# Supplementary material for: Love the one you’re with: replicate viral adaptations converge on the same phenotypic change
Source: PeerJ. 2016 Jul 19;4:e2227. doi: 10.7717/peerj.2227 (PMC4958007; doi:10.7717/peerj.2227)
Supplement: Supplemental Information 1 [file peerj-04-2227-s001.pdf]

Supplemental Materials

Love the One You're With: Replicate Viral Adaptations Converge on the Same Phenotypic Change

Craig R. Miller, Anna Nagel, LuAnn Scott, Matt Settles, Paul Joyce, Holly A. Wichman

CONTENTS

1 Supplemental Materials and Methods 2
1.1 Adaptation Experiment 2
1.2 Sampling and Sequencing 2
1.3 Fitness Assay 3
1.4 Identifying Neutral vs. Beneficial Mutations 4
1.5 Statistical Analyses 4
Selection Coefficients of Background Mutations • Reversion Probability Between Backgrounds • Between-Well Parallelism and Differences between Backgrounds • Epistasis with Background
1.6 Time to Lysis Assay and Analysis 6
Assay • Exploring Molecular Mechanisms of Delay
2 Supplemental Results 8
2.1 Quality Control 8
2.2 Fitness Assays 8
2.3 Molecular Mechanisms of Delay 9
2.4 Within-well Phylogenies 10
References 20

1 SUPPLEMENTAL MATERIALS AND METHODS

1.1 Adaptation Experiment

ID11 (GenBank accession number AY751298; Rokyta et al. 2005) is a single-stranded DNA bacteriophage of the family Microviridae. It has a genome of 5,577 bases encoding 11 genes arranged in the same way as G4 (from which it differs by $\sim 3\%$). We used the nine first-step beneficial mutations obtained by Rokyta et al. (2005) via flask-passaging. Here we adapted each of these nine genetic backgrounds in eight-fold replicate in multiwell plates for 100 passages. Three flat-bottomed 48-well plates were used per passage (Falcon non-treated tissue culture, 1.4 mL volume). We established a checkerboard pattern on each plate where the equivalent of 'black' wells were media-only negative controls (used to assess prevalence of potential contamination events) and 'red' wells contained phage. The 72 lineages were assigned at random to the 72 'red' wells. Each lineage was initiated from a plaque that was grown on *E. coli* C at 37°C (i.e., eight different plaques were used per background). In initiating the first plate, we placed 10^4 phage into their target 'red' wells, then 500 μL of freshly grown host cells at density 10^8/mL were added to all wells, including the 'black' ones. Wells were sealed with a double-layer of gas permeable membrane (Breathe-Easy, Diversified Biotech, Dedham, MA) to reduce the possibility of contamination between wells, and grown for 25 minutes in a 37°C incubator with shaking at 200 rpm.

All passages in the experiment then followed the same basic protocol. (1) Host *E. coli* C cells were grown at 37°C in a flask of ϕLB (a modified Luria-Bertani broth: 1% w/v Tryptone, 0.5% w/v Bacto yeast extract and 1% w/v NaCl) plus 2 mM $CaCl_2$ in a shaking water bath to a density of 10^8/mL. (2) At room temperature and within a UV pre-sterilized hood, 500 μL of cells were distributed into each well in each recipient plate. (3) One of the two membranes was pealed from the donor plates (i.e., plates with passaged phage). (4) A hot soldering iron was used to burn holes in one row of eight wells and a multichannel pipette was then used to transfer 5 μL of volume from the four phage wells (i.e., the 'red' wells) to their analogous wells in the recipient plate. (5) To check for contamination, 5 μL were taken using the mulitichannel pipette from each of the negative control wells (i.e., 'black' wells) and dotted on a ϕLB plate (ϕLB + 2 mM $CaCl_2$ + 7% w/v Bacto agar) pre-spread with cells in 4 mL ϕLB top agar (ϕLB + 2 mM $CaCl_2$ + 0.7% w/v Bacto agar). The row of exposed wells from the donor plate was covered with a strip of tape and the row of recipient wells covered with a sheet of sterile paper. (6) Steps 4 and 5 were repeated for each row on the paired donor-recipient plates. The recipient plate was then sealed with new membranes. (7) Steps 3-6 were repeated for each of the other two plates. (8) Plates were placed in floor shaker at 37°C orbiting at 200 rpms for 25 minutes. (9) Plates were then removed and placed on ice for 5 minutes. We repeated steps 1-9 five times per day. At the end of the day the plates were placed in the refrigerator at 4°C overnight and treated in the morning exactly the same as plates placed on ice after growth (step 9). Negative control plates were grown for 4 hours and scored for the presence of plaques. On passages 2, 20, 40, 60, 80 and 100 we did order of magnitude titering to determine very approximate titers in the wells.

1.2 Sampling and Sequencing

After 100 passages we plated each of the 72 lineage endpoints and obtained five isolates from each. This is equivalent to sampling five individual phage at random from each population. Their genomes were sequenced by a novel method that allows many small genomes to be sequenced at once using high-throughput sequencing while retaining linkage information using the Fluidigm Access Array Platform (South San Francisco, CA) and Roche 454 Genome Sequencing FLX (454 Life Sciences, Branford, CT). Each isolate was diluted 5-fold and its DNA was amplified using 24 overlapping primer pairs (Fig. 1, primer set p) and a dual barcode multiplexed strategy on a Fluidigm Access Array System. Amplified products were then combined and run on a half plate of the 454 FLX generating approximately 422K reads of mean length 423bp. Raw Roche 454 unclipped DNA sequence reads were cleaned, assigned to barcode and amplicon, and filtered in the following manner. Raw SFF files were read directly into the R statistical programming language using the R package rSFFreader (Settles et al. 2011). Using full-length sequence reads (unclipped) Cross Match (version 1.080806; parameters: minimum matches 15, minimum score 14) from the phred/phrap/consed application suite was used to identify Roche 454 adapter sequences, primer barcodes, and amplicon primer sequences. Cross Match alignment information was then read into R and processed using custom scripts to identify alignment quality, directionality, barcode assignment, and sequence quality clip points. Reads containing both forward (< 3 allowed mismatches) and reverse (< 5

allowed mismatches) adapter, barcode and amplicon primer sequences in correct orientation and expected combination were clipped of adapter sequence and processed further (approximately 385K reads). Reads were mapped to the ancestral genome using gmap (Thomas and Serban 2010) and mutations were called using samtools followed by bcftools using default parameters (Li 2011). Mutations were accepted as real only when coverage at the site where a mutation was detected ≥ 10 times and the mutation was present in $> 90\%$ of the reads (mean coverage, 46x). To avoid strong sample size effects, we removed wells from the analysis with 3 or fewer successfully sequenced isolates. This resulted in the removal of 4 wells leaving 68 in the analysis. For each of the 68 wells we then constructed parsimony trees manually to visualize the mutational relationships among the isolates.

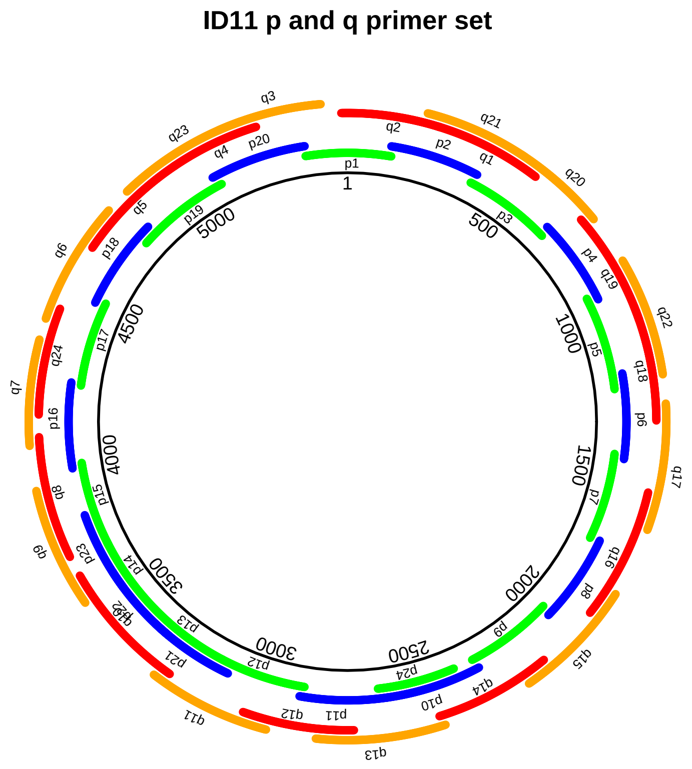

Figure 1. ID11 Fluidigm Access Array Primer Sets.
Fluidigm Access Array p and q overlapping primer sets for ID11 genome.

1.3 Fitness Assay

After completing the adaptation experiment and obtaining the sequencing results, we were surprised to find that 15 wells contained one or more isolates where the ancestral mutation had reverted to wild type. This prompted us to question whether our background mutations were beneficial as we had assumed. To address this we conducted a fitness assay that mimicked conditions in the adaptation experiment. In addition to the nine background phage we used in the experiment, we also included one with the single mutation 1910aG, that was observed in many wells. The fitness assay competed each mutation against wildtype ID11 for six passages and measured the change in the mutation's frequency. For each mutation, we seeded three wells with a 90:10 ratio of mutant/wildtype and three wells with the inverse 10:90 ratio, for a total of six replicates per mutation. Assays were initiated by diluting the mixed phage titer to 10^7/mL and then placing 10 μL into 500 μL of *E. coli c* cells that had been grown to a density of 10^8/mL. Thus, the titer of the mutation at time 0 was approximately 1.8×10^5 for the 90:10 wells and 2×10^4 for the 10:90 wells. We then passaged the wells exactly as was done in the passage experiment described above for six

transfers. Sequencing was performed on phage from passages 0, 2, 4 and 6. We used the same sequencing approach as described above except that we focused on the target mutation by using 24 times more of the primers for the segments containing the target mutations than the other primers (Fig. 1, primer set p and q).

1.4 Identifying Neutral vs. Beneficial Mutations

The identification of adaptive mutations is based on two types of evidence: parallelism and mutation location in the tree. First, when a mutation appears in more than one well independently, it is likely to be adaptive. Second, the phylogenies of each well contain information about mutations that reached moderate to high frequencies. When a mutation reaches moderate to high frequency unaccompanied by another mutation in few generations (here on the order of 10^2) in a large population (here on the order of 10^{10}), it is very likely to be beneficial. Two types of mutations bear this signature: (i) mutations on their own node sampled two or more times in a sample of just five (e.g., 1911cT and 2398cT in the example phylogenies shown in Fig. 2B), and (ii) mutations on their own node with two or more descendant lineages in the sample since these mutations were frequent enough to spawn two mutational events on them (e.g., 1911cT in Fig. 2C). Note we do not include mutations appearing at a node together because we cannot exclude the possibility that one hitchhiked to high frequency (e.g., 1910aG and 2386cT in Fig. 2C) nor do we include lone-node mutations sampled in just one isolate (e.g., 2311aG in Fig. 2C). Our back of the envelope calculations suggest that the frequency of the collective set of neutral variants in the population may become large enough that sampling any one of them once is not unlikely. Still, some of these tip mutations sampled once may be beneficial and some of the mutations at nodes with two or more mutations may be beneficial; thus, our estimates for the number and proportion of adaptive mutations and events are conservative.

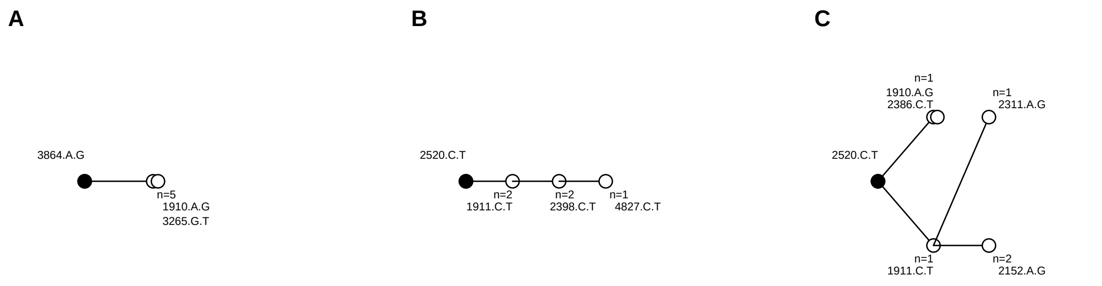

Figure 2. Example phylogenies from three wells.
Ancestral background mutation is black dot. A) All isolates fixed for same mutations is consistent with sweep dynamics. B) All mutations part of single lineage is consistent with simple clonal interference. Notice mutations 1911cT and 2398cT must have risen to moderate frequency to be sampled in two isolates each. C) Mutations on different lineages is consistent with complex interference dynamics. Notice mutation 1911cT must have risen to moderate frequency in order to serve as a background for two different subsequent mutations (2311aG and 2152aG).

1.5 Statistical Analyses

1.5.1 Selection Coefficients of Background Mutations

We estimated the selection coefficient (s) of each mutation relative to the wildtype (against which it was competed). For each well with a given mutation, we calculated the frequency of the mutation at each sample time based on the fraction of reads in which it was observed. We assumed two generations per passage such that sampling at passages 0, 2, 4 and 6 equated to generations 0, 4, 8 and 12. We then ran a

linear regression of generation against the natural log of the frequency. The slope of this line provides an estimate of s. We averaged over the six replicates to obtain a point estimate of s for each mutation. We calculated confidence intervals by using the replicates to calculate standard errors (SE), assumed normality and took the upper and lower bounds to be ± 1.96 SE.

1.5.2 Reversion Probability Between Backgrounds

We tested whether the probability of reversion is equal across backgrounds using the following likelihood ratio test: (1) calculate lnL_{null} = the sum over each background of the log-likelihood of the observed number of reversions at each background under the null (where all backgrounds have the same probability of reversion as given by the global frequency of reversions), (2) calculate lnL_{alt} = the sum over backgrounds of the log-likelihood of the same data under the alternative (where each background has a reversion probability given by the observed frequency at that background), (3) calculate $\Lambda = -2(lnL_{alt} - lnL_{null})$ and (4) determine the probability of Λ being this large or larger from the Chi-squared distribution with 8 df (number of backgrounds -1).

1.5.3 Between-Well Parallelism and Differences between Backgrounds

Denote the set of mutations found in wells i and j be M_i and M_j respectively (regardless of how many times each appeared or in what isolates). Let p_{ij} be the proportion of M_i found in M_j (i.e., the size of $M_i \cup M_j$ divided by the size of M_i) and p_{ji} be the reciprocal comparisons (i.e., the proportion of M_j found in M_i). We then define between-well parallelism as the mean of these two comparisons: $P_{ij} = P_{ji} = (p_{ij} + p_{ji})/2$. We calculated P_{ij} for every pairwise comparison on a given background, summarized by averaging and repeated for all nine backgrounds. Because P_{ij} values within a background are not independent of each other, we cannot use ANOVA to test for difference between backgrounds. Instead, we conducted the following randomization test to see if the levels of within-background parallelism were different for different backgrounds. The null in this case is that the level of parallelism does not depend on background. Notice that a more general null that produces no differences among backgrounds is when background does not effect the probability of a mutation arising (i.e., no epistasis with the background mutations). We used this more general null in the test described next. Failure to reject the more general null implies that the more specific null cannot be rejected either. For the real data, we took each background, calculated P_{ij} for all pairs of wells within that background and repeated for each background. On this within-background data, we calculated the total sum of squares (SS_{total}), the background sum of squares (SS_{back}) and calculated the proportion of explained variation as $R_{real} = 1 - SS_{back}/SS_{total}$. Consistent with our null model, we then randomly reassigned all mutations to wells, but did so holding the total number of mutations in each well fixed to the observed values, and avoided assigning the same mutation to the same well more than once. We repeated this 100 times. Each time, we repeated the above calculation on the randomized data to obtain R_{boot}. We then approximated the p-value as the proportion of times where $R_{real} \geq R_{boot}$.

1.5.4 Epistasis with Background

We analyzed our data for epistasis in three ways.

Method 1: Within vs. between background parallelism We first compared parallelism calculated within backgrounds vs. between backgrounds. To do this we took the real data, concatenated all within-background P_{ij} values and averaged to get $\bar{P}_{within}$, concatenated all between-background P_{ij} values and averaged to get $\bar{P}_{between}$ and defined our summary statistic as their ratio: $\bar{P}_{w/b(real)} = \bar{P}_{within}/\bar{P}_{between}$. To get the distribution of this ratio under the null, we assumed a null where background does not effect where mutations arise. Thus we randomized the associations between mutations and background while maintaining the number of observed mutations in each well and the number of observations of each mutation. For each of 100 bootstrap replicates we took the randomized data and calculated $\bar{P}_{w/b(boot)}$. We approximated the p-value as the proportion of times $\bar{P}_{w/b(boot)} > \bar{P}_{w/b(real)}$.

Method 2: Mutation-background association We used the within vs between test on parallelism (method 1) because it fits logically into the paper's theme. However, as a method testing whether there is an association between background and mutation, it is less powerful than the likelihood ratio test (LRT) we present next. The LRT also has the advantage of not only showing if an association exists, but, when

one does, of revealing which mutations are most responsible for it. Let w_b be the number of wells in the experiment initiated on background b, w_{ib} be the number of times mutation i arose among these w_b wells, and let p_{ib} be the probability that mutation i arises in a well with background b. We model w_{ib} as a binomial random variable with parameters w_b and p_{ib}. Under the null, background has no influence on the probability p_{ib}; the log-likelihood of the data, $lnL_{null(i)}$ is maximized by using the global frequency of mutation i among the 68 wells. Under the alternative model, background affects p_{ib} and the log-likelihood $lnL_{alt(i)}$ is maximized using the observed frequency of mutation i among the w_b wells (i.e., w_{ib}/w_b). We sum over all mutations to obtain the likelihood of the data under each model: lnL_{null} and lnL_{alt}. Using the real data, we calculate the test statistic Λ as the difference: $\Lambda_{real} = lnL_{alt} - lnL_{null}$. To test for significance, we bootstrap randomized the mutations across backgrounds 1000 times, calculated Λ_{boot} each time and obtained approximate p-values as the proportion of bootstraps where $\Lambda_{boot} > \Lambda_{real}$. To determine which mutations were most important, we returned to the real data and calculated the log-likelihood difference for each mutation (i.e., $\Lambda_{real(i)} = lnL_{alt(i)} - lnL_{null(i)}$) and ranked from largest to smallest. We then began removing mutations in a cumulative fashion from largest $\Lambda_{real(i)}$ downward, each time repeating the bootstrap randomization test to determine significance, and continuing until the p-value was no longer < 0.05. The largest set for which $p < 0.05$ was used to define the mutations that show significant background associations.

Method 3: Epistasis among de novo mutations The third test examines epistasis between pairs of *de novo* mutations. We were interested in whether any pairs of mutations co-occur more or less often than expected by chance. Here co-occurrence is defined as appearing in the same isolate, not simply in the same well. For the analysis, we removed all singletons and reversions. We then considered all pairs of remaining mutations and calculated the number of independent wells in which the two mutations were found in an isolate together. Being observed together in more than one isolate from a well was considered one observation. To assess significance, we bootstrap randomized the observed set of mutations across wells and isolates. To do this, we proceeded well by well, took each mutation found in that well and replaced it with a random mutation in every isolate where it occurred. Thus, our randomization process retained the genealogical patterns from the real data. After randomizing the data, we repeated the calculation of co-occurence for every pair of mutations. After 100 bootstrap replicates, we calculated approximate p-value for each pair of mutations, ij, by locating the real number of co-occurrences ($C_{ij(real)}$) in the bootstrap distribution of the same comparison ($C_{ij(boot)}$). Specifically, for small $C_{ij(real)}$ values (i.e., $C_{ij(real)} \leq \bar{C}_{ij(boot)}$), we took the p-value to be twice the proportion of bootstraps where $C_{ij(real)} \leq C_{ij(boot)}$; for large $C_{ij(real)}$ values (i.e., $C_{ij(real)} \geq \bar{C}_{ij(boot)}$), we took the p-value to be twice the proportion of bootstraps where $C_{ij(real)} \geq C_{ij(boot)}$. Because we were using this method to probe for potentially interesting patterns and not to draw firm conclusions, we did not perform a multiple test correction. However, we did limit the most obvious source of false positives by excluding mutation pairs with $p < 0.05$ that co-occurred just once (i.e., cases where $C_{ij(real)} = 1$ and $\bar{C}_{ij(boot)} << 1$).

1.6 Time to Lysis Assay and Analysis

1.6.1 Assay

The above analysis of epistasis among de novo mutations (method 3) in conjunction with evidence in the literature led us to hypothesize that a number of the mutations that arose in the experiment delayed lysis. We tested this hypothesis on a subset of mutations. To do so we identified four putative lysis-delaying mutations (1910aG, 1911cT, 2131cT and 2134tC) where we had one or more backgrounds both with and without the individual mutation in our set of sequenced isolates. We then assayed lysis time for these phage using the following procedure. Phage were recovered from either 4°C liquid stocks or deep freeze storage and plaqued out as described above. Single isolates were picked into 750 μL ϕLB, extracted with 50 μL $CHCl_3$ to remove hosts and stored at 4°C. Genomes of the plaque-purified isolates were re-sequenced to verify genotypes. To do this, overlapping halves of the circular phage genome were amplified by PCR and each half was Sanger-sequenced with 6-7 primers using Applied Biosystems Big Dye Terminator Cycle Sequencing chemistry (Life Technologies, Grand Island, NY). Reactions were visualized on an ABI 3130 automated sequencer. The trace files were compiled with Lasergene program SeqMan (DNAStar, Madison, WI) and contigs were aligned with ID11 and checked manually for the expected mutations. The sequence-verified isolates were then diluted and plated out and three single plaques of each mutant were picked for replicate assays.

Assays for lysis time were conducted in 125-mL Erlenmeyer flasks in a 37°C water bath shaking at 200 rpm with the water level at 3 cm above the platform. In order to reduce the confounding effect of secondary infection, assays were performed with two flasks – attachment and lysis. For each assay, two flasks containing ϕLB + 2 mM $CaCl_2$ were warmed in the bath as the *E. coli* C host cells were growing to log phase in a separate flask. When the cells reached an A600 O.D. of 0.300, they were aliquoted into each attachment flask so the final volume was 10 mL and their concentration was 7.5×10^7 cells/mL. 10,000 phage (10^4/ml) were added and allowed to attach to the host cells for 7 minutes, then 10 μL were transferred to the lysis flask containing 10 mL warm ϕLB + 2 mM $CaCl_2$ (a 10^{-5} dilution). This dilution greatly reduces the rate that phage released by the initial bursts encounter hosts and thereby reduces secondary infections. 500 μL samples were taken once per minute over the empirically determined lysis time window for each phage, a period typically of 5-10 minutes. The samples were filtered through 0.2 μm PES membranes (Acrodisc, Pall Corporation, Port Washington, NY), and 100 μL was plated with 100 μL of log phase *E. coli* C in 4 mL ϕLB top agar on ϕLB plates. The dilutions and plating volumes were calibrated so that, until lysis occurred, 1 plaque was expected per plate. Timepoints with extremely high plaque counts relative to others in the same time series likely indicated lysis during sampling or filtration; these assays were repeated. Mutants were always assayed simultaneously with their appropriate background phage and replicates of the same mutant were generally assayed on different days. Because the differences between mutation and background were strikingly obvious for all mutations and all backgrounds, we did not conduct a formal time-series statistical analysis. We instead simply averaged the estimated burst size (the plate count) over replicates for each timepoint for each genotype and calculated standard errors.

1.6.2 Exploring Molecular Mechanisms of Delay

Our lysis time assay confirmed that all four of the mutations we studied delay lysis (see Results and Discussion in main paper). We suspect that all of the delay is being driven by reduced expression of the lysis protein, protein E. We then wondered, what molecular mechanisms might lead to down-regulation of protein E? The mutations and potential delay mechanism(s) fall into two groups that we treated separately: D-promoter mutations and mutations within gene(s) D and E.

D-promoter Mutations: Nine different mutations arose in the -10 and -35 regions of the D-promoter and two more arose between these regions. Collectively, these mutations were common (in 62 of 68 wells) and yet never co-occurred (Fig. 8 of main paper). We suspect that their repulsion comes from the fact that they are all doing the same thing: delaying lysis. We further hypothesize that they are doing this by altering the binding affinity of the RNA Polymerase at the D-promoter site. To test this, we used the thermodynamic promoter model from Brewster (2012) to predict how our observed mutations would alter RNA Polymerase binding affinity. This model was developed within *E. coli*. We specifically used their energy matrix (file SI Text S2) derived from Kinney et al. (2010) to plot the binding energy of the background and each mutation.

Mutations in Genes D and E: We identified 38 mutations in the gene D/E open reading frame and partitioned them into two groups: (i) 11 mutations that are mutually exclusive of the D-promoter mutations and therefore that we suspect delay lysis and (ii) 27 found with D-promoter mutations that we suspect do not delay lysis. We then tested four hypotheses, each which might generate in differential lysis protein expression between the two groups: (1) & (2) that the 11 putative lysis delay mutations change codon usage to less preferred codons when translating gene D (1) or gene E (2) while the other group does not; (3) that the 11 putative lysis delaying mutations cause ribosomal pausing by creating anti-Shine Dalgarno-like motifs while the other group does not; and (4) that the 11 putative lysis delaying mutations significantly alter the stability of the mRNA and thereby affect translation. To assess the effects on codon usage, we used the codon usage values for 40 highly expressed genes in *E. coli* from Sharp et al. (2010), calculating the difference in usage between the wild type and the mutant codon for each mutation. Because genes D and E are overlapping but have out-of-register reading frames, we did this separately for the two genes. Ribosome Shine-Dalgarno (SD) affinities were calculated for the background and mutant sequences after Li et al. (2012) for a sliding 6-nucleotide window across the D/E transcript using values provided by Gene-Wei Li (personal communication). The effect of each mutation was quantified as the largest difference in affinity (in either direction) between the wild type and the mutant among the 6-base

windows covering the mutation. Finally, transcript stability was quantified as the minimum free energy (MFE) of the entire transcript based on the RNAfold program within the Vienna RNA Package (version 2.1.8; Lorenz et al. 2011). The effect of each mutation was taken as the difference between wild type and the mutant MFE. For all hypotheses, we conducted a simple t-test (with unequal variances) comparing mutational effects of the molecular quantity (Δ codon usage, Δ SD-affintity, Δ MFE) between the set of 11 putative lysis delaying mutations vs. the set of 27 putative non-lysis delaying mutations.

2 SUPPLEMENTAL RESULTS

2.1 Quality Control

Mean coverage was 42x (by isolate sd = 18.5, by site sd = 21.5). We failed to get usable sequence on a 257 base pair region in gene H (bases 4871-5128) in most samples and therefore excluded this region for all samples. Our goal was to obtain five sequenced isolates for each of the 72 wells in the experiment. We obtained 2, 3, 4, 5 and 6 sequenced isolates in 4, 0, 3, 63 and 2 wells, respectively . To avoid strong sample size effects we removed the four wells with just two sequenced isolates. Contamination was a concern in our experiment because adaptations involved 100 transfer events for each of three 48-well plates using a multichannel pipette. Potential contamination events were identified by four criteria: (1) the expected background mutation was missing from one or more isolates from the well, (2) in those isolates, one of the other (unexpected) background mutations appeared, (3) the putative contamination isolates carried other mutations (besides the unexpected background mutation) that linked them to another well on the same plate and (4) reversions were otherwise rare in wells with the background we expected to find in the well. By these criteria, three wells may have had contamination events. In one case on background F355 we had one of five observed isolates that clearly matched a genotype that was common on another background. Whether this was an actual contamination event or a mistake during sample processing is not known, but we simply removed the single isolate leaving 4 others. There are two other cases where contamination criteria 1 and 2, but not 3 or 4, were met. Here it was impossible to know if the results are real evolution or represent contamination. Given the overall scarcity of evidence for contamination we suspect these represent real evolution. We therefore present our analysis with the wells included, but we also reran the analysis without them to confirm that their inclusion or exclusion has no qualitative effect on our results and conclusions.

2.2 Fitness Assays

Because endpoint sequencing revealed that 15 wells contained reversions, we came to suspect that our background mutations were not beneficial under the passage conditions. We therefore conducted a short-term fitness assay. The results confirm our suspicions (Fig 3), suggesting that the ancestral mutations in the fitness assay were either deleterious (F355, F322, J20, F416 and F421) or near neutral (F5, J15, F182 and F178). The results thus indicate we did not execute the experiment we intended. Instead, we initiated replicate adaptation on nine different first-step backgrounds, but not first-steps that selection would likely have pursued on its own. Furthermore, it is clear that fitness assay results are not entirely consistent with the larger replicate adaptation experiment. For example, mutation J20 appears deleterious in the fitness assay. By contrast, in wells that began with the F416 background we see reversion in 7 of 8 replicates; in all 7 cases mutation J20 arose. In one of these cases the parsimony tree indicates the reversion preceded the acquisition of J20 (in the other cases order cannot be determined). Thus, the replicate adaptation data suggests that J20 was beneficial. We cannot reconcile these results except to point out that the fitness assay was done months later and by a different person than the original adaptation passages. Nevertheless, these assays confirmed our suspicion: fitness effects differed substantially between the flask and the microtiter environments.

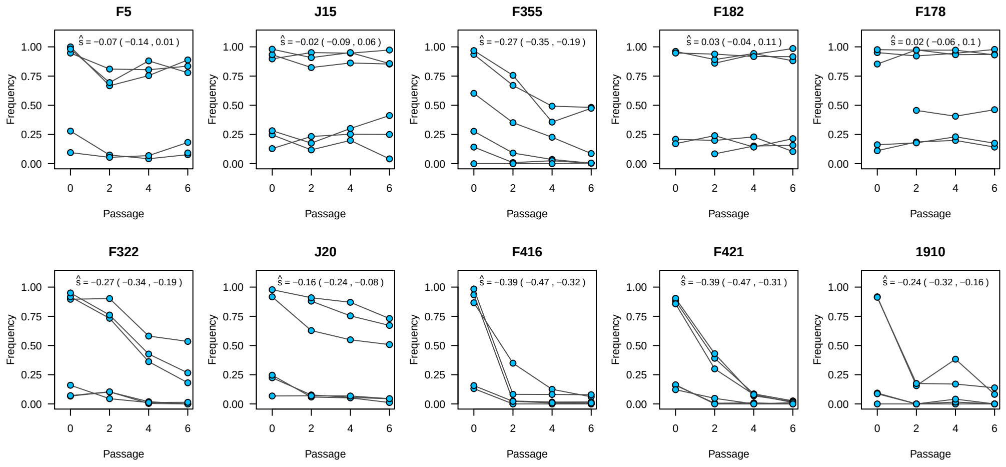

Figure 3. Background mutations were generally near neutral or deleterious, not beneficial. Plots show frequency changes over six passages for the nine ancestral backgrounds plus mutation 1910 (identified above panels). Passaging of each genotype was replicated six times; each replicate is connected by lines. Estimated selection coefficients and 95% confidence intervals are inset.

2.3 Molecular Mechanisms of Delay

D-promoter Mutations: Fig. 4 shows the effect that each D-promoter mutation is predicted to have on RNA Polymerase binding affinity based on the model of Brewster et al. (2012). Nine of the eleven total mutations reduce affinity and five of those have effects between +0.95 and +1.8 k_BT. These values correspond to between 2.6 and 6.0-fold reductions in the predicted expression level ($e^{0.95} = 2.6$; $e^{1.8} = 6.0$). However, the mean squared error of their model was 1 k_BT unit–the same order as our predicted effect size. More troubling is the fact that the two D-promoter mutations observed frequently have small and inconsistent effects on stability: 1910aG is observed 53 times and changes affinity +0.17 k_BT and 1911cT is observed 27 times and changes affinity -0.10 k_BT. Hence, the thermodynamic model of Brewster et al. (2012) does not illuminate what the D-promoter mutations are doing. It could be that the relationship between down-regulation of protein E and lysis time is below the sensitivity of their model (i.e., a two- or three-fold reduction in expression has a huge effect on lysis time). Alternatively, it is possible that other molecular mechanisms are at work such as transcription factors or secondary structures.

Mutations in Genes D and E: Our analysis found relatively little evidence for differences in these molecular traits between the 11 D/E mutations in repulsion with the D-promoter mutations and the 27 that are not (Fig. 5). Mutations in our putative lysis-delay group did not change codon usage in gene D, the Shine-Dalgarno affinities, or the transcript stability in ways consistently different from the other group of mutations. In particular, we noted that the two mutations known to delay lysis (2131cT and 2134cT—denoted *a* and *b* in the figure) show no pattern of effect by any measure. The one significant result is that mutations in gene E change usage to less preferred codons ($p = 0.004$), but because neither 2131cT nor 2134cT is in gene E, this result does not explain the lysis delay observed in these two phage. Thus, the molecular mechanism allowing these two mutations and perhaps others to delay lysis remains largely unknown. It is conceivable, but far from proven, that mutation to low-use codons in gene E contributes in some cases.

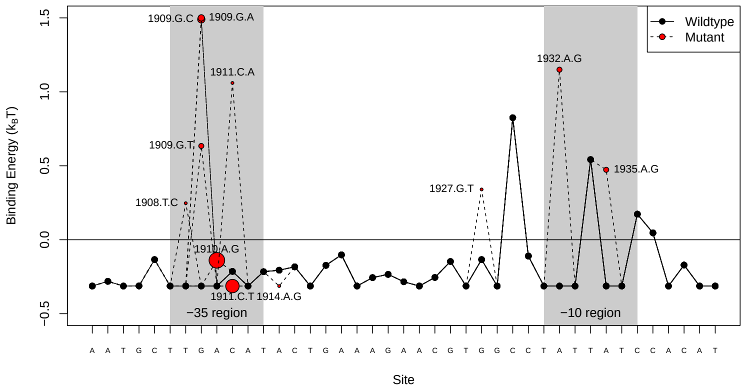

Figure 4. Predicted mutational effects on binding energy profile across the D-promoter. Plot shows the predicted effect of each D-promoter mutation on the binding affinity of RNA polymerase based on the thermodynamic model of Brewster et al. 2012. Mutations are scaled by the number of wells they appeared in. The wildtype sequence is given along the x-axis.

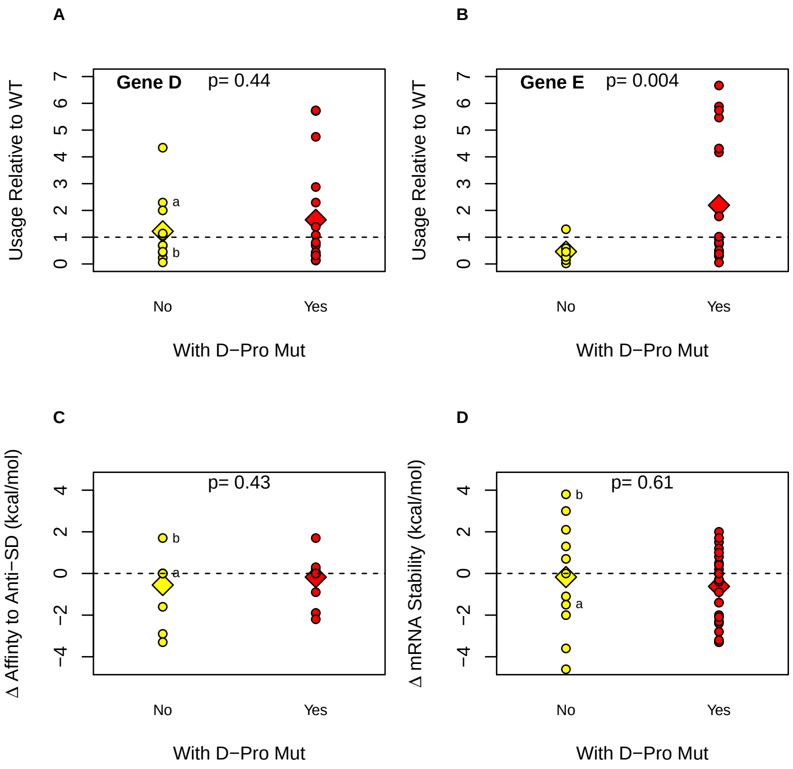

Figure 5. Comparison of molecular properties for mutations in the genes D/E transcript in repulsion with D-promoters mutation (yellow) vs. those found with D-promoter mutations (red). (A) Mutant codon usage relative to wild type in gene D. (B) Mutant codon usage relative to wild type in gene E. (C) Maximum change in Shine-Dalgarno affinity about the mutation site. (D) Change in minimum free energy (stability) of the transcript. The assayed mutations 2131cT and 2134cT are denoted with *a* and *b* respectively.

2.4 Within-well Phylogenies

The following nine figures present the relationships among sampled isolates within each well, sorted by background (Fig. 6 - Fig. 14).

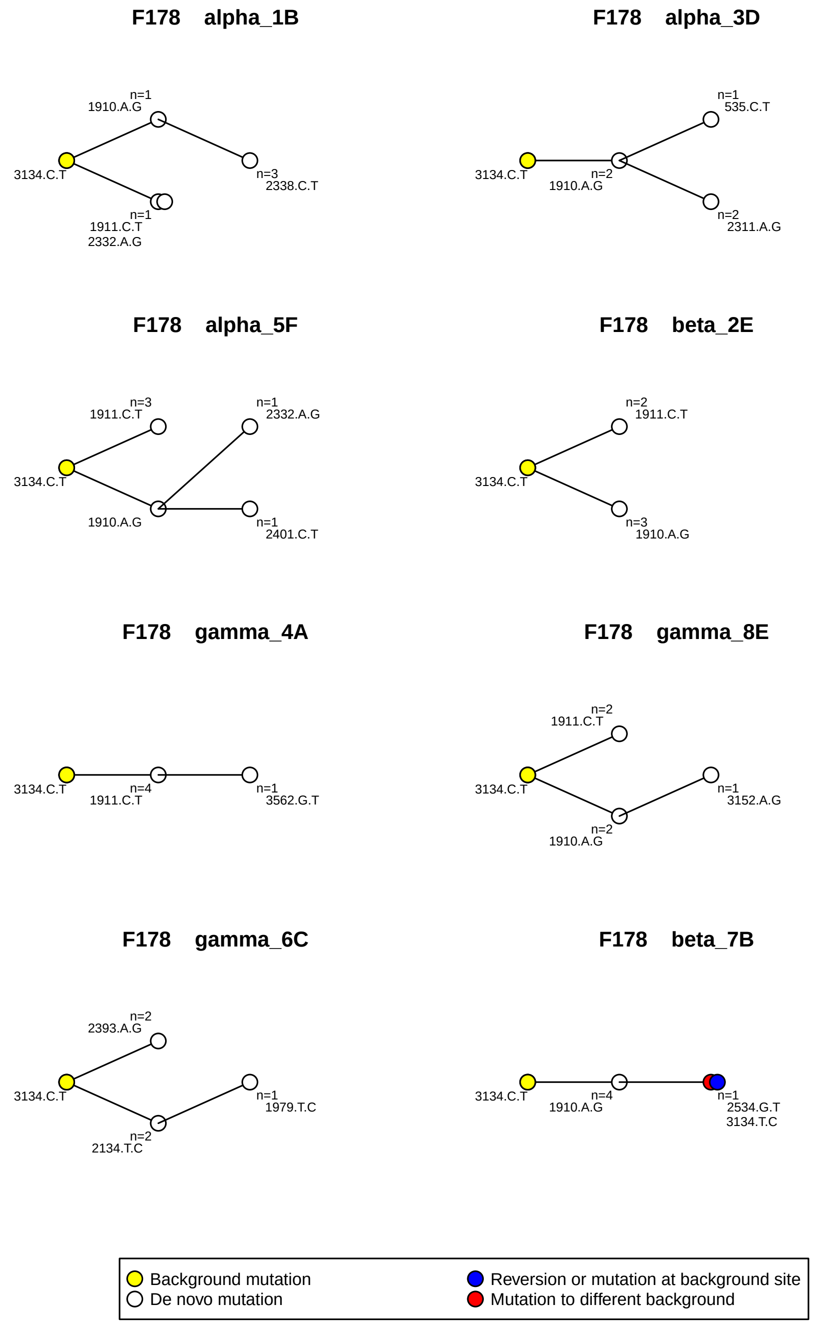

Figure 6. **Phylogenies for wells seeded with background F178.**
Each panel corresponds to one well. Panel names indicate the passaging plate (the three plates were named alpha, beta and gamma) and the well address on the plate (plate rows were A-F and columns were 1-8).

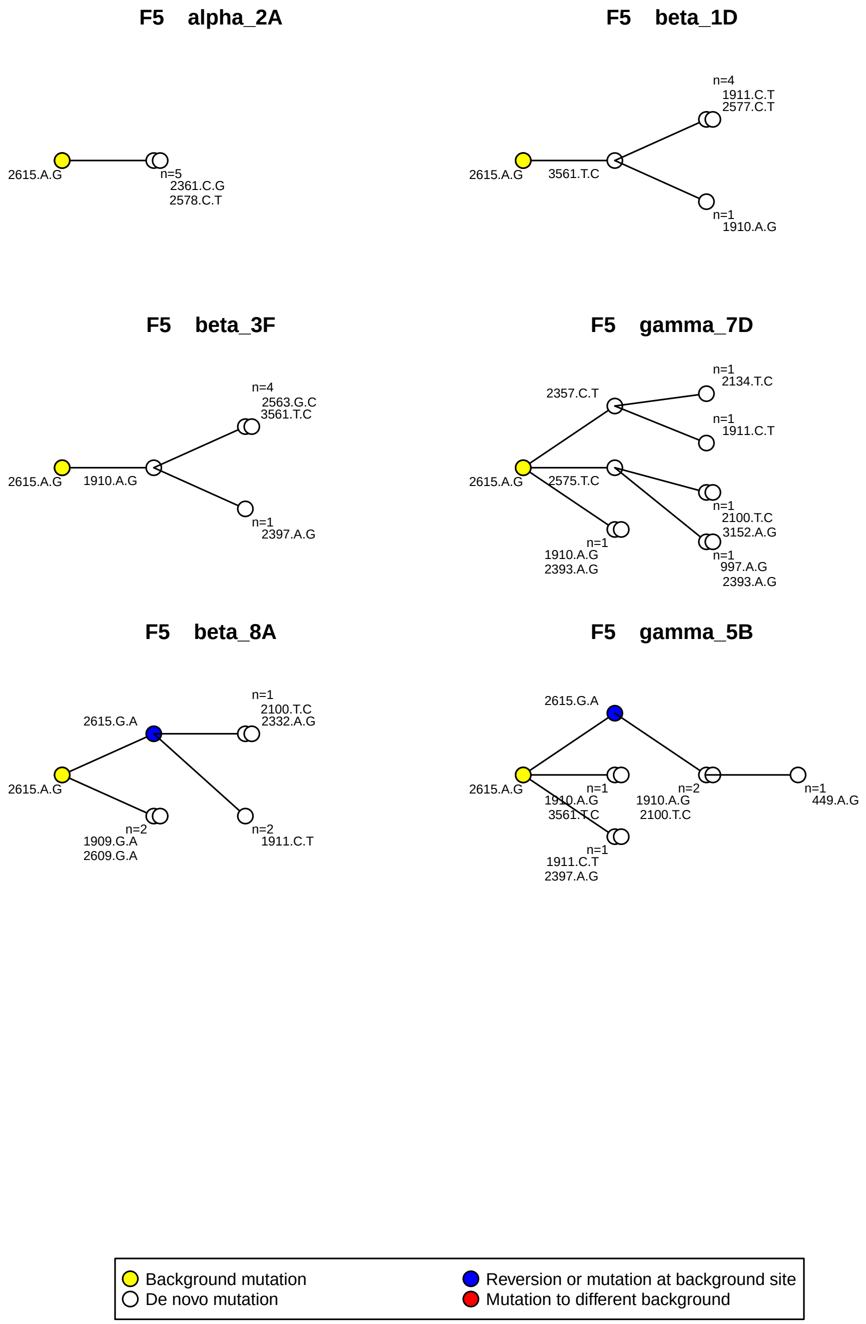

Figure 7. Phylogenies for wells seeded with background F5.
Note well gamma_7D has homoplasy and one possible parsimony tree is shown. Two wells have been removed due to quality control issues (see Materials and Methods).

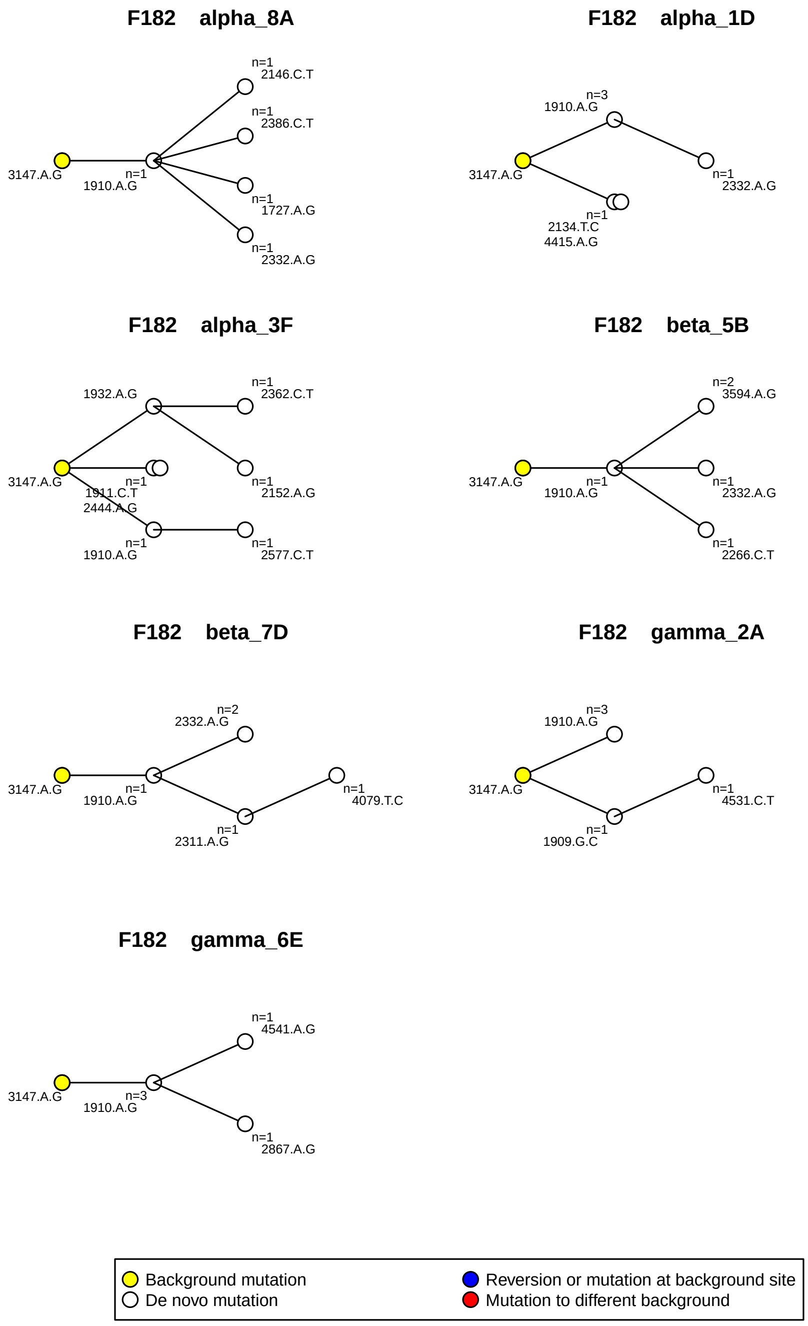

Figure 8. **Phylogenies for wells seeded with background F182.**
Note that one well has been removed due to quality control concerns.

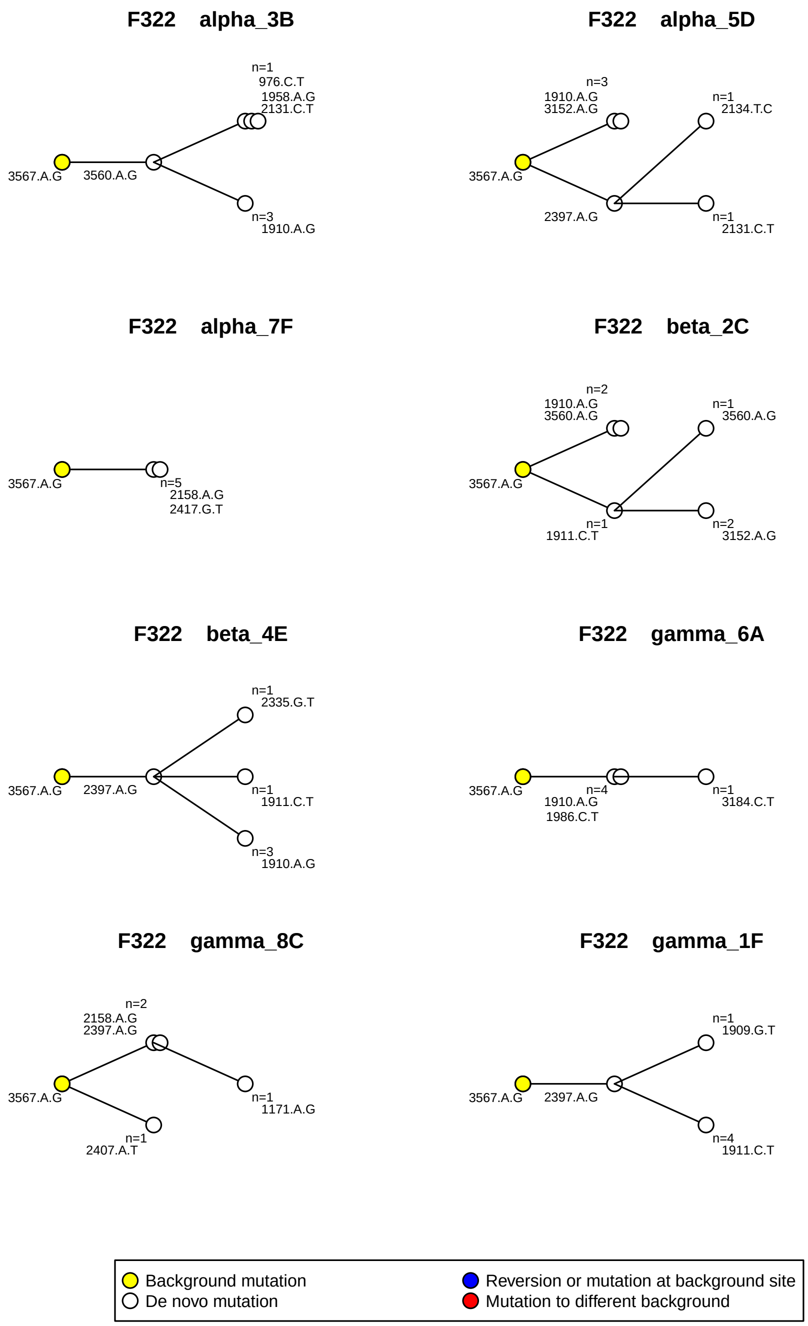

Figure 9. Phylogenies for wells seeded with background F322.

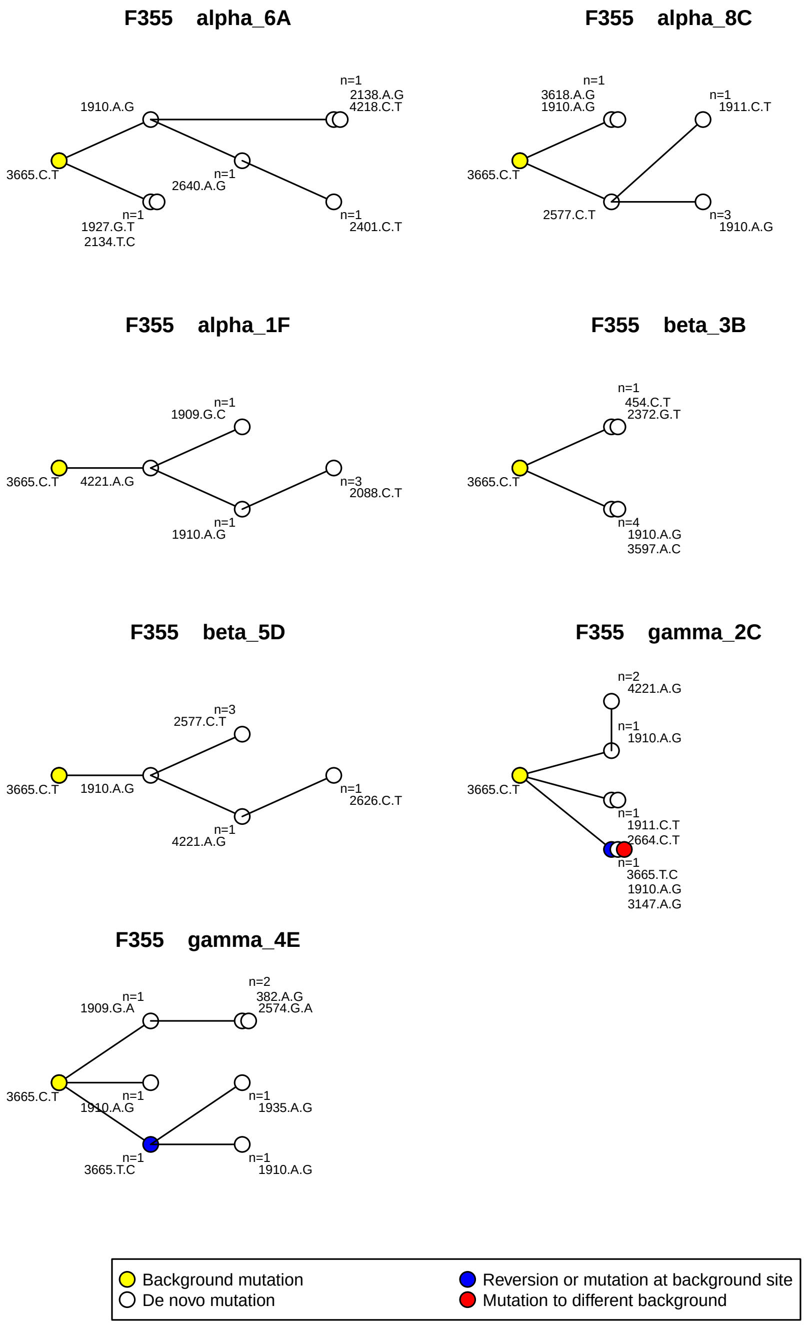

Figure 10. **Phylogenies for wells seeded with background F355.**
Note that one well has been removed due to quality control concerns.

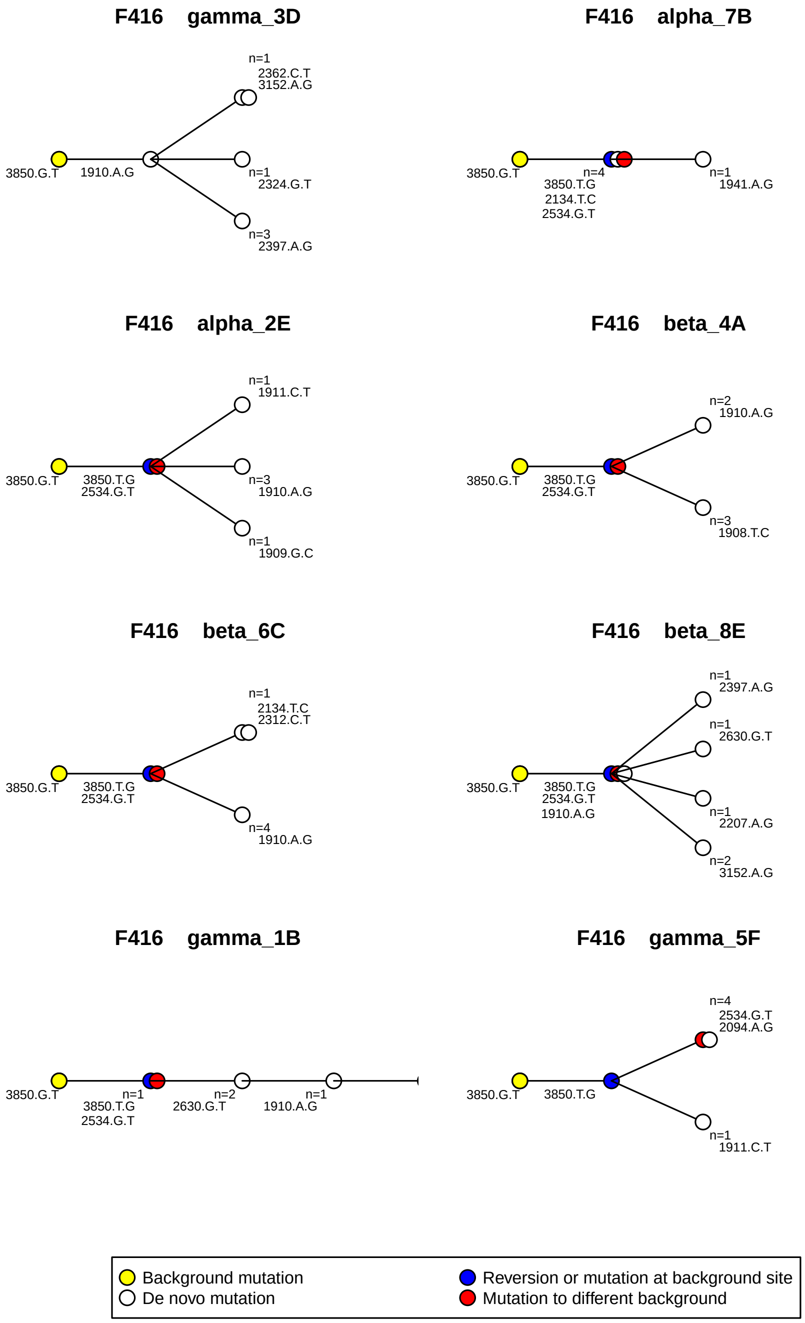

Figure 11. Phylogenies for wells seeded with background F416.

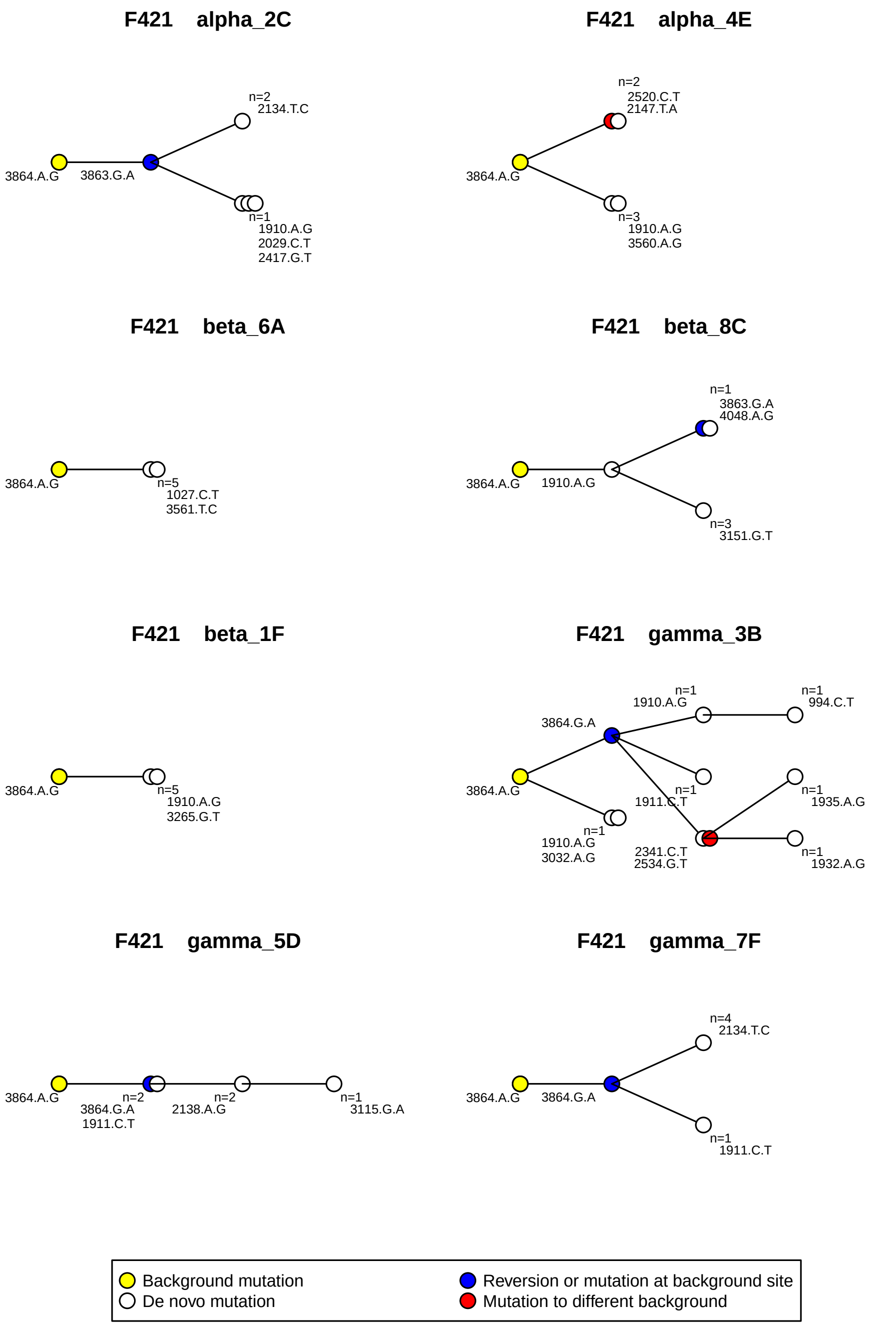

Figure 12. Phylogenies for wells seeded with background F421.

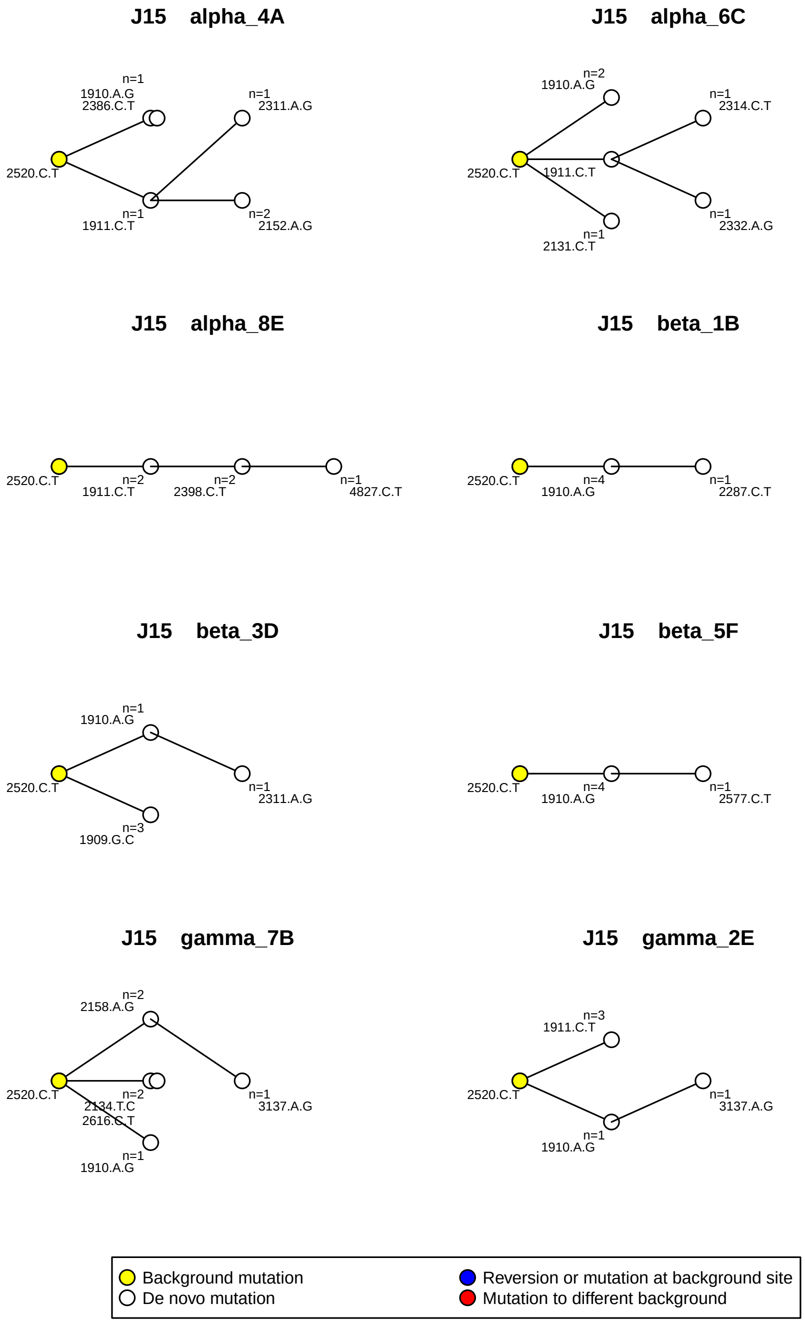

Figure 13. Phylogenies for wells seeded with background J15.

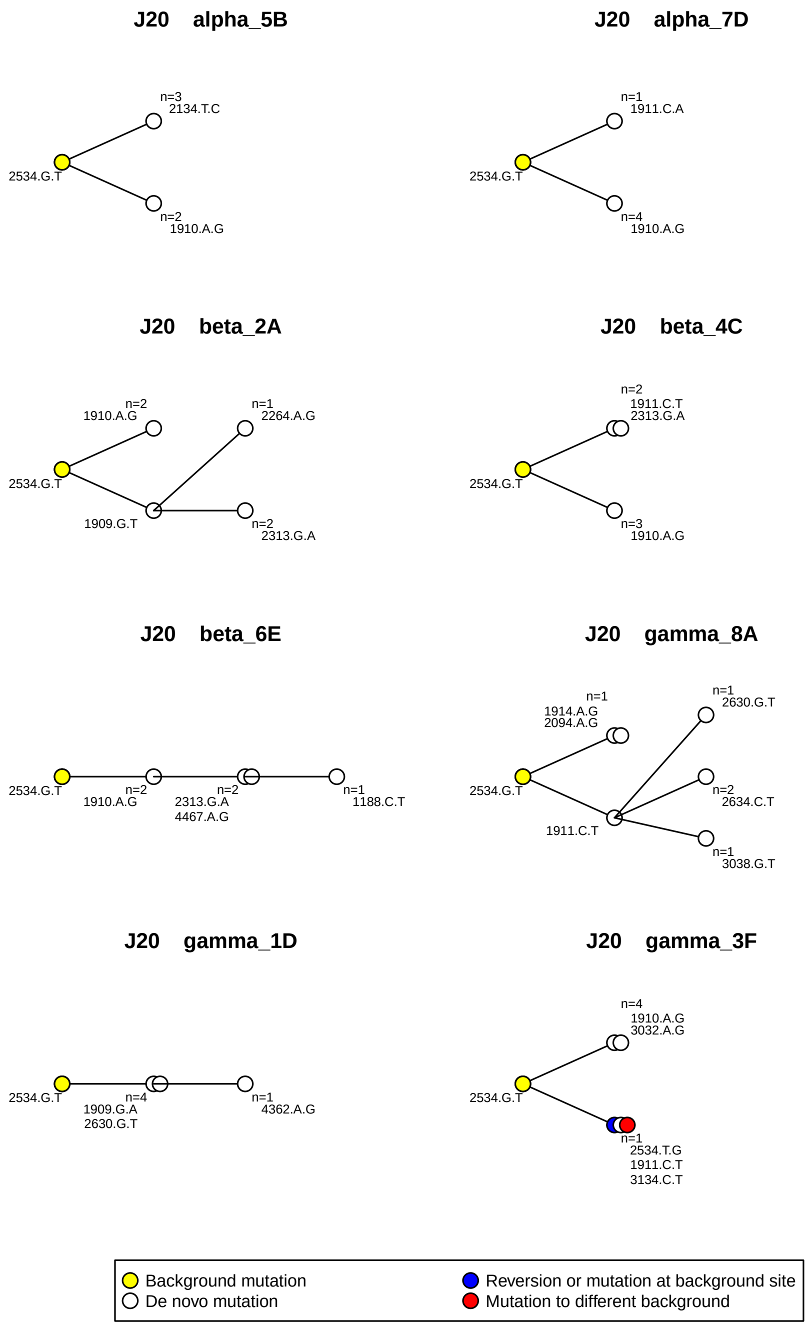

Figure 14. **Phylogenies for wells seeded with background J20.**

REFERENCES

Brewster, R. C., Jones, D. L., and Phillips, R. (2012). Tuning promoter strength through rna polymerase binding site design in ¡italic¿escherichia coli¡/italic¿. *PLoS Comput Biol*, 8(12):e1002811.

Kinney, J. B., Murugan, A., Callan, C. G., and Cox, E. C. (2010). Using deep sequencing to characterize the biophysical mechanism of a transcriptional regulatory sequence. *Proceedings of the National Academy of Sciences*, 107(20):9158–9163.

Li, G.-W., Oh, E., and Weissman, J. S. (2012). The anti-shine-dalgarno sequence drives translational pausing and codon choice in bacteria. *Nautre*, 484:538?541.

Li, H. (2011). A statistical framework for snp calling, mutation discovery, association mapping and population genetical parameter estimation from sequencing data. *Bioinformatics*, 27(21):2987–2993.

Lorenz, R., Bernhart, S., zu Siederdissen, C. H., Tafer, H., Flamm, C., Stadler, P., and Hofacker, I. (2011). Viennarna package 2.0. *Algorithms for Molecular Biology*, 6:26.

Rokyta, D. R., Joyce, P., Caudle, S. B., and Wichman, H. A. (2005). An empirical test of the mutational landscape model of adaptation using a single-stranded DNA virus. *Nature Genetics*, 37(4):441–444.

Settles, M. L., Hunter, S., Sarver, B., Zhbannikov, I., and Cho, K. (2011). rsffreader: rsffreader reads in sff files generated by roche 454 and life sciences ion torrent sequencers. *Bioconductor, R package version 0.18.0.*

Sharp, P. M., Emery, L. R., and Zeng, K. (2010). Forces that influence the evolution of codon bias. *PTRSB*, 365:1203–1212.

Thomas, W. D. and Serban, N. (2010). Fast and snp-tolerant detection of complex variants and splicing in short reads. *Bioinformatics*, 26:873–881.
