# Supplementary material for: Love the one you’re with: replicate viral adaptations converge on the same phenotypic change
Source: PeerJ. 2016 Jul 19;4:e2227. doi: 10.7717/peerj.2227 (PMC4958007; doi:10.7717/peerj.2227)
Supplement: Supplemental Information 2 [file peerj-04-2227-s002.html]

Data Analysis


# Data Analysis

#### *Craig Miller*

#### *April, 2015*

## R Markdown for *Love the One You’re With: Replicate Viral Adaptations Converge on the Same Phenotypic Change*

##### by Craig R. Miller, Anna Nagel, LuAnn Scott, Matt Settles, Paul Joyce, Holly A. Wichman

## OVERVIEW

This markdown file has all the R code to do the analysis in the paper and the supplement as well as create the figures in both documents. Note that the processing of raw data was performed by Matt Settles; the files and scripts involved therein are provided in the *RawDataAnalysisFiles* folder as part of the Supplemental Materials. The functions for this markdown are found in *Analais Functions Replicate Adaptations Study.R* which is sourced in the first block of code. All the files necessary to running the analysis have been put into the *Markdown* folder where this file resides. You will need redefine the pathway to the Markdown folder using the setwd() command in the first code block below. The code also expects that data will be in the *Data* folder and other files that direct modeling in the *Model\_input\_files* folder.

## ISOLATES SEQUENCE ANALYSIS

From here down to MUTATION ANALYSIS, the code is about alinging the reads against the ancestor and finding the mutations. Note that I did this anlaysis and Matt Settles used alternative methods of aligning sequenes and identifying reads (see his anlaysis in *RawDataAnalysisFiles*). I confirmed that our two methods of analysis yielded identical sets of mutations.

#### Load Packages and set working directory

```
library(Biostrings)     # to install: (1) source("http://bioconductor.org/biocLite.R")
library(ShortRead)
setwd("~/Dropbox/Craig_Work/Project--Replicate Adaptation/Markdown")
source("Analysis Functions Replicate Adaptations Study.R")
```

#### Control workflow using booleanes

```
  analyze.isolates <- FALSE       # Extraction of mutations. Slow. Generally set to false and read in mutations.
  translate.mutations.bool <- FALSE    # Translate mutations (take a minute or so) or just read in.
  calculate.parallelism.bool <- TRUE    # Does so if true (takes a few minuts); otherwise it reads results in and crates plots.
  bootstap.win.bet.parallelism.bool <- FALSE
  bootstrap.cocount.sig.bool <- FALSE
```

#### ID11 Genome Inputs

```
  ID.11 <- readBStringSet(filepath=paste(getwd(), "/Model_input_files/ID11_seq.txt", sep=""), format="fasta")
  #ID.11 <- readDNAStringSet(filepath="ID11_seq.txt", format="fasta")
  ID.11.genes <- read.table(file=paste(getwd(), "/Model_input_files/ID11_gene_startstop.txt", sep=""), header=TRUE)
  anc.sites <- read.table(file=paste(getwd(),"/Model_input_files/anc_states_sites.txt", sep=""), header=TRUE)   # note it was  "F421 3863.G.A 3863" but I changed to F421 3864.A.G 3864 b/c it is the mutation in 3864 that appears
  
  ID11.features <- read.table(file=paste(getwd(),"/Model_input_files/ID11.gene.features.txt", sep=""), header=TRUE)
  n.genes <- length(ID.11.genes$gene)
  gene.names <- ID.11.genes$gene
  wraped.genes <- as.numeric(apply(ID.11.genes, 1, function(x) x[3] < x[2]))
```

#### Read in, define outputs, set up containers

Note you must toggle anlayze.isolates <- TRUE below if you actually want to run this analysis. It takes a minute or two per sample. And there are 385 isolates. So if you run it all it takes several hours. Also, at top here you define the names of the files that hold the observed mutations and the coverage per site. You can skip running this code by simply reading the results in below.

```
  mut.fileout <- "Data/Mutation_output_file.txt"
  cov.fileout <- "Data/Coverage_output_file.txt"

    if (analyze.isolates == TRUE){
      good.reads <- readDNAStringSet(paste(getwd(), "/Data/good_reads(Oct2013)_ISO.fasta", sep=""), format="fasta")   # In /Good_reads_both_runs/ this should have all non-frequencey (fitness) reads
      good.data <- read.table(file=paste(getwd(),"/Data/good_data_ISO.txt", sep=""), header=TRUE)
      sample.sheet <- read.table(file=paste(getwd(), "/Data/sample_assignment_both_runs_182_to_178_corrected.txt", sep=""), header=TRUE)  #(the 182 to 178 is a correction of 2 mislabelled samples)
    
      amps.to.split <- c("a22", "b23")   # for phiX174
      amps.to.split <- "p1"   # for ID.11
      list.of.amps <- levels(good.data$Amplicon)
      proj <- "ID11"      # this selects the samples to analyze.  Set up to analyze by phage (but could easily be changed to anlayze by inivestigator, project, etc.)  OR #proj <- "phiX174"
      proj.rows <- which(sample.sheet$phage==proj)
      n.samps <- length(proj.rows)
      n.bases <- 15    # this sets length of window from end & beg of genome to use in alignments for finding which amplicons span this.  
      first.bases <- substring(ID.11, 1, n.bases)
      last.bases <- substring(ID.11, width(ID.11)-(n.bases-1),)
      length.i <- width(ID.11)
      wraped.genes <- as.numeric(apply(ID.11.genes, 1, function(x) x[3] < x[2]))
      isolate.cutoff <- 0.1    #  sets freq of mutations in isolate for which mutations will be checked for translational changes.
      pop.cutoff <- 0.1

      #Create containers

      results <- vector("list", length=n.samps)
      results.ins <- vector("list", length=n.samps)
      results.dels <- vector("list", length=n.samps)
      results.all.ins <- matrix(nrow=1, ncol=6, data=NA)
      colnames(results.all.ins) <- c("samp.i", "samp.name", "mutation", "count", "n.reads", "p.reads")
      results.all.ins <- as.data.frame(results.all.ins)
      results.all.dels <- matrix(nrow=1, ncol=6, data=NA)
      colnames(results.all.dels) <- c("samp.i", "samp.name", "mutation", "count", "n.reads", "p.reads")
      results.all.dels <- as.data.frame(results.all.dels)
      results.all <- matrix(nrow=1, ncol=21)
      colnames(results.all) <- c("samp.i", "samp.name", "mutation", "count", "n.reads", "p.reads", "genome.site", "base.anc", "base.mut", "Gene.1", "AA.1.site", "AA.1.anc", "AA.1.mut", "Gene.2", "AA.2.site", "AA.2.anc", "AA.2.mut", "Gene.3", "AA.3.site", "AA.3.anc", "AA.3.mut")
      results.all <- as.data.frame(results.all)
      #positions.to.samp <- seq(1, width(ID.11), by=100)
      positions.to.samp <- seq(1, width(ID.11))
      counts.by.position <- matrix(nrow=1, ncol=length(positions.to.samp))
      colnames(counts.by.position) <- positions.to.samp
      #counts.by.position <- as.data.frame(counts.by.position)
    }
```

#### Process Samples to Find Mutations

(1) Define samples to analyze; (2) call functions that anlzyzes each, (3) outputsto mut.fileout and cov.fileout To analyze all 385 isolates from Anna, define proj.rows <- which(sample.sheet$Investigator == “Nagel”) (see code below) Also, note it takes a minute or two per sample.

```
  if (analyze.isolates == TRUE){
    # (1) Define the samples to analyze -> proj.rows
    reversion.wells <- "beta_7B"
    #reversion.wells <- c("alpha_4C", "alpha_6E", "beta_8A", "gamma_5B", "beta_7F", "gamma_2C", "gamma_4E", "gamma_4C", "beta_7B", "gamma_3F", "alpha_7B", "alpha_2E", "beta_4A", "beta_6C", "beta_8E", "gamma_1B", "gamma_5F", "gamma_3B", "gamma_5D", "gamma_7F")
  reruns <- c("gamma_3B", "gamma_5D", "gamma_7F")
  proj.rows <- unlist(lapply(reversion.wells,function(x) which(sample.sheet$well==x)))
  #proj.rows <- unlist(lapply(reruns,function(x) which(sample.sheet$well==x)))
  # proj.rows <- which(sample.sheet$Investigator == "Nagel")  
  
    # (2) Here is the workhorse where we call align.identify.mutations for each sample in proj.rows
    for (i in 1:length(proj.rows)){
      align.identify.mutations(i, freq.analysis=FALSE, mut.fileout, cov.fileout)
    }
  }
```

## MUTATION ANLAYSIS

#### Read in Mutations and Analyze

This begins by reading int the mutation output file. So if analyze.isolates is set to FALSE, this is where the process begins. The output of this code block is a set of matrices and vectors that show which and how many mutations appear in which isolates and wells. In the matrices, mutations are columns and isolates or wells are rows.

```
  new.res <- read.table(file=paste(getwd(),"/Data/Mutation_output_file_(october 2013)_2b.txt", sep=""), sep="\t", header=TRUE)
  new.res.F178add <- read.table(file=paste(getwd(), "/Data/Mutation_output_file_(F182to178)_2.txt", sep=""), sep="\t", header=TRUE)  # This well gamma_6C was mislabelled as F182. This little file as the corrected rows to append on.
  new.res <- rbind(new.res, new.res.F178add)
  
  new.res.rev <- read.table(file=paste(getwd(), "/Data/Mutation_output_file(2-6-14).txt", sep=""), sep="\t", header=TRUE)
  new.res.rev <- new.res.rev[,-12]

  n.samps <- length(new.res[,1])
  anc.sites <- read.table(file=paste(getwd(),"/Data/anc_states_sites.txt", sep=""), header=TRUE)   # note it was  "F421 3863.G.A 3863" but I changed to F421 3864.A.G 3864 b/c it is the mutation in 3864 that appears 
  n.anc <- 9
  passage.samples <- which(unlist(lapply(new.res$samp.name, function(x) as.numeric(grepl("pas", x))))==1)
  not.alpha.samples <- which(unlist(lapply(new.res$samp.name, function(x) as.numeric(grepl("alpha", x))))==0)
  not.beta.samples <- which(unlist(lapply(new.res$samp.name, function(x) as.numeric(grepl("beta", x))))==0)
  not.gamma.samples <- which(unlist(lapply(new.res$samp.name, function(x) as.numeric(grepl("gamma", x))))==0)
  not.abg.samples <- intersect(not.alpha.samples, not.beta.samples)
  not.abg.samples <- intersect(not.abg.samples, not.gamma.samples)
  rem.samps <- c(passage.samples, not.abg.samples)
  p100.samps <- seq(1, n.samps)[-rem.samps]
  new.res.2 <- new.res[p100.samps, ]
  #wells.to.ommit <- c("alpha_2C", "alpha_4C", "alpha_6E", "beta_7B", "beta_7F", "gamma_2C", "gamma_4C", "gamma_3F")
  wells.to.ommit <- c("alpha_4C", "alpha_6E", "beta_7F", "gamma_4C")   # these are the wells with n=2
  
  cross.names <- rep(NA, length(new.res.2[,1]))
  cross.names[1:382] <- as.character(new.res.2$samp.name[1:382])
  new.res.names.v <- lapply(new.res.2$samp.name, function(x) unlist(strsplit(as.character(x), split="_")))
  new.res.names.v.2 <-  unlist(lapply(new.res.names.v, function(x) paste(x[1], x[2], x[3], x[length(x)], sep="_")))
  cross.names[383:length(new.res.2[,1])] <- new.res.names.v.2[383:length(new.res.names.v.2)]
  new.res.2 <- cbind(new.res.2, cross.name=cross.names)
  samp.v <- unique(new.res.2$cross.name)
  n.samps <- length(samp.v)

  n.res.rev <-length(new.res.rev[,1])
  cross.names <- as.character(new.res.rev$samp.name)
  new.res.rev <- cbind(new.res.rev, cross.name=cross.names)
  new.res.names.v <- lapply(new.res.rev$samp.name, function(x) unlist(strsplit(as.character(x), split="_")))
  new.res.names.v.2 <-  unlist(lapply(new.res.names.v, function(x) paste(x[1], x[2], x[3], x[length(x)], sep="_")))
  pas.rows <- which(lapply(new.res.names.v, function(x) x[4]) =="pas")
  if (length(pas.rows)>0){
    new.res.rev <- new.res.rev[-pas.rows,]
  }
  rem.rows <- which(new.res.rev$samp.name == "gamma_2C_F355_5-12_2")
  new.res.rev <- new.res.rev[-rem.rows,]
  
  reversion.wells <- c("beta_8A", "gamma_5B", "gamma_2C", "gamma_4E", "gamma_3F", "alpha_7B", "alpha_2E", "beta_4A", "beta_6C", "beta_8E", "gamma_1B", "gamma_5F", "gamma_3B", "gamma_5D", "gamma_7F", "beta_7B")
  rerun.wells.2 <- c("alpha_4C", "alpha_6E", "beta_8A", "gamma_5B", "beta_7F", "gamma_2C", "gamma_4E", "gamma_4C", "beta_7B", "gamma_3F", "alpha_7B", "alpha_2E", "beta_4A", "beta_6C", "beta_8E", "gamma_1B", "gamma_5F", "gamma_3B", "gamma_5D", "gamma_7F")
  
  # === Replace reversion wells in new.res.2 with those from new.res.rev
  rem.rows <- unlist(lapply(reversion.wells, function(x) which(new.res.2$Well == x)))
  new.res.2 <- new.res.2[-rem.rows,]
  add.rows <- unlist(lapply(reversion.wells, function(x) which(new.res.rev$Well==x)))
  new.res.2 <- rbind(new.res.2, new.res.rev[add.rows,])
  
  # === Remove muts < 90% of reads  (removes 4 mutations; same 4 if you use < 60% (they are all in the 50-55% zone))
  new.res.2 <- new.res.2[-which(new.res.2$p.reads < 0.9),]

  # === Remove wells.to.ommit (those with n=2) ===
  for (i in 1:length(wells.to.ommit)){
      well.id <- wells.to.ommit[i]
    rows <- which(new.res.2$Well==well.id)
    new.res.2 <- new.res.2[-rows,]
  }
  
  n.wells <- length(unique(new.res.2$Well))
    wells.v <- unique(new.res.2$Well)
    
    # === Sort wells so they cluster by backgrund
    wells.by.back <- as.data.frame(matrix(nrow=n.wells, ncol=2))
    colnames(wells.by.back) <- c("Well", "Anc.AA")
    wells.by.back$Well <- wells.v
    for (well.i in 1:n.wells){
        wells.by.back$Anc.AA[well.i] <- as.character(new.res.2$Anc.AA[which(new.res.2$Well == wells.v[well.i])[1]])
    }
    
    wells.by.back.list <- vector(mode="list", length=9)
    names(wells.by.back.list) <- anc.sites$anc.state[1:9]
    for (anc.i in 1:9){
        wells.by.back.list[[anc.i]] <- unique(as.character(new.res.2$Well[which(new.res.2$Anc.AA == as.character(anc.sites$anc.state[anc.i]))]))
    }
    
    sorted.wells <- (unlist(wells.by.back.list))
    n.wells.by.back <- unlist(lapply(wells.by.back.list, function(x) length(x)))
    breaks.by.back <- cumsum(n.wells.by.back)
    
    # === New mutation matrix, 1 row for each isolate

    unq.muts <- unique(new.res.2$unq.muts)
    sites <- as.numeric(unlist(lapply(unq.muts, function(x) unlist(strsplit(as.character(x), split="\\."))[1])))
    o.sites <- order(sites)
    unq.muts <- unq.muts[o.sites]
    sites <- sites[o.sites]
    unq.samps <- unique(new.res.2$samp.name)
    n.muts <- length(unq.muts)
    n.samps <- length(unq.samps)
    samp.v <- unique(new.res.2$cross.name)
    
    mut.matrix.n <- as.data.frame(matrix(nrow=n.samps, ncol=n.muts, data=0))
    rownames(mut.matrix.n) <- samp.v
    colnames(mut.matrix.n) <- unq.muts
    mut.matrix.nb <- mut.matrix.n
    
    # Note that mut.matrix.n has mut counts for each isolate. But I generated this by comparision to ID11 wild type. Hence the ancestral mutations were part of the set of mutations. I then went back and fixed this issue so that the comparison was against the ancestral sequence for whatever well. This means the ancestral site only appears when it undergoes a reversion. This is in in mut.matrix.nb. Note the .nb verison is what I use henceforward.
    
    
    for (samp.i in 1:n.samps){
        samp.id <- samp.v[samp.i]
        rows <- which(new.res.2$cross.name == samp.id)
        muts <- new.res.2$unq.muts[rows]
        mut.cols <- unique(unlist(lapply(muts, function(x) which(unq.muts == x))))
        mut.matrix.n[samp.i, mut.cols] <- 1
        
        back.mut.aa <- unlist(strsplit(as.character(samp.id), split="_"))[3]
        back.i <- which(anc.sites$anc.state == back.mut.aa)
        back.mut <- anc.sites$mutation[back.i]
        
        mut.rem <- which(as.character(muts) == as.character(back.mut))
        if (length(mut.rem)>0){
            muts <- muts[-mut.rem]
        }
        mut.cols <- unique(unlist(lapply(muts, function(x) which(unq.muts == x))))
        mut.matrix.nb[samp.i, mut.cols] <- 1
    }
    
    #write.csv(x=mut.matrix.nb, file="Mutation_by_isolate_matrix.csv")  # unpound to export this isolate by mutation matrix
    
    zero.muts <- which(colSums(mut.matrix.nb)==0)
    unq.muts <- unq.muts[-zero.muts]
    mut.matrix.nb <- mut.matrix.nb[,-zero.muts]
    n.muts <- length(unq.muts)
    


    counts.by.mut <- apply(mut.matrix.nb, 2, sum)
    counts.by.cat <- rep(0, max(counts.by.mut))
    names(counts.by.cat) <- seq(1, max(counts.by.mut))
    for (i in 1:max(counts.by.mut)){
        j <- which(counts.by.mut == i)
        counts.by.cat[i] <- length(j)
    }
    
    
    muts.by.well <- matrix(nrow=n.wells, ncol=n.muts, data=0)
    rownames(muts.by.well) <- wells.v
    colnames(muts.by.well) <- unq.muts
    muts.by.well.nb <- muts.by.well   # So mut.by.well.nb simply has the background mutation removed--not reveresions
    for (well.i in 1:length(wells.v)){
        well.id <- wells.v[well.i]
        rows <- which(new.res.2$Well==well.id)
        muts <- unique(new.res.2$unq.muts[rows])
        mut.cols <- unlist(lapply(muts, function(x) which(unq.muts == x)))
        muts.by.well[well.i, mut.cols] <- 1
        
        back.mut.aa <- as.character(new.res.2$Anc.AA[rows[1]])
        back.i <- which(anc.sites$anc.state == back.mut.aa)
        back.mut <- anc.sites$mutation[back.i]
        
        mut.rem <- which(as.character(muts) == as.character(back.mut))
        if (length(mut.rem)>0){
            muts <- muts[-mut.rem]
        }
        mut.cols <- unlist(lapply(muts, function(x) which(unq.muts == x)))
        muts.by.well.nb[well.i, mut.cols] <- 1
    }
    
    well.counts.by.mut <- apply(muts.by.well.nb, 2, sum)
    well.counts.by.cat <- rep(0, max(well.counts.by.mut))
    names(well.counts.by.cat) <- seq(1, max(well.counts.by.mut))
    for (i in 1:max(well.counts.by.mut)){
        j <- which(well.counts.by.mut == i)
        well.counts.by.cat[i] <- length(j)
    }
    mut.counts.by.well <- apply(muts.by.well.nb, 1, sum)
    

    #head(mut.matrix.n, 2)
    #head(mut.matrix.nb, 2)
    #head(muts.by.well,2)
    #head(muts.by.well.nb,2)
    #head(well.counts.by.mut,20)
    #head(well.counts.by.cat,20)
    #head(mut.counts.by.well,20)
```

#### Translate Mutations

This code block takes the mutations and translates them so we know what genes and residues are altered. The name of the matrix holding the info is mut.nuc.AA.m.

```
    fileout <- "Data/mut.nuc.AA.m.3.txt"
    #setwd("~/Dropbox/Work/R_programming/454_analysis/NEW 2014/Markdown")
    if (translate.mutations.bool == TRUE){
    translate.mutations(fileout)
    } else{
      mut.nuc.AA.m <- read.table(file=paste(getwd(), fileout, sep="/"), sep="\t",header=T)
    }
    head(mut.nuc.AA.m)
```

```
##   mut.DNA site wt mut gene.1 AA.site.1 from.1 to.1 anc.codon1 mut.codon1
## 1 382.A.G  382  A   G      A       108    sil  sil        AAA        AAG
## 2 449.A.G  449  A   G      A       131      T    A        ACA        GCA
## 3 454.C.T  454  C   T      A       132    sil  sil        GTC        GTT
## 4 535.C.T  535  C   T      A       159    sil  sil        CCC        CCT
## 5 976.C.T  976  C   T      A       306    sil  sil        CAC        CAT
## 6 994.C.T  994  C   T      A       312    sil  sil        CGC        CGT
##   gene.2 AA.site.2 from.2 to.2 anc.codon2 mut.codon2 all.silent in.feature
## 1   <NA>        NA   <NA> <NA>       <NA>       <NA>          1          -
## 2   <NA>        NA   <NA> <NA>       <NA>       <NA>          0          -
## 3   <NA>        NA   <NA> <NA>       <NA>       <NA>          1          -
## 4   <NA>        NA   <NA> <NA>       <NA>       <NA>          1          -
## 5     A*        93    sil  sil        CAC        CAT          1          -
## 6     A*        99    sil  sil        CGC        CGT          1          -
##   de.novo trans.v anc.usage1 mut.usage1 del.usage1 anc.usage2 mut.usage2
## 1       1     sit       1.45       0.55      -0.90         NA         NA
## 2       1     sit       0.19       1.09       0.90         NA         NA
## 3       1     sit       0.30       2.07       1.77         NA         NA
## 4       1     sit       0.05       0.59       0.54         NA         NA
## 5       1     sit       1.39       0.61      -0.78       1.39       0.61
## 6       1     sit       1.80       4.13       2.33       1.80       4.13
##   del.usage2     feat.cat
## 1         NA  A.108.NA.NA
## 2         NA  A.131.NA.NA
## 3         NA  A.132.NA.NA
## 4         NA  A.159.NA.NA
## 5      -0.78 A.306.A*. 93
## 6       2.33 A.312.A*. 99
```

## WITHIN-WELL PHYLOGENIES

The first code block here creates the first figure from the paper with three example phlogenies in it. This is followed by the code that plots the phylogeny for every well.

```
    tree.data <- read.table(file=paste(getwd(),"/Data/Trees_each_well.txt", sep=""), header=T)
    node.level <- paste(tree.data$level, tree.data$node.name, sep=".")
    node.x <- rep(NA, length(tree.data[,1]))
    node.y <- node.x
    tree.data <- cbind(tree.data, node.level, node.x, node.y)
    
  # === A 3 figure tree for the paper ===
    file.out <- "Example_tree_figure_March1_2016"
    wells.to.plot <- c("beta_1F", "alpha_8E", "alpha_4A")
    backs.for.plots <- c("F421", "J15", "J15")

  plot.example.phlogenies(wells.to.plot, backs.to.plot, file.out, file.type="pdf")
```

```
## quartz_off_screen 
##                 2
```

```
  plot.example.phlogenies(wells.to.plot, backs.to.plot, file.out, file.type="svg")
```

```
## quartz_off_screen 
##                 2
```

```
  plot.all.phylogenies(file.type="pdf", backs.to.plot=seq(1,9))
  plot.all.phylogenies(file.type="svg", backs.to.plot=3)
```

**Figure 1 from paper.** Example phylogies correspond to different types of dynamics.

**Figure from the Supplement showing all wells seeded with F355.** Note that F355 which is called here coresponds to back.to.plot=3 above. If you change that number, you are asking it to plot a different background and you’ll need to change the requested file here.

## CHARACTERIZE THE MUTATOINS

This sectionn corresponds to the section by the same name in the paper.

#### Calculate coverage.

The zone of low coverage is used in the genome plot that comes next.

```
    # ===coverage ===
    coverage.m <- read.table(file=paste(getwd(), "/Data/Coverage.by.site.output(2-6-14).txt", sep=""), header=TRUE) 
    mean.coverage <- apply(coverage.m[,5:(5577+4)], 2, mean)
    mean.coverage.by.samp <- apply(coverage.m[,5:(5577+4)], 1, mean)
    low.coverage <- which(mean.coverage < 10)
    low.cov.zone <- c(min(low.coverage), max(low.coverage))
```

#### Create The Genome (Pins in the Map) Figure

I put the actual code that creates the plot off in the *create.pins.in.map.plot* so it would be easy to create both pdf and svg versions.

```
    # === Create a "PINS IN MAP" figure ===
  
    file.name <- "pin_plot_March_2016"  
    create.pins.in.map.plot(file.name, file.type="pdf")
```

```
## quartz_off_screen 
##                 2
```

```
    create.pins.in.map.plot(file.name, file.type="svg")
```

```
## quartz_off_screen 
##                 2
```

**Figure 2 in Paper** Genome location of all mutations observed across the experiment.  
  
 #### Identify Beneficial Mutations

```
    # ==== How many of the mutations are beneficial and how many are near netural? ===
  tree.data <- read.table(file=paste(getwd(), "/Data/Trees_each_well.txt", sep=""), header=T)
    node.level <- paste(tree.data$level, tree.data$node.name, sep=".")
    node.x <- rep(NA, length(tree.data[,1]))
    node.y <- node.x
    tree.data <- cbind(tree.data, node.level, node.x, node.y)
    
    
lone.node.muts <- NULL
n.lone.node.muts <- NULL
tip.node <- NULL
n.branches <- NULL

for (well.i in 1:72){
    rows <- which(tree.data$counter == well.i)
    well.id <- tree.data$Well[rows][1]
    sub.data <- tree.data[rows,]
    tree.length <- max(sub.data$level)

    if ((well.id %in% wells.to.ommit)==FALSE){

        for (level.i in 1:tree.length){ 
            r.level <- which(sub.data$level==level.i)   
            nodes.v <- unique(sub.data$node.level[r.level])
            n.nodes <- length(nodes.v)
                            
            for (node.i in 1:n.nodes){
                node.id <- nodes.v[node.i]
                node.rows <- which(sub.data$node.level==node.id)
                node.name <- sub.data$node.name[node.rows[1]]
                
                if (length(node.rows)==1){
                    lone.node.muts <- c(lone.node.muts, as.character(sub.data$mut[node.rows]))
                    n.lone.node.muts <- c(n.lone.node.muts, as.numeric(sub.data$n.isolates[node.rows]))
                    if ((node.name %in% sub.data$bkgrd)==FALSE){
                        tip <- 1
                        n.branches <- c(n.branches, 0)
                    } else{
                        tip <- 0
                        branch.nodes <- unique(sub.data$node.name[which(as.character(sub.data$bkgrd) == as.character(node.name))])
                        n.branches <- c(n.branches, length(branch.nodes))
                    }
                    tip.node <- c(tip.node, tip)
                }
                
            }  #next node.i
        }  #next level.i
    }  # end if
} #next well.i

lone.node.data <- as.data.frame(cbind(mut= lone.node.muts, n= n.lone.node.muts, tip=as.numeric(tip.node), branches=as.numeric(n.branches)))
lone.node.data$n <- as.numeric(as.character(lone.node.data$n))
lone.node.data$branches <- as.numeric(as.character(lone.node.data$branches))
lone.node.muts <- unique(lone.node.data$mut)
tip.nodes <- which(lone.node.data$tip == 1)
n1.nodes <- which(lone.node.data$n ==1)
tip.n1.nodes <- intersect(tip.nodes, n1.nodes)
ng1.nodes <- which(lone.node.data$n >1)   # nodes with > 1 isolate sampled
ng1.branches <-which(lone.node.data$branches > 1)
ng1.node.muts <- unique(lone.node.data$mut[ng1.nodes])
ng1.branch.muts <- unique(lone.node.data$mut[ng1.branches])

lone.node.trimed <- lone.node.data[-tip.n1.nodes,]
lone.node.all <- as.character(unique(lone.node.data$mut))
lone.node.trim <- as.character(unique(lone.node.trimed$mut))

D.pro.muts <- as.character(mut.nuc.AA.m$mut.DNA[which(mut.nuc.AA.m$feat.cat == "D.pro")])
J.term.muts <-as.character(mut.nuc.AA.m$mut.DNA[which(mut.nuc.AA.m$feat.cat == "J.term")]) 
nonsyn.muts <- as.character(mut.nuc.AA.m$mut.DNA[which(mut.nuc.AA.m$all.silent == 0)])
reg.muts <- c(D.pro.muts, J.term.muts)
NSR.muts <- unique(c(nonsyn.muts, reg.muts))

lone.node.counts <- sapply(unq.muts, function(x) length(which(as.character(lone.node.muts) == as.character(x))))
names(lone.node.counts) <- unq.muts
D.pro.binary <- sapply(mut.nuc.AA.m$feat.cat, function(x) as.numeric(x=="D.pro"))
J.term.binary <- sapply(mut.nuc.AA.m$feat.cat, function(x) as.numeric(x=="J.term"))


evid.of.adapt <- as.data.frame(matrix(nrow=length(mut.nuc.AA.m[,1]), ncol=7))
colnames(evid.of.adapt) <- c("DNA.mut", "feat.cat", "N.wells", "g1.well", "ng1", "bck.2.muts", "NS.Reg")
evid.of.adapt$DNA.mut <- mut.nuc.AA.m$mut.DNA
evid.of.adapt$feat.cat <- mut.nuc.AA.m$feat.cat
evid.of.adapt$N.wells <- well.counts.by.mut
evid.of.adapt$g1.well <- as.numeric(well.counts.by.mut>1)
evid.of.adapt$ng1 <- 0
evid.of.adapt$ng1[sapply(as.character(ng1.node.muts), function(x) which(mut.nuc.AA.m$mut.DNA ==x))] <- 1
evid.of.adapt$bck.2.muts <- 0
evid.of.adapt$bck.2.muts[sapply(as.character(ng1.branch.muts), function(x) which(mut.nuc.AA.m$mut.DNA ==x))] <- 1
evid.of.adapt$NS.Reg <- 0
evid.of.adapt$NS.Reg[sapply(as.character(NSR.muts), function(x) which(mut.nuc.AA.m$mut.DNA==x))] <- 1

write.table(evid.of.adapt, file="Evidence of Adaptation Table.txt", sep="\t", col.names=T, row.names=F)

parallel <- which(evid.of.adapt$g1.well ==1)
ng1 <- which(evid.of.adapt$ng1 == 1)
g1.branch <- which(evid.of.adapt$bck.2.muts==1)
tree.based <- unique(c(ng1, g1.branch))
NSR <- which(evid.of.adapt$NS.Reg == 1)
silent <- which(evid.of.adapt$NS == 0)
parallel.int.tree <- intersect(parallel, tree.based)
parallel.not.tree <- parallel[which(sapply(parallel, function(x) x %in% tree.based)==FALSE)]
tree.not.parallel <- tree.based[which(sapply(tree.based, function(x) x %in% parallel)==FALSE)]
adaptive <- unique(c(parallel, tree.based))
NSR.not.adaptive <- NSR[which(sapply(NSR, function(x) x %in% adaptive)==FALSE)]
silent.not.adaptive <- silent[which(sapply(silent, function(x) x %in% adaptive)==FALSE)]
parallel.int.NSR <- intersect(parallel, NSR)
tree.int.NSR <- intersect(tree.based, NSR)
triple.int <- intersect(parallel.int.NSR, tree.int.NSR)

p.nsr.notree <- intersect(NSR, parallel.not.tree)
p.tree.nsr <- intersect(NSR, parallel.int.tree)
tree.nsr.nop <- intersect(NSR, tree.not.parallel)
p.sil.notree <- intersect(silent, parallel.not.tree)
p.tree.sil <- intersect(silent, parallel.int.tree)
tree.sil.nop <- intersect(silent, tree.not.parallel)
N.A <- sum(well.counts.by.mut[NSR.not.adaptive])
N.B <- sum(well.counts.by.mut[p.nsr.notree])
N.C <- sum(well.counts.by.mut[p.tree.nsr])
N.D <- sum(well.counts.by.mut[tree.nsr.nop])
N.E <- sum(well.counts.by.mut[silent.not.adaptive])
N.F <- sum(well.counts.by.mut[p.sil.notree])
N.G <- sum(well.counts.by.mut[p.tree.sil])
N.H <- sum(well.counts.by.mut[tree.sil.nop])
N1 <- sum(well.counts.by.mut[parallel])
N2 <- sum(well.counts.by.mut[tree.based])
N3 <- sum(well.counts.by.mut[NSR])
N12 <- sum(well.counts.by.mut[parallel.int.tree])
N13 <- sum(well.counts.by.mut[parallel.int.NSR])
N23 <- sum(well.counts.by.mut[tree.int.NSR])
N123 <- sum(well.counts.by.mut[triple.int])
Ntot <- sum(well.counts.by.mut)

file.name <- "Venn_Adaptive_Muts_2_split_panels"
create.venn.plot(file.name, file.type="pdf")
```

```
## quartz_off_screen 
##                 2
```

```
create.venn.plot(file.name, file.type="svg")
```

```
## quartz_off_screen 
##                 2
```

**Figure 3 in Paper** Number and evidence of adaptive mutations.

## PARALLELISM

```
    anc.order <- order(anc.sites$site[1:9])
    anc.order.names <- anc.sites$anc.state[anc.order]
  file.name <- "Well_count_dist_by_mutation_3"
  create.well.count.distribution.plot(file.name, "pdf")
```

```
## quartz_off_screen 
##                 2
```

```
  create.well.count.distribution.plot(file.name, "svg")
```

```
## quartz_off_screen 
##                 2
```

**Figure 4 from Paper** The number of occurrences of each mutation is highly uneven. A) All mutations except reversions. D-promoter mutations emphasized in red. Y-axis on left gives the well count out of 68; on right is the probability the particular mutation would occur in two wells selected at random (frequency squared). B) Reversions only. Because 4 wells were dropped from dataset, not all backgrounds had the same number of wells available for reversion (as indicated by white bars). The probability of reversion is not equal across backgrounds (\(p=0.0002\)).

```
if (calculate.parallelism.bool == TRUE){
  # === Calculate Parallelism using the function  ===

  
    parallel.list <- calculate.parallelism(muts.by.well.nb)

    pw.p.ij <- parallel.list$pw.p.ij
    pw.p.ij.dpro <- parallel.list$pw.p.ij.dpro
    pw.p.ij.nodpro <- parallel.list$pw.p.ij.nodpro
    pw.p.ij.NR <- parallel.list$pw.p.ij.NR
    pw.feats.p.ij <- parallel.list$pw.feats.p.ij
    pw.feats.p.ij.dpro <- parallel.list$pw.feats.p.ij.dpro
    pw.feats.p.ij.noD <- parallel.list$pw.feats.p.ij.noD
    dpro.by.well <- parallel.list$dpro.by.well
    
    write.table(x=pw.p.ij, file="pw_p_ij.txt", sep="\t", row.names=TRUE, col.names=TRUE)
    write.table(x=pw.feats.p.ij, file="pw_feats_p_ij.txt", sep="\t", row.names=TRUE, col.names=TRUE)
    
    reversions <- as.character(anc.sites$mutation[1:9])
    for (i in 1:9){
        x <- unlist(strsplit(reversions[i], split="\\."))
        reversions[i] <- paste(x[1], x[3], x[2], sep=".")
    }
    names(reversions) <- anc.sites$anc.state[1:9]
    
    muts.by.well.norev <- muts.by.well.nb
    cols.to.rem <- unlist(sapply(reversions, function(x) which(colnames(muts.by.well.nb) == x)))
    if (length(cols.to.rem)>0){
        muts.by.well.norev <- muts.by.well.nb[,-cols.to.rem]
    } else{
        muts.by.well.norev <- muts.by.well.nb
    }

    parallel.list.NR <- calculate.parallelism(muts.by.well.norev)

    pw.p.ij.NR2 <- parallel.list.NR$pw.p.ij
    pw.p.ij.dpro.NR2 <- parallel.list.NR$pw.p.ij.dpro
    pw.p.ij.nodpro.NR2 <- parallel.list.NR$pw.p.ij.nodpro
    pw.p.ij.NR2 <- parallel.list.NR$pw.p.ij.NR
    pw.feats.p.ij.NR2 <- parallel.list.NR$pw.feats.p.ij
    pw.feats.p.ij.dpro.NR2 <- parallel.list.NR$pw.feats.p.ij.dpro
    pw.feats.p.ij.noD.NR2 <- parallel.list.NR$pw.feats.p.ij.noD
    dpro.by.well.NR2 <- parallel.list.NR$dpro.by.well

    excl.muts <- c("3850.T.G", "2534.G.T")
    excl.mut.dex <- sapply(excl.muts, function(x) which(colnames(muts.by.well.nb)  ==x))
    muts.nb.well.F416 <- muts.by.well.nb[,-excl.mut.dex]
    parallel.list.F416 <- calculate.parallelism(muts.nb.well.F416)
    
    anc.order <- order(anc.sites$site[1:9])
    anc.order.names <- anc.sites$anc.state[anc.order]

    
    
    # ==== Summarize Parallelsism ====
    #==== Histograms of parallelism ====

    prl.within.bck <- vector("list", 9)
    prl.within.bck.nodpro <- vector("list", 9)
    prl.within.bck.feat <- vector("list", 9)
    prl.within.bck.feat.noD <- vector("list", 9)
    prl.within.bck.NR <- vector("list", 9)
    prl.within.bck.feat.NR <- vector("list", 9)
    prl.bt.bck <- vector("list", 9)
    prl.bt.bck.nodpro <- vector("list", 9)
    prl.bt.bck.feat <- vector("list", 9)
    prl.bt.bck.NR <- vector("list", 9)
    prl.within.bck.F416 <- vector("list", 9)
    sorted.wells.order <- sapply(sorted.wells, function(x) which(wells.by.back$Well == x))

    for (anc.j in 1:9){
        anc.i <- anc.order[anc.j]
        anc <- anc.sites$anc.state[anc.i]
        well.names <- as.character(wells.by.back$Well[which(wells.by.back$Anc.AA == anc)])
        wells.h <- sapply(well.names, function(w) which(sorted.wells==w))
        bt.wells <- seq(1, length(sorted.wells))
        bt.wells <- bt.wells[-wells.h]
        
        n.wells.h <- length(wells.h)
        prl.win.this.back <- matrix(nrow=0, ncol=5)
        prl.bt.this.back <- matrix(nrow=0, ncol=5)
        colnames(prl.win.this.back) <- colnames(prl.bt.this.back) <- c("Well.1", "Well.2", "Back.1", "Back.2", "Prl")
        prl.win.this.back.nodpro <- prl.win.this.back.feat <-  prl.win.this.back.feat.noD <- prl.win.this.back.NR <- prl.win.this.back.F416 <- prl.win.this.back.feat.NR <-  prl.win.this.back
        prl.bt.this.back.nodpro <- prl.bt.this.back.feat <- prl.bt.this.back
        
        for (well.i in 1:n.wells.h){
            this.well <- well.names[well.i]
            comp.wells <- wells.h[-well.i]
            prl.win.this.back <- rbind(prl.win.this.back, as.data.frame(cbind(rep(this.well, n.wells.h-1), sorted.wells[comp.wells], rep(as.character(anc), n.wells.h-1), rep(as.character(anc), n.wells.h-1), pw.p.ij[comp.wells,wells.h[well.i]])))
            prl.win.this.back.nodpro <- rbind(prl.win.this.back.nodpro, as.data.frame(cbind(rep(this.well, n.wells.h-1), sorted.wells[comp.wells], rep(as.character(anc), n.wells.h-1), rep(as.character(anc), n.wells.h-1), pw.p.ij.nodpro[comp.wells,wells.h[well.i]])))
            prl.win.this.back.NR <- rbind(prl.win.this.back.NR, as.data.frame(cbind(rep(this.well, n.wells.h-1), sorted.wells[comp.wells], rep(as.character(anc), n.wells.h-1), rep(as.character(anc), n.wells.h-1), pw.p.ij.NR[comp.wells,wells.h[well.i]])))
            prl.win.this.back.feat <- rbind(prl.win.this.back.feat, as.data.frame(cbind(rep(this.well, n.wells.h-1), sorted.wells[comp.wells], rep(as.character(anc), n.wells.h-1), rep(as.character(anc), n.wells.h-1), pw.feats.p.ij[comp.wells,wells.h[well.i]])))
            prl.win.this.back.feat.noD <- rbind(prl.win.this.back.feat.noD, as.data.frame(cbind(rep(this.well, n.wells.h-1), sorted.wells[comp.wells], rep(as.character(anc), n.wells.h-1), rep(as.character(anc), n.wells.h-1), pw.feats.p.ij.noD[comp.wells,wells.h[well.i]])))
            
            prl.win.this.back.feat.NR <- rbind(prl.win.this.back.feat.NR, as.data.frame(cbind(rep(this.well, n.wells.h-1), sorted.wells[comp.wells], rep(as.character(anc), n.wells.h-1), rep(as.character(anc), n.wells.h-1), pw.feats.p.ij.NR2[comp.wells,wells.h[well.i]])))
            
            prl.bt.this.back <- rbind(prl.bt.this.back, as.data.frame(cbind(rep(this.well, length(bt.wells)), sorted.wells[bt.wells], rep(as.character(anc), length(bt.wells)), wells.by.back$Anc.AA[sorted.wells.order[bt.wells]], pw.p.ij[bt.wells,wells.h[well.i]])))
            prl.bt.this.back.nodpro <- rbind(prl.bt.this.back.nodpro, as.data.frame(cbind(rep(this.well, length(bt.wells)), sorted.wells[bt.wells], rep(as.character(anc), length(bt.wells)), wells.by.back$Anc.AA[sorted.wells.order[bt.wells]], pw.p.ij.nodpro[bt.wells,wells.h[well.i]])))
            prl.bt.this.back.feat <- rbind(prl.bt.this.back.feat, as.data.frame(cbind(rep(this.well, length(bt.wells)), sorted.wells[bt.wells], rep(as.character(anc), length(bt.wells)), wells.by.back$Anc.AA[sorted.wells.order[bt.wells]], pw.feats.p.ij[bt.wells,wells.h[well.i]])))
        } #next well.i
        
        colnames(prl.win.this.back) <- colnames(prl.win.this.back.nodpro) <- colnames(prl.win.this.back.feat) <- colnames(prl.win.this.back.NR) <- colnames(prl.win.this.back.feat.NR) <-  c("Well.1", "Well.2", "Back.1", "Back.2", "Prl")
        colnames(prl.bt.this.back) <- colnames(prl.bt.this.back.nodpro) <- colnames(prl.bt.this.back.feat) <- c("Well.1", "Well.2", "Back.1", "Back.2", "Prl")
    
        remove.duplicates <- function(prl.mat){
            wells.12 <- apply(prl.mat, 1, function(x) paste(sort(x[1:2]), collapse="_"))
            u.wells12 <- unique(wells.12)
            u.dex <- sapply(u.wells12, function(x) which(wells.12 == x)[1])
            prl.mat.trim <- prl.mat[u.dex, ]
            return(prl.mat.trim)
        }   
        
        prl.win.this.back <- remove.duplicates(prl.win.this.back)
        prl.win.this.back.nodpro <- remove.duplicates(prl.win.this.back.nodpro)
        prl.win.this.back.feat <- remove.duplicates(prl.win.this.back.feat)
        prl.win.this.back.feat.noD <- remove.duplicates(prl.win.this.back.feat.noD)
        prl.win.this.back.NR <- remove.duplicates(prl.win.this.back.NR)
        prl.win.this.back.feat.NR <- remove.duplicates(prl.win.this.back.feat.NR)
        prl.bt.this.back <- remove.duplicates(prl.bt.this.back)
        prl.bt.this.back.nodpro <- remove.duplicates(prl.bt.this.back.nodpro)
        prl.bt.this.back.feat <- remove.duplicates(prl.bt.this.back.feat)
        
        prl.within.bck[[anc.j]] <- prl.win.this.back
        prl.within.bck.nodpro[[anc.j]] <- prl.win.this.back.nodpro
        prl.within.bck.feat[[anc.j]] <- prl.win.this.back.feat
        prl.within.bck.feat.noD[[anc.j]] <- prl.win.this.back.feat.noD
        prl.within.bck.NR[[anc.j]] <- prl.win.this.back.NR
        prl.within.bck.feat.NR[[anc.j]] <- prl.win.this.back.feat.NR
        prl.bt.bck[[anc.j]] <- prl.bt.this.back
        prl.bt.bck.nodpro[[anc.j]] <- prl.bt.this.back.nodpro
        prl.bt.bck.feat[[anc.j]] <- prl.bt.this.back.feat
        if (anc.j == 1){
            prl.all.bt.bck <- prl.bt.bck[[anc.j]]
            prl.all.bt.bck.nodpro <- prl.bt.bck.nodpro[[anc.j]]
            prl.all.bt.bck.feat <- prl.bt.bck.feat[[anc.j]]
        } else{
            prl.all.bt.bck <- rbind(prl.all.bt.bck, prl.bt.bck[[anc.j]])
            prl.all.bt.bck.nodpro <- rbind(prl.all.bt.bck.nodpro, prl.bt.bck.nodpro[[anc.j]])
            prl.all.bt.bck.feat <- rbind(prl.all.bt.bck.feat, prl.bt.bck.feat[[anc.j]])
        }
    }  # next anc.j
    
    prl.all.bt.bck <- as.data.frame(prl.all.bt.bck)
    prl.all.bt.bck.nodpro <- as.data.frame(prl.all.bt.bck.nodpro)
    prl.all.bt.bck.feat <- as.data.frame(prl.all.bt.bck.feat)
    prl.all.bt.bck$Prl <- as.numeric(prl.all.bt.bck$Prl)
    prl.all.bt.bck.nodpro$Prl <- as.numeric(prl.all.bt.bck.nodpro$Prl)
    prl.all.bt.bck.feat$Prl <- as.numeric(prl.all.bt.bck.feat$Prl)  
    
    prl.mean.nodpro <- rep(NA, 9)
    prl.mean.DNA <- rep(NA, 9)
    prl.mean.feat <- rep(NA, 9)
    prl.mean.feat.noD <- rep(NA, 9)
    prl.mean.NR <- rep(NA, 9)
    prl.mean.feat.NR <- rep(NA, 9)
    for (anc.i in 1:9){
        prl.mean.nodpro[anc.i] <- mean(as.numeric(prl.within.bck.nodpro[[anc.i]][,5]), na.rm=TRUE)
        prl.mean.DNA[anc.i] <- mean(as.numeric(prl.within.bck[[anc.i]][,5]), na.rm=TRUE)
        prl.mean.feat[anc.i] <- mean(as.numeric(prl.within.bck.feat[[anc.i]][,5]), na.rm=TRUE)
        prl.mean.feat.noD[anc.i] <- mean(as.numeric(prl.within.bck.feat.noD[[anc.i]][,5]), na.rm=TRUE)
        prl.mean.NR[anc.i] <- mean(as.numeric(prl.within.bck.NR[[anc.i]][,5]), na.rm=TRUE)
        prl.mean.feat.NR[anc.i] <- mean(as.numeric(prl.within.bck.feat.NR[[anc.i]][,5]), na.rm=TRUE)
    }
    names(prl.mean.nodpro) <- names(prl.mean.DNA) <- names(prl.mean.feat) <- names(prl.mean.feat.noD) <- names(prl.mean.NR) <- names(prl.mean.feat.NR) <- anc.order.names

    prl.all.bt.bck.mean <- mean(as.numeric(prl.all.bt.bck[,5]))
    prl.all.win.back <- prl.within.bck[[1]]
    for (anc.i in 2:9){
        prl.all.win.back <- rbind(prl.all.win.back, prl.within.bck[[anc.i]])
    }
    prl.all.win.bck.mean <- mean(as.numeric(prl.all.win.back[,5]))
}
```

#### Create Parallelism Within Background Plot

```
    pw.p.ij <- read.table(file="pw_p_ij.txt", header=TRUE)
    pw.feats.p.ij <- read.table(file="pw_feats_p_ij.txt", header=TRUE)
    comp.m.nuc <- summarize.variation.in.parallelism(pw.p.ij)$comp.m   # Provides P.ij for all within bacvkground comparisons
        comp.feats.m.nuc <- summarize.variation.in.parallelism(pw.feats.p.ij)$comp.m   # Provides P.ij for all within bacvkground comparisons
        
        file.name <- "Parallelism_between_Wells_2"
        create.within.back.parallelism.plot(file.name, file.type="pdf")
```

```
## quartz_off_screen 
##                 2
```

```
        create.within.back.parallelism.plot(file.name, file.type="svg")
```

```
## quartz_off_screen 
##                 2
```

**Figure 5 in Paper. Amount of parallelism between pairs of wells within backgrounds.** Parallelism is defined as the mean of the proportion of mutations in one well shared with the other and the reciprocal comparison. Note that individual pairwise comparisions (x symbols) with same value are stacked vertically, creating the bars. A) Mutations are shared only when they match at the nucleotide level. B) Mutations match when they occur at the same codon or in the same regulatory element. Backgrounds are indicated on the y-axis; only within-background comparisons are made. Background F416 has elevated parallelism in large part because it experienced much higher reversion. Once reversions are removed, a randomization test showed no evidence for differences in parallelism between backgrounds at the nucleotide level (\(p\approx 0.58\)) nor the regulatory/codon level (\(p \approx 0.56\)).  
  
 #### Create Parallelism Between All Wells Heatmap

```
        comps.nuc <- summarize.variation.in.parallelism(pw.p.ij.NR)
      comps.feats <- summarize.variation.in.parallelism(pw.feats.p.ij.NR2)
      file.name <- "across_back_parallelism_match"
      create.between.all.wells.heatmap(file.name, file.type="pdf")
```

```
## quartz_off_screen 
##                 2
```

```
      create.between.all.wells.heatmap(file.name, file.type="svg")
```

```
## quartz_off_screen 
##                 2
```

**Figure 6 in Paper. Parallelism within backgrounds is only slightly higher than parallelism between backgrounds.**(A) Parallelism between all pairs of wells from low (light) to high (dark). Above the diagonal in red is parallelism at nucleotide level; below the diagonal in blue is parallelism at the regulatory/codon level. Squares along diagonal are within background comparisons, all others are between backgrounds. Notice that within-background quadrants are not noticeably darker than between-background quadrants. (B-C) Histograms comparing within- vs. between-background parallelism pooled across backgrounds at the nucleotide (B) and regulatory/codon (C) level. The histograms reveal that parallelism is, on average, slightly higher within backgrounds (\(p<0.01\)).

#### Calcualte parallelism within and between and their ratio

```
    # Note that R is the amount of variation attributable to background
    # P is the meawn parallelims.
    # mWIN.mBT is the ratio of mean within P to mean between P
      
    sum.var.real <- summarize.variation.in.parallelism(pw.p.ij)
    R.real <- sum.var.real$R
    P.mean.real <- sum.var.real$mean.P
    R2.WIN <- sum.var.real$R2.all
    mWIN.mBT.real <- sum.var.real$mWIN.mBT
    
    sum.var.real.feats <- summarize.variation.in.parallelism(pw.feats.p.ij)
    R.real.feats <- sum.var.real.feats$R
    mWIN.mBT.real.feats <- sum.var.real.feats$mWIN.mBT
    P.mean.real.feats <- sum.var.real.feats$mean.P
    R2.WIN.feats <- sum.var.real.feats$R2.all

    sum.var.real.NR <- summarize.variation.in.parallelism(pw.p.ij.NR)
    R.real.NR <- sum.var.real.NR$R
    P.mean.real.NR <- sum.var.real.NR$mean.P
    mWIN.mBT.real.NR <- sum.var.real.NR$mWIN.mBT
    R2.WIN.NR <- sum.var.real.NR$R2.all
    
    sum.var.real.feats.NR <-  summarize.variation.in.parallelism(pw.feats.p.ij.NR2)
    R.real.feats.NR <- sum.var.real.feats.NR$R
    P.mean.real.feats.NR <- sum.var.real.feats.NR$mean.P
    mWIN.mBT.real.feats.NR <- sum.var.real.feats.NR$mWIN.mBT
    R2.WIN.feats.NR <- sum.var.real.feats.NR$R2.all
    
    comp.m.nuc <- summarize.variation.in.parallelism(pw.p.ij)$comp.m
    comp.btb.nuc <- summarize.variation.in.parallelism(pw.p.ij)$comp.btb
```

This code block calculates the within and between levels of parallelism. Within backgrounds, the average level of parallelims is, 0.32, 0.25, 0.24, 0.32, 0.32, 0.21, 0.27, 0.46, 0.15 for backgrounds J15, J20, F5, F178, F182, F322, F355, F416, F421 respectively. The overall mean parallelism within is 0.282. The mean level of parallelism for comparisions between backgrounds is 0.219. The ratio of within to between is 1.294.

At the feature level, the mean within parallelism is 0.41, 0.47, 0.38, 0.39, 0.35, 0.28, 0.34, 0.52, 0.26 for backgrounds J15, J20, F5, F178, F182, F322, F355, F416, F421 respectively. Overall mean within is 0.377. The mean level of parallelism for comparisions between backgrounds is 0.313. The ratio of within to between is 1.208.

In the next code block we use bootstrap randomization and find that the difference between backgrounds are not significant, but that parallelism within is significantly greater than parallelism between.

#### Assess significance using bootstrap randomization

```
    n.boots <- 100
    if (bootstap.win.bet.parallelism.bool == TRUE){
    
    R.by.boot <- R.feat.by.boot <- R2.WIN.by.boot <- R2.WIN.feat.by.boot <- rep(NA, n.boots)
    P.mean.by.boot <- P.feat.mean.by.boot <- as.data.frame(matrix(nrow=n.boots, ncol=9))
    colnames(P.mean.by.boot) <- names(P.mean.real)
    mWIN.mBT.boot <- rep(NA,n.boots)
    mWIN.mBT.feats.boot <- rep(NA,n.boots)
    
        # --- identify reversions ---
        reversion.v <- rep(NA, 9)
        reversion2.v <- rep(NA, 9)
        names(reversion.v) <- names(reversion2.v) <- anc.sites$anc.state[anc.order]
        for (anc.i in 1:9){
            anc <- as.character(anc.sites$mutation[anc.order[anc.i]])
            anc.split <- unlist(strsplit(anc, split = "\\."))
            rev <- paste(anc.split[1], anc.split[3], anc.split[2], sep=".")
            col.id <- which(colnames(muts.by.well.nb) == rev)
            if (length(col.id)>0){
                reversion.v[anc.i] <- col.id
                reversion2.v[anc.i] <- rev
            }
        }
    
      for (boot.i in 1:100){
    
        print(paste("boot.i=", boot.i))
        muts.by.well.boot <- randomize.muts.by.well.matrix(muts.by.well.nb, reversion.v, reversion2.v)  
        parallel.boot <- calculate.parallelism(muts.by.well.boot)
  
        boot.pw.p.ij <- parallel.boot$pw.p.ij
        boot.pw.p.ij.dpro <- parallel.boot$pw.p.ij.dpro
        boot.pw.feats.p.ij <- parallel.boot$pw.feats.p.ij
        boot.pw.feats.p.ij.dpro <- parallel.boot$pw.feats.p.ij.dpro
        boot.pw.p.ij.NR <- parallel.boot$pw.p.ij.NR
        boot.dpro.by.well <- parallel.boot$dpro.by.well
    
        Nuc.lv.res <- summarize.variation.in.parallelism(boot.pw.p.ij)
        Feat.lv.res <- summarize.variation.in.parallelism(boot.pw.feats.p.ij)
        R.by.boot[boot.i] <- Nuc.lv.res$R
        R.feat.by.boot[boot.i] <- Feat.lv.res$R
        R2.WIN.by.boot[boot.i] <- Nuc.lv.res$R2.all
        R2.WIN.feat.by.boot[boot.i] <- Feat.lv.res$R2.all
        P.mean.by.boot[boot.i,] <- Nuc.lv.res$mean.P
        P.feat.mean.by.boot[boot.i,] <- Feat.lv.res$mean.P
        mWIN.mBT.boot[boot.i] <- Nuc.lv.res$mWIN.mBT
        mWIN.mBT.feats.boot[boot.i] <- Feat.lv.res$mWIN.mBT
      }
        
        write.table(P.mean.by.boot, file=paste(getwd(),"/Parallelism_bootstrap/P.mean.by.boot.txt", sep=""), sep="\t")
        write.table(P.feat.mean.by.boot,  file=paste(getwd(),"/Parallelism_bootstrap/P.feat.mean.by.boot.txt", sep=""), sep="\t")
        write.table(R.by.boot, file=paste(getwd(),"/Parallelism_bootstrap/R.by.boot.txt", sep=""), sep="\t")
        write.table(R.feat.by.boot, file=paste(getwd(),"/Parallelism_bootstrap/R.feat.by.boot.txt", sep=""), sep="\t")
        write.table(mWIN.mBT.boot, file=paste(getwd(),"/Parallelism_bootstrap/mWIN.mBT.boot.txt", sep=""), sep="\t")
        write.table(mWIN.mBT.feats.boot, file=paste(getwd(),"/Parallelism_bootstrap/mWIN.mBT.feats.boot.txt", sep=""), sep="\t")
        write.table(R2.WIN.by.boot, file=paste(getwd(),"/Parallelism_bootstrap/R2.WIN.boot.txt", sep=""), sep="\t")
        write.table(R2.WIN.feat.by.boot, file=paste(getwd(),"/Parallelism_bootstrap/R2.WIN.feat.boot.txt", sep=""), sep="\t")
    } else{
      P.mean.by.boot <- read.table(file=paste(getwd(),"/Parallelism_bootstrap/P.mean.by.boot.txt", sep=""))
      P.feat.mean.by.boot <- read.table(file=paste(getwd(),"/Parallelism_bootstrap/P.feat.mean.by.boot.txt", sep=""))
      R.by.boot <- read.table(file=paste(getwd(),"/Parallelism_bootstrap/R.by.boot.txt", sep=""))[,1]
      R.feat.by.boot <- read.table(file=paste(getwd(),"/Parallelism_bootstrap/R.feat.by.boot.txt", sep=""))[,1]
      mWIN.mBT.boot <- read.table(file=paste(getwd(),"/Parallelism_bootstrap/mWIN.mBT.boot.txt", sep=""))[,1]
      mWIN.mBT.feats.boot <- read.table(file=paste(getwd(),"/Parallelism_bootstrap/mWIN.mBT.feats.boot.txt", sep=""))[,1]
      R2.WIN.by.boot <- read.table(file=paste(getwd(),"/Parallelism_bootstrap/R2.WIN.boot.txt", sep=""))[,1]
      R2.WIN.feat.by.boot <- read.table(file=paste(getwd(),"/Parallelism_bootstrap/R2.WIN.feat.boot.txt", sep=""))[,1]
    }
    

    P.mean.boot.SS <- apply(P.mean.by.boot, 1, function(x) sum((x-mean(x))^2))
    P.mean.real.SS <- sum((P.mean.real.NR-mean(P.mean.real.NR))^2)
    P.mean.boot.max <- apply(P.mean.by.boot, 1, max)
    P.mean.real.max <- max(P.mean.real)
    P.mean.boot.min <- apply(P.mean.by.boot, 1, min)
    P.mean.real.min <- min(P.mean.real)
    
    
    P.mean.boot.feats.SS <- apply(P.feat.mean.by.boot, 1, function(x) sum((x-mean(x))^2))
    P.mean.real.feats.SS <- sum((P.mean.real.feats-mean(P.mean.real.feats))^2)
    P.mean.real.feats.SS.NR <- sum((P.mean.real.feats.NR-mean(P.mean.real.feats.NR))^2)
    P.mean.feats.boot.max <- apply(P.feat.mean.by.boot, 1, max)
    P.mean.feat.real.max <- max(P.mean.real.feats)

    p.val.R <- length(which(R.by.boot >= R.real.NR))/n.boots                    # Tells us no evidence for difference among backgrounds.
    p.val.mean.SS <- length(which(P.mean.boot.SS >= P.mean.real.SS))/n.boots    
    p.val.mean.max <- length(which(P.mean.boot.max >= P.mean.real.max))/n.boots
    p.val.mean.min <- length(which(P.mean.boot.min >= P.mean.real.min))/n.boots
    
    p.val.R2.WIN <- length(which(R2.WIN.by.boot >= R2.WIN))/n.boots           # This says that within background parallism is different from between
  p.val.R2.WIN.feat <- length(which(R2.WIN.feat.by.boot >= R2.WIN.feats))/n.boots
    
    p.val.R.feats <- length(which(R.feat.by.boot >= R.real.feats.NR))/n.boots   # All this holds at the feature level
    p.val.mean.feats.SS <- length(which(P.mean.boot.feats.SS >= P.mean.real.feats.SS.NR))/n.boots
    p.val.mean.feats.max <- length(which(P.mean.feats.boot.max >= P.mean.feat.real.max))/n.boots
    
    p.val.mWIN.mBT <- length(which(mWIN.mBT.boot >= mWIN.mBT.real.NR))/n.boots      #The ratio is significant just like R2.WIN is. 
    p.val.mWIN.mBT.feat <- length(which(mWIN.mBT.feats.boot >= mWIN.mBT.real.feats))
```

#### Deteremine if Reversion Differs by Background

```
    # === Now compress further by asking how many wells was each mutation seen in for each background ===
        anc.states.v <- anc.sites$anc.state[1:9]
        n.backs <- length(anc.sites[,1])-1
        mut.mat.by.back.real <- mut.mat.by.back <- get.mut.mat.by.back(muts.by.well.nb)
        n.wells.by.back <- rep(NA, 9)
        names(n.wells.by.back) <- anc.order.names
        n.wells.by.back <- unlist(lapply(names(n.wells.by.back), function(x) length(which(wells.by.back$Anc.AA == x))))
        names(n.wells.by.back) <- anc.order.names
    
        
        # --- identify reversions ---
        reversion.v <- rep(NA, 9)
        reversion2.v <- rep(NA, 9)
        names(reversion.v) <- names(reversion2.v) <- anc.sites$anc.state[anc.order]
        for (anc.i in 1:9){
            anc <- as.character(anc.sites$mutation[anc.order[anc.i]])
            anc.split <- unlist(strsplit(anc, split = "\\."))
            rev <- paste(anc.split[1], anc.split[3], anc.split[2], sep=".")
            col.id <- which(colnames(muts.by.well.nb) == rev)
            if (length(col.id)>0){
                reversion.v[anc.i] <- col.id
                reversion2.v[anc.i] <- rev
            }
        }
        
        # === Test for whether reversions differ significantly b/t backgrounds ===
        n.reversions <- rep(NA, 9)
        names(n.reversions) <- anc.order.names
        for (anc.i in 1:9){
            if (is.na(reversion.v[anc.i])){
                n.reversions[anc.i] <- 0
            } else{
            n.reversions[anc.i] <- mut.mat.by.back.real[anc.i, reversion.v[anc.i]]
            }
        }
        p.null <- rep(sum(n.reversions)/n.wells, 9)
        p.alt <- n.reversions/n.wells.by.back
        like.null.v <- dmultinom(x=n.reversions, prob=p.null, log=TRUE)
        like.alt.v <- dmultinom(x=n.reversions, prob=p.alt, log=TRUE)
        LR.rev.real <- like.alt.v - like.null.v
        
        n.boots <- 10000
        LR.rev.boot <- rep(NA, n.boots)
        n.revs <- sum(n.reversions)
        n.non.revs <- n.wells-n.revs
        back.dex.v <- rep(NA, n.wells)
        i <- 1
        for (anc.i in 1:9){
            back.dex.v[i:(i+n.wells.by.back[anc.i])] <- anc.i
            i <- i+n.wells.by.back[anc.i]
        }
        back.dex.v <- back.dex.v[1:n.wells]
        back.dex.list <- lapply(seq(1,9), function(x) which(back.dex.v==x))
        
        for (boot.i in 1:n.boots){
            rv <- c(rep(1, n.revs), rep(0, n.non.revs))
            rv <- rv[order(runif(n=n.wells))]
            rv.n <- unlist(lapply(back.dex.list, function(x) sum(rv[x])))
            p.null <- rep(sum(n.reversions)/n.wells, 9)
            p.alt <- rv.n/n.wells.by.back
            like.null.v <- dmultinom(x=rv.n, prob=p.null, log=TRUE)
            like.alt.v <- dmultinom(x=rv.n, prob=p.alt, log=TRUE)
            LR.rev.boot[boot.i] <- like.alt.v - like.null.v
        }
        
        p.val.reversion <- length(which(LR.rev.boot > LR.rev.real))/n.boots
```

This results corresponds to Figure 4B in table, where the p-value associated with the null is small (0.0014).

## EPISTASIS

#### Likelihood Ratio Test for Effect of Background on Each Mutation

```
            # --- Full data set --- Showing evidence of background dependnce 
        mut.mat.by.back.real <- get.mut.mat.by.back(muts.by.well.nb)
        LR.results <- calc.like.ratio(mut.mat.by.back.real, anc.to.excl=NULL, muts.to.excl=NULL)
        LR.results.bin.real <- calc.like.ratio.binomial.based(mut.mat.by.back.real, anc.to.excl=NULL, muts.to.excl=NULL)
        LR.bin.real.bymut <- LR.results.bin.real$alt.null.like.diff
        bymut.like.diff.bin <- LR.results.bin.real$bymut.like.diff
        LR.bin.top.10 <- LR.results.bin.real$top.10.difs
        sum.muts.real <- LR.results$sum.of.muts
        LR.real.byanc <- LR.results$byanc.like.ratio
        LR.real.bymut <- LR.results$bymut.like.ratio
        bymut.like.diffs <- LR.results$bymut.like.diff
    
        n.boots <- 100
        LR.boot.dist.byanc <- rep(NA, n.boots)
        LR.boot.dist.bymut <- rep(NA, n.boots)
        LR.boot.top10 <- matrix(nrow=n.boots, ncol=10)
        #LR.bin.boot.dist.byanc <- rep(NA, n.boots)
        LR.bin.boot.dist.bymut <- rep(NA, n.boots)
        LR.bin.boot.top10 <- matrix(nrow=n.boots, ncol=10)
        boot.i <- 1
        
        while (boot.i <= n.boots){
            muts.by.well.boot <- randomize.muts.by.well.matrix(muts.by.well.nb, reversion.v, reversion2.v, anc.to.excl=NULL, muts.to.excl=NULL)
            sum.muts.boot <- sum(muts.by.well.boot)
            if (sum.muts.boot == sum.muts.real){
                #print (boot.i)
                mut.mat.by.back.boot <- get.mut.mat.by.back(muts.by.well.boot)
                LR.boot.res <- calc.like.ratio(mut.mat.by.back.boot, anc.to.excl=NULL, muts.to.excl=NULL)
                LR.boot.dist.byanc[boot.i] <- LR.boot.res$byanc.like.ratio
                LR.boot.dist.bymut[boot.i] <- LR.boot.res$bymut.like.ratio
                LR.boot.top10[boot.i,] <- LR.boot.res$top.10.difs$difs
                LR.bin.boot.res <- calc.like.ratio.binomial.based(mut.mat.by.back.boot, anc.to.excl=NULL, muts.to.excl=NULL)
                LR.bin.boot.dist.bymut[boot.i] <- LR.bin.boot.res$alt.null.like.diff
                LR.bin.boot.top10[boot.i,] <- LR.bin.boot.res$top.10.difs$difs
                boot.i <- boot.i + 1
            }  # if (sum.muts.boot == sum.muts.real){
        }  # for (boot.i in ...)
        
        LR.bin.boot.dist.bymut.FULL <- LR.bin.boot.dist.bymut
        top.1.v <- as.numeric(apply(LR.boot.top10, 1, function(x) max(x)))
        #p.val.byanc.FULL <- length(which(LR.boot.dist.byanc > LR.real.byanc))/n.boots
        p.val.bymut.FULL <- length(which(LR.boot.dist.bymut > LR.real.bymut))/n.boots
        hist(LR.boot.dist.bymut, xlim=c(min(LR.boot.dist.bymut), 1.1*max(c(LR.boot.dist.bymut, LR.real.bymut))), xlab=expression(Lambda), main=expression(paste("Distribtion of ", Lambda, " under Null and Observed ", Lambda)), col="grey", breaks=10)
        mtext(side=3, line=0, text="All Mutations", font=2)
        points(LR.real.bymut,0, pch=23, bg="red")
        legend("topright", pch=23, pt.bg="red", legend="Observed")
```

```
        #p.val.bin.bymut.FULL <- length(which(LR.bin.boot.dist.bymut> LR.bin.real.bymut))/n.boots
```

The result of the full LR Test is highly significant.

#### Identify which mutations are driving significance

Now we remove the mutation with the smallest liklihood and rerun the test. Continue removing until we do not get a significant result.

```
        # --- Remove a mutation (e..g 2534.G.T) from the dataset and rerun ---
        mut.excl <- "2534.G.T"
        mut.mat.by.back.real <- get.mut.mat.by.back(muts.by.well.nb)
        LR.results <- calc.like.ratio(mut.mat.by.back.real, anc.to.excl=NULL, muts.to.excl=mut.excl)
        LR.results.bin <- calc.like.ratio.binomial.based(mut.mat.by.back.real, anc.to.excl=NULL, muts.to.excl=mut.excl)
        LR.bin.real.bymut <- LR.results.bin$alt.null.like.diff
        sum.muts.real <- LR.results$sum.of.muts
        LR.real.byanc <- LR.results$byanc.like.ratio
        LR.real.bymut <- LR.results$bymut.like.ratio
        bymut.like.diffs <- LR.results$bymut.like.diff
    
        n.boots <- 100
        LR.boot.dist.byanc <- rep(NA, n.boots)
        LR.boot.dist.bymut <- rep(NA, n.boots)
        LR.boot.top10 <- matrix(nrow=n.boots, ncol=10)
        #LR.bin.boot.dist.byanc <- rep(NA, n.boots)
        LR.bin.boot.dist.bymut <- rep(NA, n.boots)
        LR.bin.boot.top10 <- matrix(nrow=n.boots, ncol=10)
        boot.i <- 1
        
        while (boot.i <= n.boots){
            muts.by.well.boot <- randomize.muts.by.well.matrix(muts.by.well.nb, reversion.v, reversion2.v, anc.to.excl=NULL, muts.to.excl=mut.excl)
            sum.muts.boot <- sum(muts.by.well.boot)
            if (sum.muts.boot == sum.muts.real){
                #print (boot.i)
                mut.mat.by.back.boot <- get.mut.mat.by.back(muts.by.well.boot)
                LR.boot.res <- calc.like.ratio(mut.mat.by.back.boot, anc.to.excl=NULL, muts.to.excl=NULL)
                LR.boot.dist.byanc[boot.i] <- LR.boot.res$byanc.like.ratio
                LR.boot.dist.bymut[boot.i] <- LR.boot.res$bymut.like.ratio
                LR.boot.top10[boot.i,] <- LR.boot.res$top.10.difs$difs
                LR.bin.boot.res <- calc.like.ratio.binomial.based(mut.mat.by.back.boot, anc.to.excl=NULL, muts.to.excl=NULL)
                LR.bin.boot.dist.bymut[boot.i] <- LR.bin.boot.res$alt.null.like.diff
                LR.bin.boot.top10[boot.i,] <- LR.bin.boot.res$top.10.difs$difs
                boot.i <- boot.i + 1
            }  # if (sum.muts.boot == sum.muts.real){
        }  # for (boot.i in ...)
        
        
        top.1.v.ex <- as.numeric(apply(LR.boot.top10, 1, function(x) max(x)))
        p.val.byanc.ex <- length(which(LR.boot.dist.byanc > LR.real.byanc))/n.boots
        p.val.bymut.ex <- length(which(LR.boot.dist.bymut > LR.real.bymut))/n.boots
        p.val.bin.bymut.ex <- length(which(LR.bin.boot.dist.bymut> LR.bin.real.bymut))/n.boots
        p.val.bymut.FULL <- length(which(LR.boot.dist.bymut > LR.real.bymut))/n.boots
    
        hist(LR.boot.dist.bymut, xlim=c(min(LR.boot.dist.bymut), 1.1*max(c(LR.boot.dist.bymut, LR.real.bymut))), xlab=expression(Lambda), main=expression(paste("Distribtion of ", Lambda, " under Null and Observed ", Lambda)), col="grey", breaks=10)
        mtext(side=3, line=0, text="Without 2534.G.T", font=2)
        points(LR.real.bymut,0, pch=23, bg="red")
        legend("topright", pch=23, pt.bg="red", legend="Observed")
```

```
        # --- Remove a mutation (e..g 2534.G.T and "2397.A.G") from the dataset and rerun ---
        mut.excl <- c("2534.G.T", "2397.A.G")
        # p.value = 0.001
        mut.mat.by.back.real <- get.mut.mat.by.back(muts.by.well.nb)
        LR.results <- calc.like.ratio(mut.mat.by.back.real, anc.to.excl=NULL, muts.to.excl=mut.excl)
        LR.results.bin <- calc.like.ratio.binomial.based(mut.mat.by.back.real, anc.to.excl=NULL, muts.to.excl=mut.excl)
        LR.bin.real.bymut <- LR.results.bin$alt.null.like.diff
        sum.muts.real <- LR.results$sum.of.muts
        LR.real.byanc <- LR.results$byanc.like.ratio
        LR.real.bymut <- LR.results$bymut.like.ratio
        bymut.like.diffs <- LR.results$bymut.like.diff
    
        n.boots <- 100
        LR.boot.dist.byanc <- rep(NA, n.boots)
        LR.boot.dist.bymut <- rep(NA, n.boots)
        LR.boot.top10 <- matrix(nrow=n.boots, ncol=10)
        #LR.bin.boot.dist.byanc <- rep(NA, n.boots)
        LR.bin.boot.dist.bymut <- rep(NA, n.boots)
        LR.bin.boot.top10 <- matrix(nrow=n.boots, ncol=10)
        boot.i <- 1
        
        while (boot.i <= n.boots){
            muts.by.well.boot <- randomize.muts.by.well.matrix(muts.by.well.nb, reversion.v, reversion2.v, anc.to.excl=NULL, muts.to.excl=mut.excl)
            sum.muts.boot <- sum(muts.by.well.boot)
            if (sum.muts.boot == sum.muts.real){
                #print (boot.i)
                mut.mat.by.back.boot <- get.mut.mat.by.back(muts.by.well.boot)
                LR.boot.res <- calc.like.ratio(mut.mat.by.back.boot, anc.to.excl=NULL, muts.to.excl=NULL)
                LR.boot.dist.byanc[boot.i] <- LR.boot.res$byanc.like.ratio
                LR.boot.dist.bymut[boot.i] <- LR.boot.res$bymut.like.ratio
                LR.boot.top10[boot.i,] <- LR.boot.res$top.10.difs$difs
                LR.bin.boot.res <- calc.like.ratio.binomial.based(mut.mat.by.back.boot, anc.to.excl=NULL, muts.to.excl=NULL)
                LR.bin.boot.dist.bymut[boot.i] <- LR.bin.boot.res$alt.null.like.diff
                LR.bin.boot.top10[boot.i,] <- LR.bin.boot.res$top.10.difs$difs
                boot.i <- boot.i + 1
            }  # if (sum.muts.boot == sum.muts.real){
        }  # for (boot.i in ...)
        
        top.1.v.ex2 <- as.numeric(apply(LR.boot.top10, 1, function(x) max(x)))
        p.val.byanc.ex2 <- length(which(LR.boot.dist.byanc > LR.real.byanc))/n.boots
        p.val.bymut.ex2 <- length(which(LR.boot.dist.bymut > LR.real.bymut))/n.boots
        p.val.bin.bymut.ex2 <- length(which(LR.bin.boot.dist.bymut> LR.bin.real.bymut))/n.boots

        hist(LR.boot.dist.bymut, xlim=c(min(LR.boot.dist.bymut), 1.1*max(c(LR.boot.dist.bymut, LR.real.bymut))), xlab=expression(Lambda), main=expression(paste("Distribtion of ", Lambda, " under Null and Observed ", Lambda)), col="grey", breaks=10)
        mtext(side=3, line=0, text="Without 2534.G.T and 2397.A.G", font=2)
        points(LR.real.bymut,0, pch=23, bg="red")
        legend("topright", pch=23, pt.bg="red", legend="Observed")
```

```
        # --- Remove a mutation (e..g 2534.G.T and "2397.A.G") from the dataset and rerun ---
        mut.excl <- c("2534.G.T", "2397.A.G", "2332.A.G")
        # p.value = 0.007
        mut.mat.by.back.real <- get.mut.mat.by.back(muts.by.well.nb)
        LR.results <- calc.like.ratio(mut.mat.by.back.real, anc.to.excl=NULL, muts.to.excl=mut.excl)
        LR.results.bin <- calc.like.ratio.binomial.based(mut.mat.by.back.real, anc.to.excl=NULL, muts.to.excl=mut.excl)
        LR.bin.real.bymut <- LR.results.bin$alt.null.like.diff
        sum.muts.real <- LR.results$sum.of.muts
        LR.real.byanc <- LR.results$byanc.like.ratio
        LR.real.bymut <- LR.results$bymut.like.ratio
        bymut.like.diffs <- LR.results$bymut.like.diff
    
        n.boots <- 100
        LR.boot.dist.byanc <- rep(NA, n.boots)
        LR.boot.dist.bymut <- rep(NA, n.boots)
        LR.boot.top10 <- matrix(nrow=n.boots, ncol=10)
        #LR.bin.boot.dist.byanc <- rep(NA, n.boots)
        LR.bin.boot.dist.bymut <- rep(NA, n.boots)
        LR.bin.boot.top10 <- matrix(nrow=n.boots, ncol=10)
        boot.i <- 1
        
        while (boot.i <= n.boots){
            muts.by.well.boot <- randomize.muts.by.well.matrix(muts.by.well.nb, reversion.v, reversion2.v, anc.to.excl=NULL, muts.to.excl=mut.excl)
            sum.muts.boot <- sum(muts.by.well.boot)
            if (sum.muts.boot == sum.muts.real){
                #print (boot.i)
                mut.mat.by.back.boot <- get.mut.mat.by.back(muts.by.well.boot)
                LR.boot.res <- calc.like.ratio(mut.mat.by.back.boot, anc.to.excl=NULL, muts.to.excl=NULL)
                LR.boot.dist.byanc[boot.i] <- LR.boot.res$byanc.like.ratio
                LR.boot.dist.bymut[boot.i] <- LR.boot.res$bymut.like.ratio
                LR.boot.top10[boot.i,] <- LR.boot.res$top.10.difs$difs
                LR.bin.boot.res <- calc.like.ratio.binomial.based(mut.mat.by.back.boot, anc.to.excl=NULL, muts.to.excl=NULL)
                LR.bin.boot.dist.bymut[boot.i] <- LR.bin.boot.res$alt.null.like.diff
                LR.bin.boot.top10[boot.i,] <- LR.bin.boot.res$top.10.difs$difs
                boot.i <- boot.i + 1
            }  # if (sum.muts.boot == sum.muts.real){
        }  # for (boot.i in ...)
        
        top.1.v.ex3 <- as.numeric(apply(LR.boot.top10, 1, function(x) max(x)))
        p.val.byanc.ex3 <- length(which(LR.boot.dist.byanc > LR.real.byanc))/n.boots
        p.val.bymut.ex3 <- length(which(LR.boot.dist.bymut > LR.real.bymut))/n.boots
        p.val.bin.bymut.ex3 <- length(which(LR.bin.boot.dist.bymut> LR.bin.real.bymut))/n.boots     
        
        hist(LR.boot.dist.bymut, xlim=c(min(LR.boot.dist.bymut), 1.1*max(c(LR.boot.dist.bymut, LR.real.bymut))), xlab=expression(Lambda), main=expression(paste("Distribtion of ", Lambda, " under Null and Observed ", Lambda)), col="grey", breaks=10)
        mtext(side=3, line=0, text="Without 2534.G.T, 2397.A.G and 2332.A.G", font=2)
        points(LR.real.bymut,0, pch=23, bg="red")
        legend("topright", pch=23, pt.bg="red", legend="Observed")
```

```
        # --- Remove c("2534.G.T", "2397.A.G", "2332.A.G", "2100.T.C") from the dataset and rerun ---
        # p.value = 0.04
        n.boots <- 100
        mut.excl <- c("2534.G.T", "2397.A.G", "2332.A.G", "2100.T.C")
        mut.mat.by.back.real <- get.mut.mat.by.back(muts.by.well.nb)
        LR.results <- calc.like.ratio(mut.mat.by.back.real, anc.to.excl=NULL, muts.to.excl=mut.excl)
        LR.results.bin <- calc.like.ratio.binomial.based(mut.mat.by.back.real, anc.to.excl=NULL, muts.to.excl=mut.excl)
        LR.bin.real.bymut <- LR.results.bin$alt.null.like.diff
        sum.muts.real <- LR.results$sum.of.muts
        LR.real.byanc <- LR.results$byanc.like.ratio
        LR.real.bymut <- LR.results$bymut.like.ratio
        bymut.like.diffs <- LR.results$bymut.like.diff
    
        LR.boot.dist.byanc <- rep(NA, n.boots)
        LR.boot.dist.bymut <- rep(NA, n.boots)
        LR.boot.top10 <- matrix(nrow=n.boots, ncol=10)
        #LR.bin.boot.dist.byanc <- rep(NA, n.boots)
        LR.bin.boot.dist.bymut <- rep(NA, n.boots)
        LR.bin.boot.top10 <- matrix(nrow=n.boots, ncol=10)
        boot.i <- 1
        
        while (boot.i <= n.boots){
            muts.by.well.boot <- randomize.muts.by.well.matrix(muts.by.well.nb, reversion.v, reversion2.v, anc.to.excl=NULL, muts.to.excl=mut.excl)
            sum.muts.boot <- sum(muts.by.well.boot)
            if (sum.muts.boot == sum.muts.real){
                #print (boot.i)
                mut.mat.by.back.boot <- get.mut.mat.by.back(muts.by.well.boot)
                LR.boot.res <- calc.like.ratio(mut.mat.by.back.boot, anc.to.excl=NULL, muts.to.excl=NULL)
                LR.boot.dist.byanc[boot.i] <- LR.boot.res$byanc.like.ratio
                LR.boot.dist.bymut[boot.i] <- LR.boot.res$bymut.like.ratio
                LR.boot.top10[boot.i,] <- LR.boot.res$top.10.difs$difs
                LR.bin.boot.res <- calc.like.ratio.binomial.based(mut.mat.by.back.boot, anc.to.excl=NULL, muts.to.excl=NULL)
                LR.bin.boot.dist.bymut[boot.i] <- LR.bin.boot.res$alt.null.like.diff
                LR.bin.boot.top10[boot.i,] <- LR.bin.boot.res$top.10.difs$difs
                boot.i <- boot.i + 1
            }  # if (sum.muts.boot == sum.muts.real){
        }  # for (boot.i in ...)
        
        top.1.v.ex4 <- as.numeric(apply(LR.boot.top10, 1, function(x) max(x)))
        p.val.byanc.ex4 <- length(which(LR.boot.dist.byanc > LR.real.byanc))/n.boots
        p.val.bymut.ex4 <- length(which(LR.boot.dist.bymut > LR.real.bymut))/n.boots
        p.val.bin.bymut.ex4 <- length(which(LR.bin.boot.dist.bymut> LR.bin.real.bymut))/n.boots 

            hist(LR.boot.dist.bymut, xlim=c(min(LR.boot.dist.bymut), 1.1*max(c(LR.boot.dist.bymut, LR.real.bymut))), xlab=expression(Lambda), main=expression(paste("Distribtion of ", Lambda, " under Null and Observed ", Lambda)), col="grey", breaks=10)
        mtext(side=3, line=0, text="Without 2534.G.T, 2397.A.G, 2332.A.G and 2100.T.C", font=2)
        points(LR.real.bymut,0, pch=23, bg="red")
        legend("topright", pch=23, pt.bg="red", legend="Observed")
```

```
# --- Remove c("2534.G.T", "2397.A.G", "2332.A.G", "2100.T.C", 3561.T.C) from the dataset and rerun ---
        # p.value = 0.104
        n.boots <- 100
        mut.excl <- c("2534.G.T", "2397.A.G", "2332.A.G", "2100.T.C", "3561.T.C")
        mut.mat.by.back.real <- get.mut.mat.by.back(muts.by.well.nb)
        LR.results <- calc.like.ratio(mut.mat.by.back.real, anc.to.excl=NULL, muts.to.excl=mut.excl)
        LR.results.bin <- calc.like.ratio.binomial.based(mut.mat.by.back.real, anc.to.excl=NULL, muts.to.excl=mut.excl)
        LR.bin.real.bymut <- LR.results.bin$alt.null.like.diff
        sum.muts.real <- LR.results$sum.of.muts
        LR.real.byanc <- LR.results$byanc.like.ratio
        LR.real.bymut <- LR.results$bymut.like.ratio
        bymut.like.diffs <- LR.results$bymut.like.diff
    
        LR.boot.dist.byanc <- rep(NA, n.boots)
        LR.boot.dist.bymut <- rep(NA, n.boots)
        LR.boot.top10 <- matrix(nrow=n.boots, ncol=10)
        #LR.bin.boot.dist.byanc <- rep(NA, n.boots)
        LR.bin.boot.dist.bymut <- rep(NA, n.boots)
        LR.bin.boot.top10 <- matrix(nrow=n.boots, ncol=10)
        boot.i <- 1
        
        while (boot.i <= n.boots){
            muts.by.well.boot <- randomize.muts.by.well.matrix(muts.by.well.nb, reversion.v, reversion2.v, anc.to.excl=NULL, muts.to.excl=mut.excl)
            sum.muts.boot <- sum(muts.by.well.boot)
            if (sum.muts.boot == sum.muts.real){
                #print (boot.i)
                mut.mat.by.back.boot <- get.mut.mat.by.back(muts.by.well.boot)
                LR.boot.res <- calc.like.ratio(mut.mat.by.back.boot, anc.to.excl=NULL, muts.to.excl=NULL)
                LR.boot.dist.byanc[boot.i] <- LR.boot.res$byanc.like.ratio
                LR.boot.dist.bymut[boot.i] <- LR.boot.res$bymut.like.ratio
                LR.boot.top10[boot.i,] <- LR.boot.res$top.10.difs$difs
                LR.bin.boot.res <- calc.like.ratio.binomial.based(mut.mat.by.back.boot, anc.to.excl=NULL, muts.to.excl=NULL)
                LR.bin.boot.dist.bymut[boot.i] <- LR.bin.boot.res$alt.null.like.diff
                LR.bin.boot.top10[boot.i,] <- LR.bin.boot.res$top.10.difs$difs
                boot.i <- boot.i + 1
            }  # if (sum.muts.boot == sum.muts.real){
        }  # for (boot.i in ...)
        
        top.1.v.ex5 <- as.numeric(apply(LR.boot.top10, 1, function(x) max(x)))
        p.val.byanc.ex5 <- length(which(LR.boot.dist.byanc > LR.real.byanc))/n.boots
        p.val.bymut.ex5 <- length(which(LR.boot.dist.bymut > LR.real.bymut))/n.boots
        p.val.bin.bymut.ex5 <- length(which(LR.bin.boot.dist.bymut> LR.bin.real.bymut))/n.boots 
        
        hist(LR.boot.dist.bymut, xlim=c(min(LR.boot.dist.bymut), 1.1*max(c(LR.boot.dist.bymut, LR.real.bymut))), xlab=expression(Lambda), main=expression(paste("Distribtion of ", Lambda, " under Null and Observed ", Lambda)), col="grey", breaks=10)
        mtext(side=3, line=0, text="Without 2534.G.T, 2397.A.G, 2332.A.G, 2100.T.C and 3561.T.C", font=2)
        points(LR.real.bymut,0, pch=23, bg="red")
        legend("topright", pch=23, pt.bg="red", legend="Observed")
```

#### Create Figure 7 Showing Mutation by Background Epistasis

```
  file.name <- "Mutations_by_background_figure_March_2016"
    generate.plot.of.mut.by.background.occurence(file.name, "pdf")
```

```
## quartz_off_screen 
##                 2
```

```
    generate.plot.of.mut.by.background.occurence(file.name, "svg")
```

```
## quartz_off_screen 
##                 2
```

**Figure 7 in Paper. Evidence of background epistasis is significant but rare.** Main plot shows the number of wells each mutation appears in with a heatmap scale above the plot. Within cells, “rev” indicates the mutation is a reversion and is excluded from statistical testing. The barplot to the right gives the difference in log-likelihood (\(\Delta lnL\)) between the null (no epistasis) and the alterantive (epistasis) model for each mutation. Globally, the no-background effect null is rejected (\(p<0.001\)). To determine which mutations are significant, they were removed from the dataset from the largest downward in a cumulative fashion. Removal of the mutations with red bars still resulted in significant results (\(^{\*\*\*} p<0.001\), \(^{\*\*} p<0.01\), \(^\* p<0.05\)). Removal of the red mutations plus the mutation in black results in a non-significant result (\(p\approx0.104\)).

#### Epistasis between de novo mutations

Reversions are excluded from this analysis.

```
    # Three pieces to this code: (1) Find when mutations co-occur in well, how often do they co-occur in isolate = mean.coij; (2) Get observed pattern of co-occurence; (3) Get expected pattern of co-occurence.
    
    # === (1) When two mutations occur in same well, how often do they occur in same isolate?
    mut.mat.nb.info <- matrix(nrow=length(rownames(mut.matrix.nb)), ncol=3)
    wells.nb <- unlist(lapply(rownames(mut.matrix.nb), function(x) paste(unlist(strsplit(x, split="_"))[1:2], collapse="_")))
    anc.nb <- unlist(lapply(rownames(mut.matrix.nb), function(x) paste(unlist(strsplit(x, split="_"))[3], collapse="_")))
    iso.nb <- unlist(lapply(rownames(mut.matrix.nb), function(x) paste(unlist(strsplit(x, split="_"))[4:6], collapse="_")))
    prob.coij.bywell <- rep(NA, n.wells)
    names(prob.coij.bywell) <- wells.by.back$Well
    
    for (well.i in 1:n.wells){
        well.nm <- as.character(wells.by.back$Well[well.i])
        tree.rows <- which(tree.data$Well==well.nm)
        sub.data <- tree.data[tree.rows,]
        u.muts <- sub.data$mut[2:length(sub.data$mut)]
        nu.muts <- length(u.muts)
        co.m <- matrix(nrow=nu.muts, ncol=nu.muts, data=NA)
        colnames(co.m) <- rownames(co.m) <- u.muts
        
        nb.rows <- which(wells.nb==well.nm)
        for (i in 1:(nu.muts-1))        {
            mut.i <- as.character(u.muts[i])
            i.dex <- which(unq.muts==mut.i)
            for (j in (i+1):nu.muts){
                mut.j <- as.character(u.muts[j])
                j.dex <- which(unq.muts == mut.j)
                co.ij <- as.numeric(length(which(apply(mut.matrix.nb[nb.rows, c(i.dex, j.dex)], 1, sum)==2))>0)
                co.m[i,j] <- co.ij
                n.z <- length(which(co.m ==0))
                n.1 <- length(which(co.m==1))
                p.1 <- n.1/(n.z + n.1)  
                prob.coij.bywell[well.i] <- p.1
            }
        }
        
    }  # next well.i
    mean.coij <- mean(prob.coij.bywell)  
    
    
  #=== (2) and (3) === packaged into functions
    obs.results <- get.mut.co.occurence(mut.matrix.nb)  # ===(2) Now on to the co-occureance summary of the real data ===
    muts.co.occur <- obs.results
    exp.results <- get.mut.exp.co.occurence(mut.mat.by.back.real, obs.results)  # === (3) Get expected co-occurence ===
    muts.co.occur.exp <- exp.results$muts.co.occur.exp
    muts.co.occur.exp2 <- exp.results$muts.co.occur.exp
    p.co.occur2 <- exp.results$p.muts.co.occur
    o <- order(p.co.occur2)
    low.5 <- p.co.occur2[o[1:5]]
    low5.pairs <- lapply(low.5, function(x) which(p.co.occur2 == x, arr.ind=TRUE))
```

#### Bootstrap to look for significance of deviations from expectation

```
    if (bootstrap.cocount.sig.bool==TRUE){
    # === bootstrap to look for significance ===
    sum.real.obs <- sum(muts.by.well.nb)
    mut.mat.by.back.real <- get.mut.mat.by.back(muts.by.well.nb)
    LR.results <- calc.like.ratio(mut.mat.by.back.real, anc.to.excl=NULL, muts.to.excl=NULL)
    sum.muts.real <- LR.results$sum.of.muts
    obs.co.occur <- get.mut.co.occurence(mut.matrix.nb)

    n.boots <- 100
    low.p.by.boot <- matrix(nrow=n.boots, ncol=5)
    co.16.17 <- rep(NA, n.boots)
    co.16.32 <- rep(NA, n.boots)
    co.17.32 <- rep(NA, n.boots)
    co.count.list <- vector(mode="list", length=n.boots)   # a list with the co-occurence count matrix from each boot
    
    boot.i <- 1
    while (boot.i <= n.boots){
        print(boot.i)
        boot.res <- randomize.muts.by.isolate.matrix(muts.by.well.nb, mut.matrix.nb, reversion.v, reversion2.v, anc.to.excl=NULL, muts.to.excl=NULL)
        sum.boot.obs <- sum(boot.res$muts.by.well.new)
  
        if (sum.boot.obs == sum.muts.real){
            muts.co.occur.boot <- get.mut.co.occurence(boot.res$mut.matrix.new)
            #muts.co.occur.exp.boot <- get.mut.exp.co.occurence(boot.res$muts.by.back, muts.co.occur.boot)
            #p.co.occur.boot <- muts.co.occur.exp.boot$p.muts.co.occur
            #low5.boot <- p.co.occur.boot[order(p.co.occur.boot)[1:5]]
            #low5.boot.pairs <- lapply(low5.boot, function(x) which(p.co.occur.boot == x, arr.ind=TRUE))
            #co.16.17[boot.i] <- muts.co.occur.boot[16,17]
            co.count.list[[boot.i]] <- muts.co.occur.boot
            boot.i <- boot.i+1
          }
    }
    
    d <- dim(co.count.list[[1]])[1]
      co.count.list.m <- matrix(nrow=0, ncol=d+1)
      for (i in 1:n.boots){
        m <- cbind(boot=rep(i, d), co.count.list[[i]])
        co.count.list.m <- rbind(co.count.list.m, m)
    }
    write.table(co.count.list.m, file="co.count.list.matrix.txt", sep="\t", col.names=TRUE, row.names=FALSE)
    
    } else{        # ---If not geneating the list, read it in here and transform it from a big matrix back into a list.
      obs.co.occur <- get.mut.co.occurence(mut.matrix.nb)
      co.count.list.m <- read.table(file="co.count.list.matrix.txt", header=TRUE)
      mut.names <- sapply(colnames(co.count.list.m), function(x) unlist(strsplit(x, "X"))[2])
      mut.names <- mut.names[-1]
      n.boots <- max(co.count.list.m$boot)
      co.count.list <- vector("list", n.boots)
      for (i in 1:n.boots){
        co.count.list[[i]] <- co.count.list.m[which(co.count.list.m$boot==i),]
        co.count.list[[i]] <- co.count.list[[i]][-1]
        colnames(co.count.list[[i]]) <- rownames(co.count.list[[i]]) <- mut.names
      }
    }
    

    # We are not very intersted in pairs with expectations < 1 as these are an obvious
    # source of false positive. So here we identify pairs where the expecation is > 1.
    
    mean.co.null.m <- matrix(nrow=length(obs.co.occur[,1]), ncol=length(obs.co.occur[1,]), data=0)
    colnames(mean.co.null.m) <- colnames(obs.co.occur)
    rownames(mean.co.null.m) <- rownames(obs.co.occur)
    for (mut.i in 1:(length(obs.co.occur[,1])-1)){
        for (mut.j in mut.i:length(obs.co.occur[,1])){
            mean.co.null.m[mut.i, mut.j] <- mean(unlist(lapply(co.count.list, function(x) x[mut.i, mut.j])), na.rm=TRUE)
        }
    }
    m.obs.muts <- which(well.counts.by.mut > 1)
    
    
    # ---- And now we get p-values for just these mutations.
    pvals.co.m <- matrix(nrow=length(m.obs.muts), ncol=length(m.obs.muts), data=NA)
    colnames(pvals.co.m) <- names(m.obs.muts)
    rownames(pvals.co.m) <- names(m.obs.muts)
    for (i in 1:(length(m.obs.muts)-1)){
        pvals.co.m[i,i]  <- NA
        for (j in (i+1):length(m.obs.muts)){
            pvals.co.m[j,j]  <- NA
            obs.c <- obs.co.occur[m.obs.muts[i], m.obs.muts[j]]
            null.c <- unlist(lapply(co.count.list, function(x) x[m.obs.muts[i], m.obs.muts[j]]))
            if (is.na(obs.c)==FALSE){
                null.leq <- length(which(null.c <= obs.c))/n.boots
                null.geq <- length(which(null.c >= obs.c))/n.boots
                null.eq <-  length(which(null.c == obs.c))/n.boots
                pvals.co.m[i,j] <-min(2*min(c(null.leq, null.geq, null.eq)),1)
            } else{
                pvals.co.m[i,j] <- pvals.co.m[j,i] <- NA
            }
        }
    }
    
    
    
    # --- Now we dig into the D-promoter mutations ---
    D.pro.dex <- sapply(D.pro.muts, function(x) which(names(well.counts.by.mut)==x))
    Near.muts <- which(names(well.counts.by.mut) %in% c("1914.A.G", "1927.G.T"))
  D.pro.dex2 <- c(D.pro.dex, Near.muts)
    
    sum.above.diag <- function(mat){
        for (i in 1:(length(mat[,1])-1)){
            mat[i:length(mat[1,]),i] <- NA
        }
        sum.mat <- sum(mat, na.rm=TRUE)
        return(sum.mat)
    }
    D.pro.co.real <- sum.above.diag(obs.co.occur[D.pro.dex, D.pro.dex])
    D.pro.co.boot <- sapply(co.count.list, function(x) sum.above.diag(x[D.pro.dex, D.pro.dex]))
    # A look at D.pro.co.boot shows that boostrap replicates never get even close to having D.pro muts not co-occur. Min in 100 bootstraps = 9 or 10.
  hist(D.pro.co.boot, col="grey", xlim=c(0,30), xlab="Co-occurence of D-promoter mutations", main="Observed vs Bootstrap Co-Occurence of D-promoter mutations")
  points(D.pro.co.real, 0, pch=23, bg="red", cex=2)
  legend("topleft", legend=c("Observed", "Bootstrap (null)"), pch=c(23,22), pt.bg=c("red", "grey"), pt.cex=c(1,2))
```

```
  # --- Get p-values associated D-pro mutations ---
  # This shows that only 2 mutations are significant with the D.pro.block: 2134.T.C (p=0) and 2534.G.T (p=0)
  p.vals.with.D.pro.block <- rep(NA, length(m.obs.muts))
    names(p.vals.with.D.pro.block) <- names(m.obs.muts)
    for (i in 8:length(m.obs.muts)){
        obs.sum <- sum(obs.co.occur[D.pro.dex, m.obs.muts[i]])
        dist.sum <- sapply(co.count.list, function(x) sum(x[D.pro.dex, m.obs.muts[i]])) 
        if (is.na(obs.sum)==FALSE){
            null.leq <- length(which(dist.sum <= obs.sum))/n.boots
            null.geq <- length(which(dist.sum >= obs.sum))/n.boots
            null.eq <-  length(which(dist.sum == obs.sum))/n.boots
            p.vals.with.D.pro.block[i] <-min(2*min(c(null.leq, null.geq, null.eq)),1)
        }
    }
    
    
    # --- This identifies our significant associations. 
    #     Pulled from pvals.co.m and p.vals.with.D.pro.block and 
    D.pro.block.sig.muts <- c("2134.T.C", "2534.G.T")   
    sig.pair.muts <- vector("list", 4)
    sig.pair.muts[[1]] <- c("1910.A.G", "1911.C.T")
    sig.pair.muts[[2]] <- c("1910.A.G", "2134.T.C")
    sig.pair.muts[[3]] <- c("1910.A.G", "2577.C.T")
    sig.pair.muts[[4]] <- c("2534.G.T", "2630.G.T")

      
    co.16.17.32 <- matrix(nrow=n.boots, ncol=5)
    colnames(co.16.17.32) <- c("16.17", "16.32", "17.32", "Dpro", "Dpro.2134")
    D.pro.muts <- as.character(mut.nuc.AA.m $mut.DNA[which(mut.nuc.AA.m$in.feature=="D.pro")])
    D.pro.dexs <- unlist(lapply(D.pro.muts, function(x) which(unq.muts==x)))
    
    # === Co-occurences in the most inmportant trio of mutation == 
    for (boot.i in 1:n.boots){
        co.16.17.32[boot.i,1]    <- co.count.list[[boot.i]][16,17]
        co.16.17.32[boot.i,2]    <- co.count.list[[boot.i]][16,32]
        co.16.17.32[boot.i,3]    <- co.count.list[[boot.i]][17,32]
        sum.Dpro <- 0
        co.16.17.32[boot.i,5] <- sum(co.count.list[[boot.i]][D.pro.dexs,32])
        for (i in 1:(length(D.pro.dexs)-1)){
            for (j in (i+1): length(D.pro.dexs)){
                sum.Dpro <- sum.Dpro + co.count.list[[boot.i]][D.pro.dexs[i], D.pro.dexs[j]]
            }
        }
        co.16.17.32[boot.i,4] <- sum.Dpro
    }
        
    mutcounts.real <- colSums(mut.mat.by.back.real)
    
    # === This gives co-occurence b/t each of the muts observed >= 6 times in real data w/ D-promoter mutations
    co.D.ge6 <- matrix(nrow=n.boots, ncol=6)
    comp.muts <- c(32,47,57,64,69,88)
    colnames(co.D.ge6) <- unq.muts[c(32,47,57,64,69,88)]
    for (boot.i in 1:n.boots){
        for (j in 1:length(comp.muts)){
            co.D.ge6[boot.i,j] <- sum(co.count.list[[boot.i]] [D.pro.dexs, comp.muts[j] ])
        }
    }
    
    unq.muts.noD <- unq.muts[-D.pro.dexs]
    unq.noD.dexs <- seq(1, n.muts)
    unq.noD.dexs <- unq.noD.dexs[-D.pro.dexs]
    co.D.all <- matrix(nrow=n.boots, ncol=length(unq.muts.noD))
    colnames(co.D.all) <- unq.muts.noD
    for (boot.i in 1:n.boots){
        for (j in 1:length(unq.muts.noD)){
            co.D.all[boot.i,j] <- sum(co.count.list[[boot.i]] [D.pro.dexs, unq.noD.dexs[j] ])
        }
    }   
    mean.co.D.all <- colMeans(co.D.all)   # this gives the mean co-occurences b/t each mutation and any 1 D-promoter mutation


    # === The mean co-occurences over set of all boots ===
    co.means.mins <- matrix(nrow=n.muts, ncol=n.muts)
    colnames(co.means.mins) <- rownames(co.means.mins) <- unq.muts
    for (i in 1:(n.muts-1)){
        for (j in (i+1):n.muts){
            obs.v <- NULL
            for (boot.i in 1:n.boots){
                obs.v <- c(obs.v, co.count.list[[boot.i]][i,j])
                co.means.mins[i,j] <- mean(obs.v)
            }
        }
    }
    
    # === Total Co-occurences b/t pairs of D-promoter mutations ---
    D.pro.co <- rep(NA, n.boots)
    for (boot.i in 1:n.boots){
        total <- 0
        for (i in 1:(length(D.pro.dexs)-1)){
            for (j in (i+1):length(D.pro.dexs)){
                total <- total+co.count.list[[boot.i]][D.pro.dexs[i], D.pro.dexs[j]]
            }
        }
        D.pro.co[boot.i] <- total
    }
    mean.D.D.co <- mean(D.pro.co)
    
    
    file.name <- "mutation_co-occurence_3"
    create.cooccurence.plot(file.name, "pdf")
```

```
## quartz_off_screen 
##                 2
```

```
    create.cooccurence.plot(file.name, "svg")
```

```
## quartz_off_screen 
##                 2
```

**Figure 8 in Paper: Pattern of observed vs expected co-occurences.** Most importantly, the plot reveals that D-promoter mutations never co-occur nor do they occur with several other mutations including 2134cT, 2094aG, 2131tC, and 2158aG. Each cell shows the observed count in the lower left vs the expected count in the upper right for that comparison. Larger counts are shaded more darkly to show the overall pattern and significant differences (\(p < 0.05\)) are highlighted in red. The D-promoter mutations are at sites 1909-1935 (first 7 mutations) and the is this collection of mutations. \*The D-pro block comparison with itself shows the total number of observed and expected wells where two different D-promoter mutations co-occur in the same isolate; all other comparisons with D-pro block are observed and expected co-occurences with any of the D-promoter mutations. Singletons and reversions have been removed from the figure and expected counts have been rounded to nearest integer. Expected counts are based on a bootstrap randomization procedure (see methods).}}

## Analysis of Lysis Data

```
  data.all <- read.table(file=paste(getwd(), "/Data/ID11 Lysis Time Data Lu 18Dec14.txt", sep=""), row.names=1, header=TRUE)
  count.to.use <- "raw.count"  # rel.count or raw.count
  
  std.err <- function(counts){
      std.err <- sd(counts)/sqrt(length(counts))
      return(std.err)
  }

  data.all$Minute <- as.numeric(substring(data.all$Minute, 1 ,2))
  Backs <- unique(data.all$Background)
  mut.cols <- as.data.frame(matrix(nrow=5, ncol=2))
  colnames(mut.cols) <- c("mutation", "color")
  mut.cols$mutation <- c("1911cT", "1910aG", "2131cT", "2134tC", "3134cT")
  mut.cols$color <- c("deepskyblue", "red", "yellow", "green", "black")
  
  file.name <- "Lysis_Time_Figure"
  create.lysis.time.plot(file.name, "pdf")
```

```
## quartz_off_screen 
##                 2
```

```
  create.lysis.time.plot(file.name, "svg")
```

```
## quartz_off_screen 
##                 2
```

**Figure 9 from Paper. Time to lysis is delayed by the assayed mutations across backgrounds.**} Each panel presents a different genetic background: A=J15, B=J20, C=F178, and D=ID11 wild type. Plotted are the means and standard errors of all observed burst counts for each genotype at each time point.

## Mechanisms of Lysis Delay

#### D-promoter Anlaysis

```
    promo.energy <- read.table(file=paste(getwd(), "/Model_input_files/polymerase_energy_matrix_2.txt", sep=""), sep="\t", header=TRUE)
    promo.energy.r <- promo.energy[order(as.numeric(rownames(promo.energy)), decreasing=TRUE),]
    D.pro.m10 <- c(ID11.features$start[which(ID11.features$feature.detail=="D.pro.m10")], ID11.features$end[which(ID11.features$feature.detail=="D.pro.m10")])
    D.pro.m35 <- c(ID11.features$start[which(ID11.features$feature.detail=="D.pro.m35")], ID11.features$end[which(ID11.features$feature.detail=="D.pro.m35")])
    D.pro.m10.seq <- substring(ID.11, D.pro.m10[1], D.pro.m10[2])
    D.pro.m35.seq <- substring(ID.11, D.pro.m35[1], D.pro.m35[2])
      
  D.pro.start <- ID11.features$start[which(ID11.features$feature.detail=="D.pro.m35")] -5
    D.pro.end <- ID11.features$end[which(ID11.features$feature.detail=="D.pro.m10")] + 6
    Dpro.seq.wt <- substring(ID.11, D.pro.start, D.pro.end)
    Dpro.seq.wt.v <- unlist(strsplit(as.character(Dpro.seq.wt), ""))
    promo.cols <- colnames(promo.energy)
    Dpro.seq.wt.cols <- sapply(Dpro.seq.wt.v, function(x) which(promo.cols==x))
  Dpro.eng.wt <- sapply(seq(1, 41), function(i) promo.energy[i, Dpro.seq.wt.cols[i]])   
  
  file.name <- "Promoter_binding_plot"
  create.D.promoter.strength.plot(file.name, "pdf")
```

```
## quartz_off_screen 
##                 2
```

```
  create.D.promoter.strength.plot(file.name, "svg")
```

```
## quartz_off_screen 
##                 2
```

**Table 3 from Supplement. Predicted mutational effects on binding energy profile across the D-promoter.** Plot shows the predicted effect of each D-promoter mutation on the binding affinity of RNA polymerase based on the thermodynamic model of Brewster et al (2012). Mutations are scaled by the number of wells they appeared in. The wildtype sequence is given along the x-axis.

#### Identify the D/E mutations found with vs in repulsion with D-promoter mutations

```
  D.pro.muts <- which(mut.nuc.AA.m$feat.cat == "D.pro")
    D.muts <- which(mut.nuc.AA.m$gene.1 == "D")
    E.muts <- which(mut.nuc.AA.m$gene.1 == "E")
    DE.muts <- c(D.muts, E.muts)
    D.pro.rows <- which(rowSums(mut.matrix.nb[,D.pro.muts])==1)
    
    DE.muts.phase <- rep(NA, length(DE.muts))
    for (mut.i in 1:length(DE.muts)){
        mut.iso.rows <- which(mut.matrix.nb[,DE.muts[mut.i]]==1)
        overlap <- sapply(mut.iso.rows, function(x) as.numeric(x %in% D.pro.rows))
        DE.muts.phase[mut.i] <- as.numeric(sum(overlap)>0)
    }
    DE.with.Dpro <- DE.muts[which(DE.muts.phase ==1)]
    DE.no.Dpro <- DE.muts[which(DE.muts.phase ==0)] 
    
    DE.muts.mech <- mut.nuc.AA.m[DE.muts,]
    DE.muts.mech <- cbind(DE.muts.mech, DE.muts.phase)
```

#### For each mutation, find effect(s) on usage

Append info as rightmost column of table with info on D/E mutations

```
    U.ratio.1 <- rep(NA, length(DE.muts))
    U.ratio.2 <- rep(NA, length(DE.muts))
    
    for (mut.i in 1:length(DE.muts)){
        U.ratio.1[mut.i] <- DE.muts.mech$mut.usage1[mut.i]/DE.muts.mech$anc.usage1[mut.i]
        U.ratio.2[mut.i] <- DE.muts.mech$mut.usage2[mut.i]/DE.muts.mech$anc.usage2[mut.i]
    }
    DE.muts.mech <- cbind(DE.muts.mech, Usage.R1=U.ratio.1, Usage.R2=U.ratio.2)
```

#### For each mutation, what is the effect on ribosomal affinity

Append info as rightmost column in DE.muts.mech (table on D/E mutations)

```
    ID11.features <- read.table(file=paste(getwd(), "/Model_input_files/ID11_gene_features_to_plot.txt", sep=""), header=TRUE)
    hex.energy <- read.table(file=paste(getwd(), "/Model_input_files/hexamer_energy.txt",sep=""), header=FALSE)
    colnames(hex.energy) <- c("hex", "ddG")
    window.rng <- c(1965, 2444)
    gene.seq <- gene.seq.wt <- DNAString(as.character(subseq(ID.11, window.rng[1], window.rng[2])))
    
    get.aSD.affinities <- function(gene.seq.h){
        aff.val <- rep(NA, length(gene.seq.h)-5)
        for (i in 1:(length(gene.seq.h)-5)){
            hex <- as.character(subseq(gene.seq.h, i, (i+5)))
            aff.val[i] <- hex.energy$ddG[which(hex.energy$hex == hex)]
        }
        return(aff.val)
    }
    
        # === For each mutation, what is the effect on ribosomal affinity   
    aff.val.wt <- get.aSD.affinities(gene.seq.wt)
    aff.max.dif <- rep(NA, length(DE.muts))
    
    for (mut.i in 1:length(DE.muts)){
        #print(mut.i)
        ID11.copy <- DNAString(as.character(ID.11))
        site <- mut.nuc.AA.m$site[DE.muts[mut.i]]
        mut.name <- mut.nuc.AA.m$mut.DNA[DE.muts[mut.i]]
        oldbase <- mut.nuc.AA.m$wt[DE.muts[mut.i]]
        newbase <- DNAString(mut.nuc.AA.m$mut[DE.muts[mut.i]])
        
        before <- subseq(ID.11, 1, site-1)
        after <- subseq(ID.11, site+1, width(ID.11))
        mut.ID.11 <- xscat(before, BStringSet(newbase), after)
        
        mut.gene.seq <- DNAString(as.character(subseq(mut.ID.11, window.rng[1], window.rng[2])))
        aff.val.mut <- get.aSD.affinities(mut.gene.seq) 
        
        rel.site <- site - window.rng[1]
        if (rel.site + 6 < window.rng[2]-window.rng[1]){
            comp <- cbind(aff.val.wt[(rel.site-6):(rel.site+6)], aff.val.mut[(rel.site-6):(rel.site+6)])
        } else {
            left <- window.rng[2]-window.rng[1] - rel.site
            comp <- cbind(aff.val.wt[(rel.site-6):(rel.site+left)], aff.val.mut[(rel.site-6):(rel.site+left)])
        }
        non.na <- which(is.na(comp[,1])==FALSE)
        which.max <- which.max(apply(comp[non.na,], 1, function(x) abs(x[2] -x[1])))
        aff.max.dif[mut.i] <- comp[which.max,2]-comp[which.max,1]
    }
    
    DE.muts.mech <- cbind(DE.muts.mech, aff.max.dif)
```

#### Get RNA Transcript Stability.

If generate.output.for.ViennaRNA is set to TRUE, then the code generates the file that the Vienna RNA software expects. Otherwise, we simply read the values in from /Data/DE\_RNA\_min\_free\_energies.txt and append as the rightmost column of DE.muts.mech.

```
    generate.output.for.ViennaRNA <- FALSE
    
    if (generate.output.for.ViennaRNA == TRUE){
      RNA.wt <- RNAString(complement(gene.seq.wt))
      RNA.wt.nc <- RNAString(gene.seq.wt)
    
      file.create("DE_mutation_seqs2.txt")
      sink("DE_mutation_seqs2.txt")
    cat(">Wildtype", append=FALSE)
    cat("\n", append=TRUE)
      cat(as.character(RNA.wt), append=TRUE)
      cat("\n", append=TRUE)

      for (mut.i in 1:length(DE.muts)){
        print(mut.i)
        ID11.copy <- DNAString(as.character(ID.11))
        site <- mut.nuc.AA.m$site[DE.muts[mut.i]]
        mut.name <- mut.nuc.AA.m$mut.DNA[DE.muts[mut.i]]
        oldbase <- mut.nuc.AA.m$wt[DE.muts[mut.i]]
        newbase <- DNAString(mut.nuc.AA.m$mut[DE.muts[mut.i]])
        
        before <- subseq(ID.11, 1, site-1)
        after <- subseq(ID.11, site+1, width(ID.11))
        mut.ID.11 <- xscat(before, BStringSet(newbase), after)
        
        mut.gene.seq <- DNAString(as.character(subseq(mut.ID.11, window.rng[1], window.rng[2])))
        mut.RNA <- RNAString(complement(mut.gene.seq))
        cat("\n", append=TRUE)
        cat(paste(">", mut.name, sep=""), append=TRUE)
        cat("\n", append=TRUE)
        cat(as.character(mut.RNA), append=TRUE)
      } 
      sink()  
    }
    
  mfe <- read.table(file=paste(getwd(), "/Data/DE_RNA_min_free_energies.txt", sep=""), header=TRUE)
    mfe.difs <- mfe$mfe[1] - mfe$mfe[2:length(mfe$mfe)]
    DE.muts.mech <- cbind(DE.muts.mech, mfe.difs)
```

#### Conduct simple tests for mechanisms and create comparative plot

```
    Usage.R.E <- c(DE.muts.mech$Usage.R2[1:length(DE.muts.mech[,1])-1], DE.muts.mech$Usage.R1[length(DE.muts.mech[,1])])
    Usage.R.D <- c(DE.muts.mech$Usage.R1[1:length(DE.muts.mech[,1])-1], NA)
    
    LR.null <- glm(DE.muts.phase ~ 1, data=DE.muts.mech, family="binomial")
    LR.Usage.R2 <- glm(DE.muts.phase  ~ Usage.R2, data=DE.muts.mech, family="binomial")
    
    muts.withD <- which(DE.muts.mech$DE.muts.phase ==1)
    muts.noD <- which(DE.muts.mech$DE.muts.phase ==0)
    ttest.Usage.RD <- t.test(x=Usage.R.D[muts.withD], y=Usage.R.D[muts.noD], mu=0, var.equal=FALSE)
    ttest.Usage.RE <- t.test(x=Usage.R.E[muts.withD], y=Usage.R.E[muts.noD], mu=0, var.equal=FALSE)
    ttest.SD.aff <- t.test(x=DE.muts.mech$aff.max.dif[muts.withD], y=DE.muts.mech$aff.max.dif[muts.noD], mu=0, var.equal=FALSE) 
    ttest.mfe <- t.test(x=DE.muts.mech$mfe.difs[muts.withD], y=DE.muts.mech$mfe.difs[muts.noD], mu=0, var.equal=FALSE)      
    
    
    file.name <- "Delay_mech_dotplots"
    create.lysis.delay.dotplot.figure(file.name, "pdf")
```

```
## quartz_off_screen 
##                 2
```

```
    create.lysis.delay.dotplot.figure(file.name, "svg")
```

```
## quartz_off_screen 
##                 2
```

**Figure 4 in Supplement. Comparison of molecular properties for mutations in the genes D/E transcript in repulsion with D-promoters mutation (yellow) vs those found with D-promoter mutations (red).** (A) Mutant codon usage relative to wild type in gene D. (B) Mutant codon usage relative to wild type in gene E. (C) Maximum change in Shine-Dalgarno affinity about the mutation site. (D) Change in minimum free energy (stability) of the transcript. The assayed mutations 2131cT and 2134cT are denoted with and respectively.
