# Supplementary material for: Love the one you’re with: replicate viral adaptations converge on the same phenotypic change
Source: PeerJ. 2016 Jul 19;4:e2227. doi: 10.7717/peerj.2227 (PMC4958007; doi:10.7717/peerj.2227)
Supplement: Supplemental Information 3 [file peerj-04-2227-s003.zip › Markdown_detailed_anlaysis_code_and_data_minor_revisions/Parallelism_between_Wells_2.pdf]

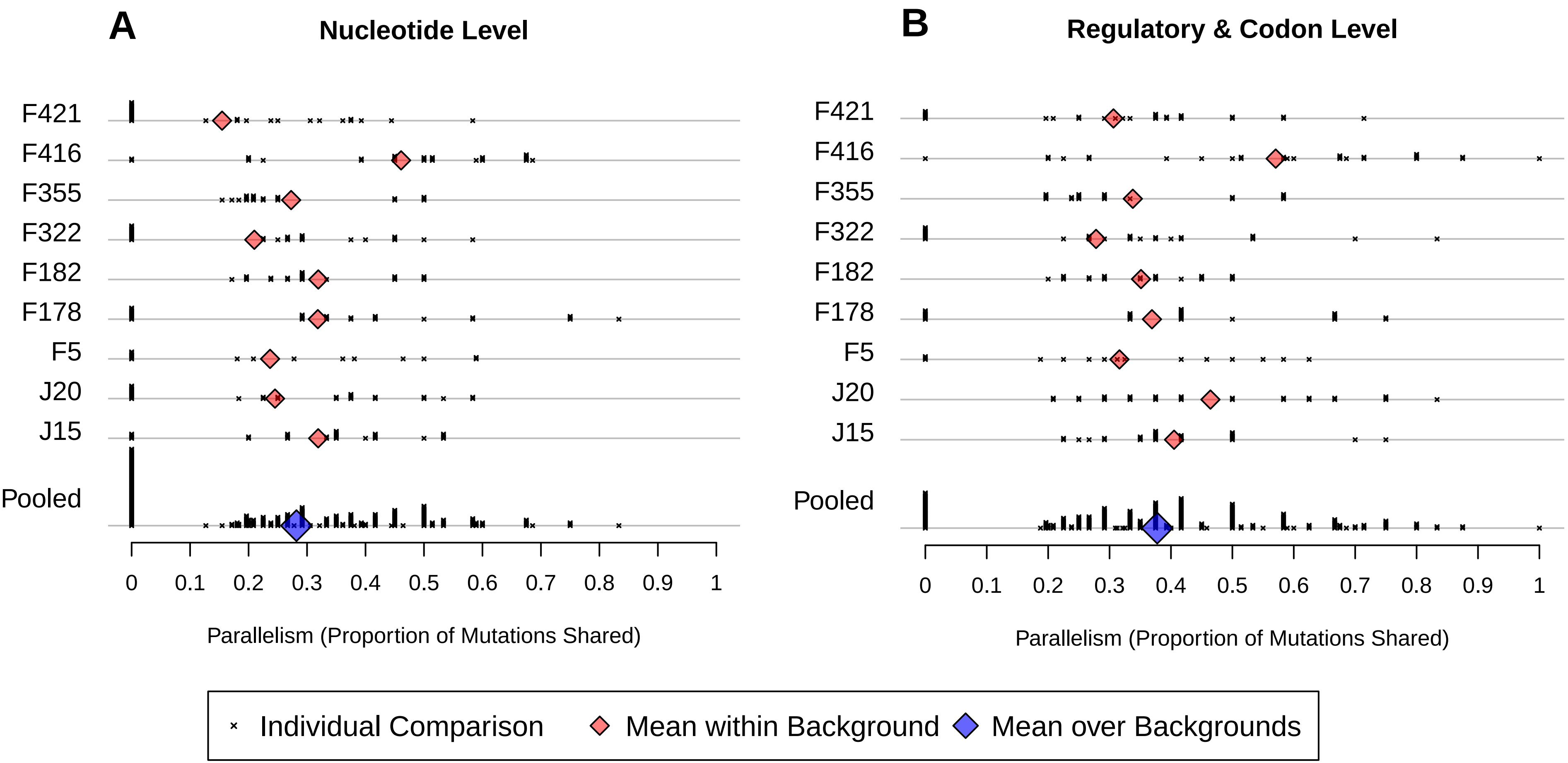

A
Nucleotide Level
B
Regulatory & Codon Level
F421
F416
F355
F322
F182
F178
F5
J20
J15
Pooled
0
0.1
0.2
0.3
0.4
0.5
0.6
0.7
0.8
0.9
1
Parallelism (Proportion of Mutations Shared)
Individual Comparison
Mean within Background
Mean over Backgrounds
