# Supplementary figures and images for: Love the one you’re with: replicate viral adaptations converge on the same phenotypic change
Source: PeerJ. 2016 Jul 19;4:e2227. doi: 10.7717/peerj.2227 (PMC4958007; doi:10.7717/peerj.2227)

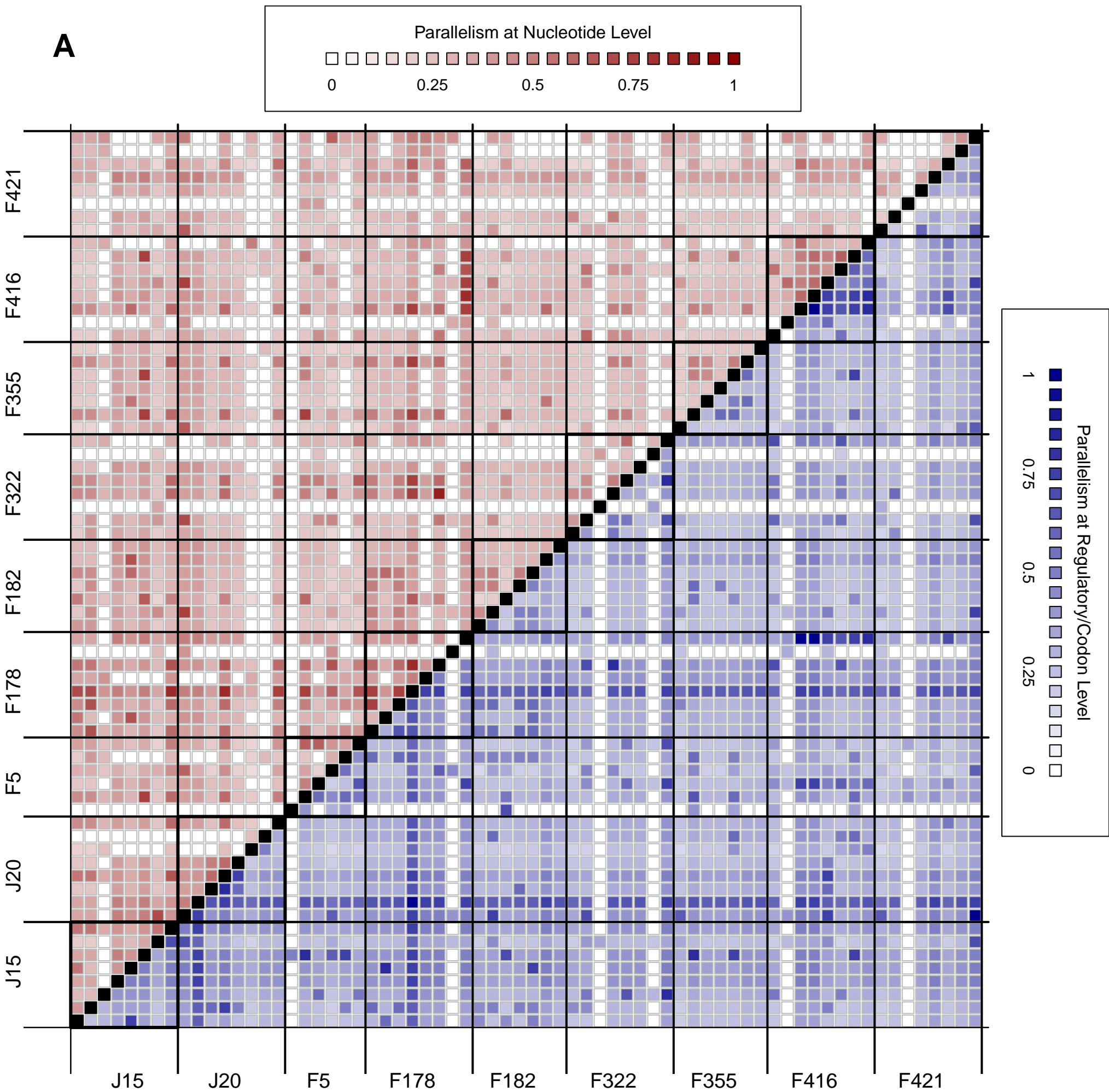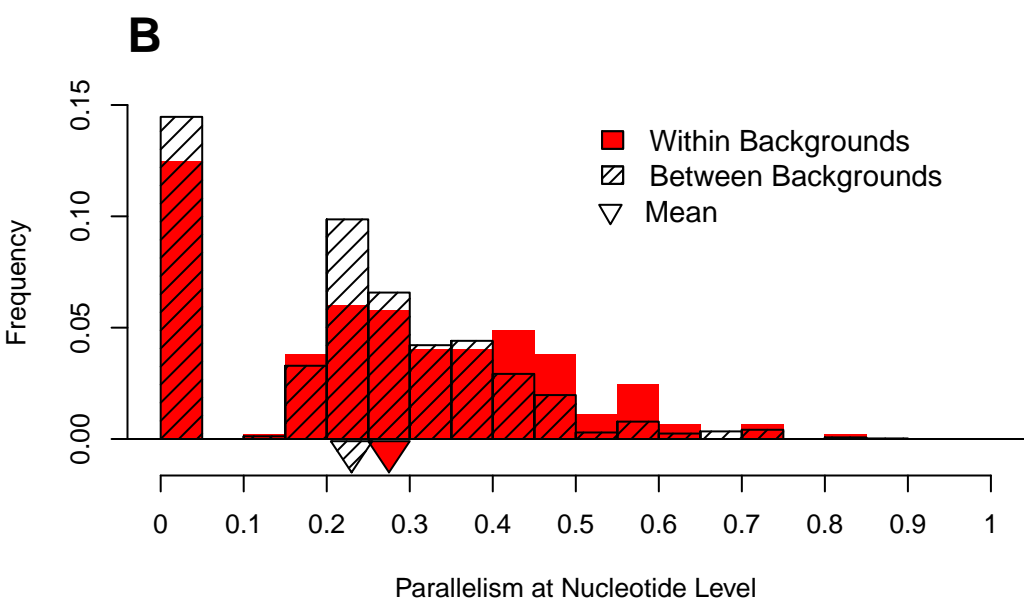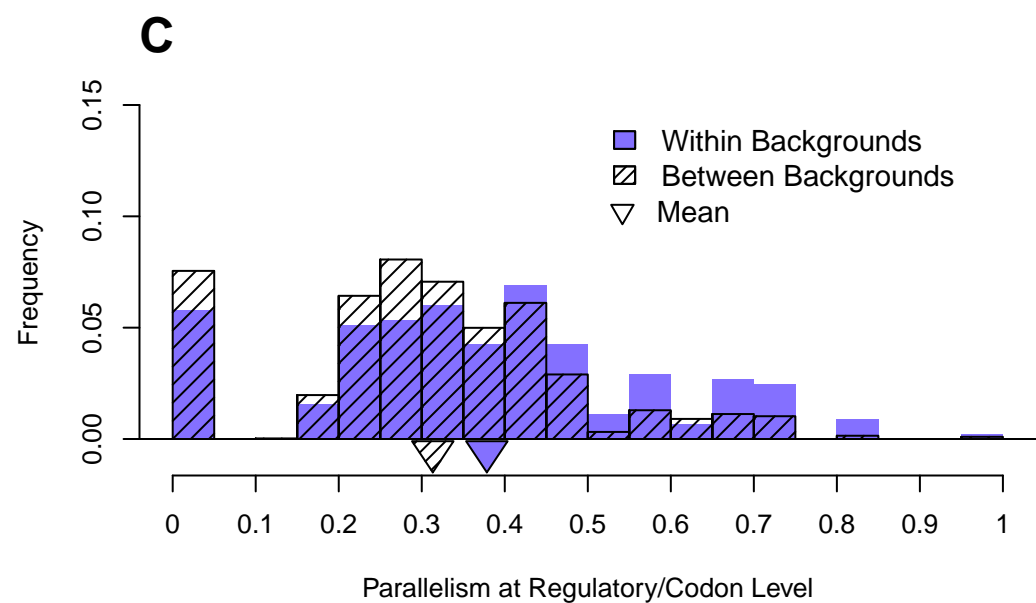

Supplement: Supplemental Information 3 [file peerj-04-2227-s003.zip › Markdown_detailed_anlaysis_code_and_data_minor_revisions/across_back_parallelism_match.pdf]

**A**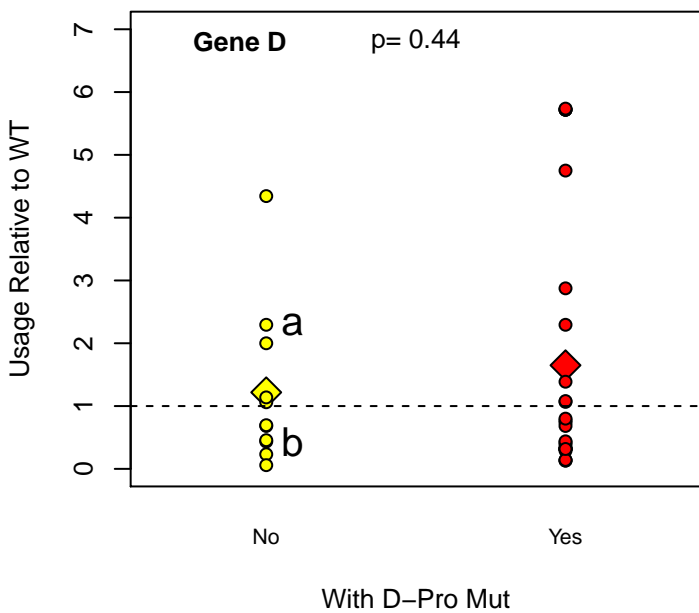**B**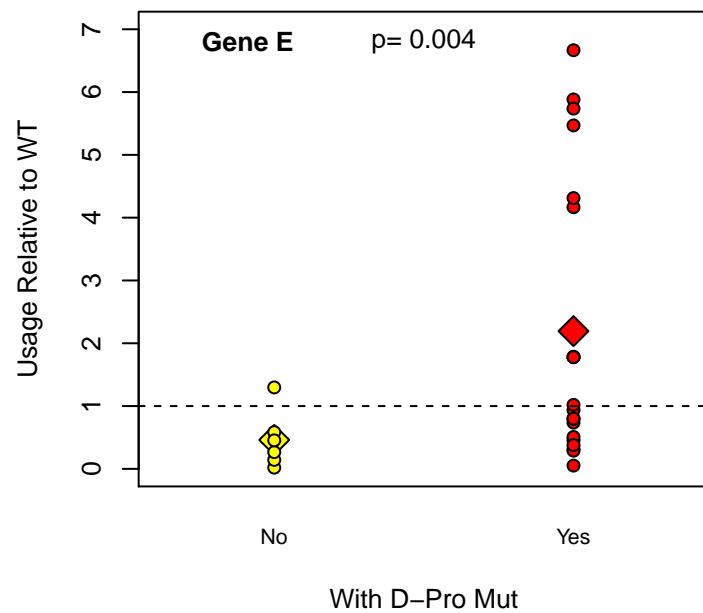**C**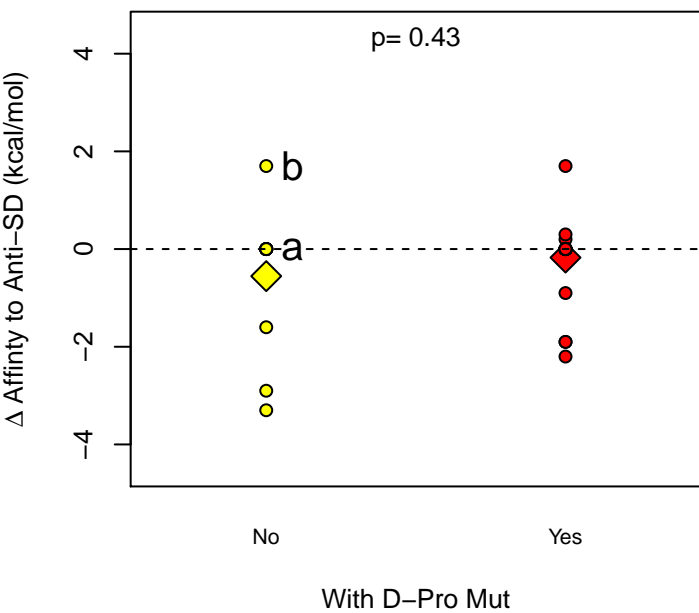**D**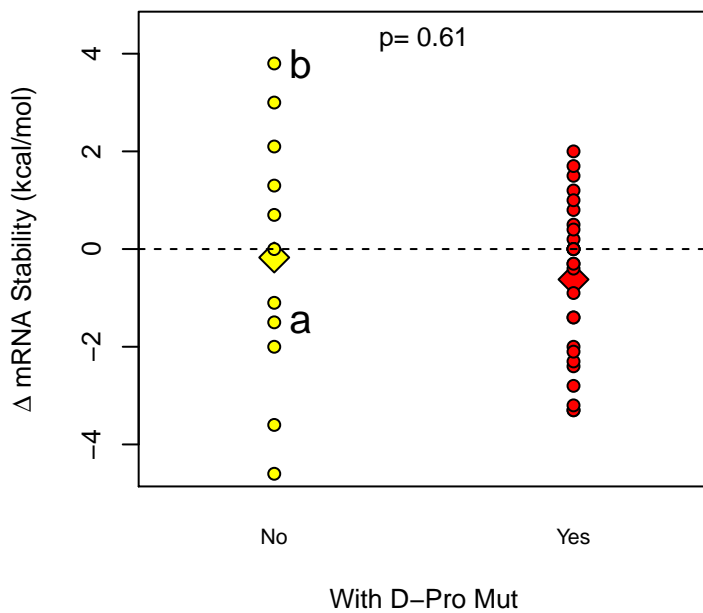

Supplement: Supplemental Information 3 [file peerj-04-2227-s003.zip › Markdown_detailed_anlaysis_code_and_data_minor_revisions/Delay_mech_dotplots.pdf]

**A**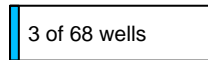

3864.A.G

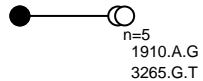**B**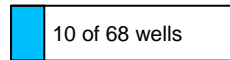

2520.C.T

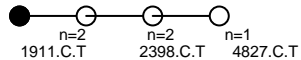**C**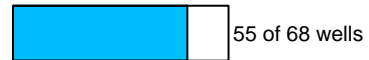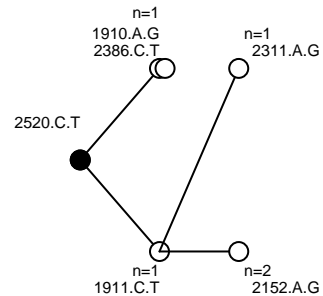

Supplement: Supplemental Information 3 [file peerj-04-2227-s003.zip › Markdown_detailed_anlaysis_code_and_data_minor_revisions/Example_tree_figure_March1_2016]

**A**

3 of 68 wells

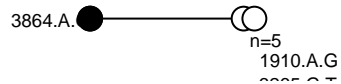**B**

11 of 68 wells

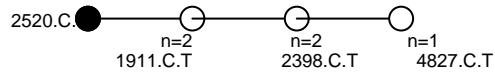**C**

12 of 68 wells

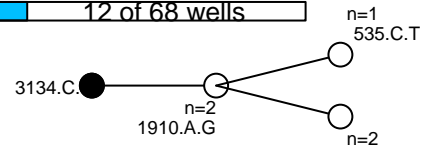**D**

41 of 68 wells

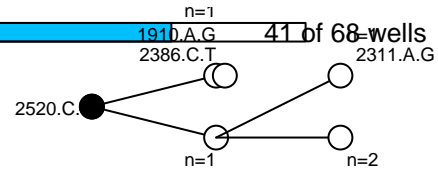

Supplement: Supplemental Information 3 [file peerj-04-2227-s003.zip › Markdown_detailed_anlaysis_code_and_data_minor_revisions/Example_tree_figure_May11_2016]

**A**

3 of 68 wells

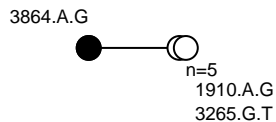**B**

18 of 68 wells

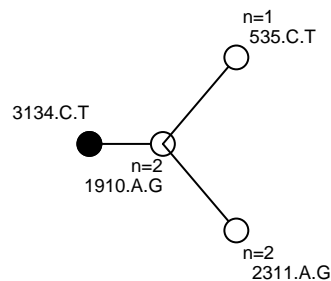**C**

5 of 68 wells

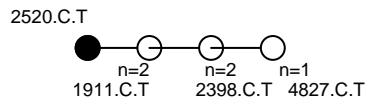**D**

41 of 68 wells

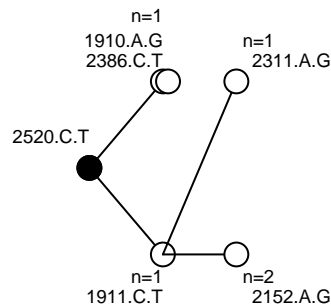

Supplement: Supplemental Information 3 [file peerj-04-2227-s003.zip › Markdown_detailed_anlaysis_code_and_data_minor_revisions/Example_tree_figure_May11_2016.pdf]

**A**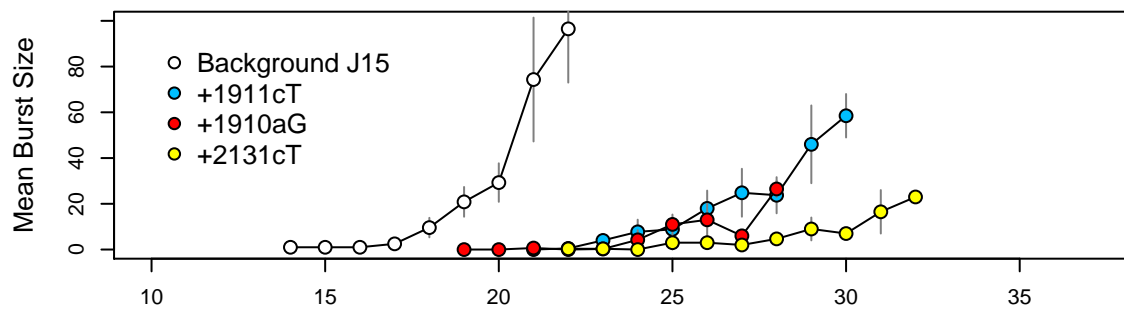**B**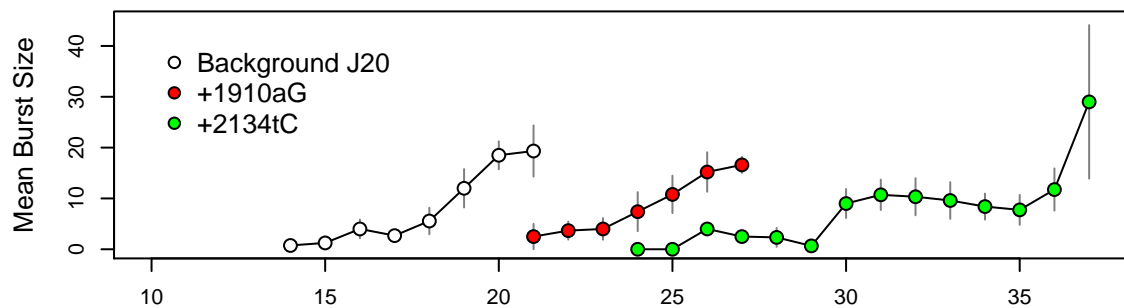**C**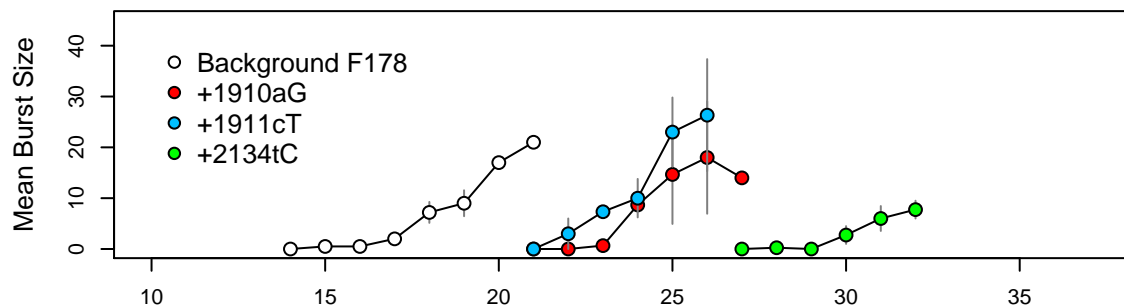**D**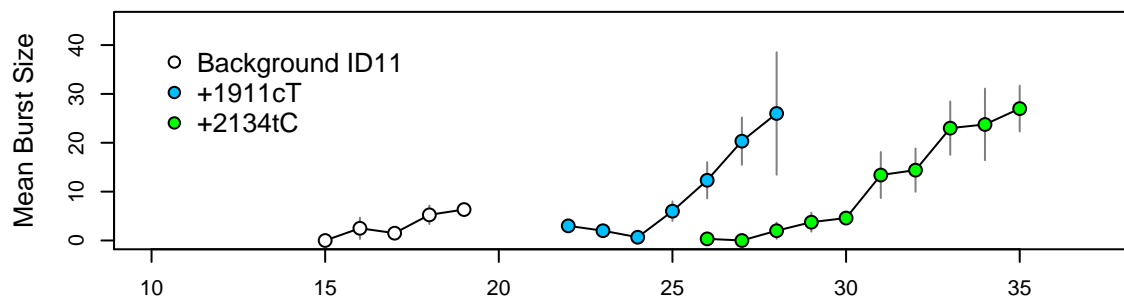

Minutes After Exposure to Host Cells

Supplement: Supplemental Information 3 [file peerj-04-2227-s003.zip › Markdown_detailed_anlaysis_code_and_data_minor_revisions/Lysis_Time_Figure.pdf]

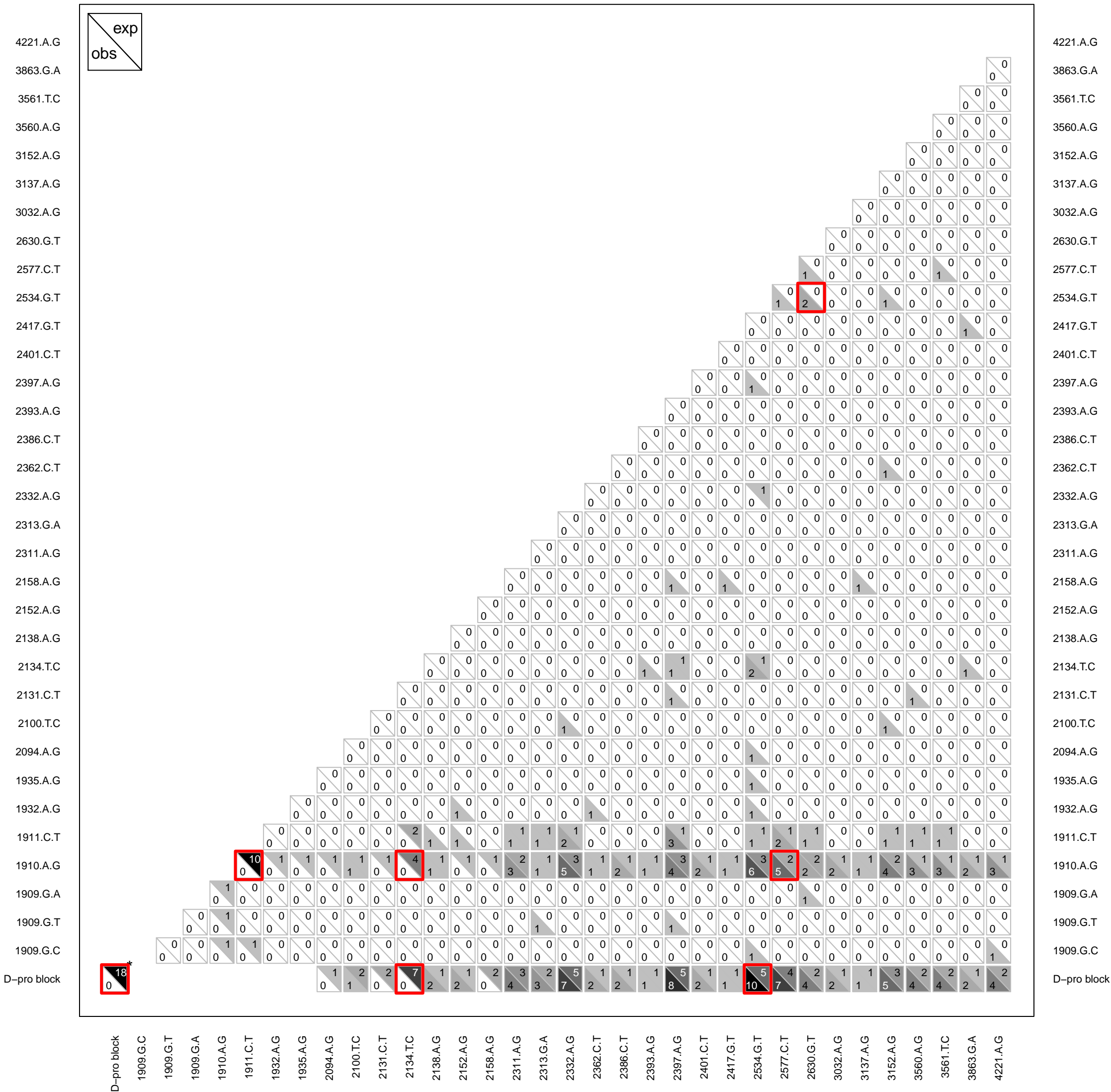

Supplement: Supplemental Information 3 [file peerj-04-2227-s003.zip › Markdown_detailed_anlaysis_code_and_data_minor_revisions/mutation_co-occurence_3.pdf]

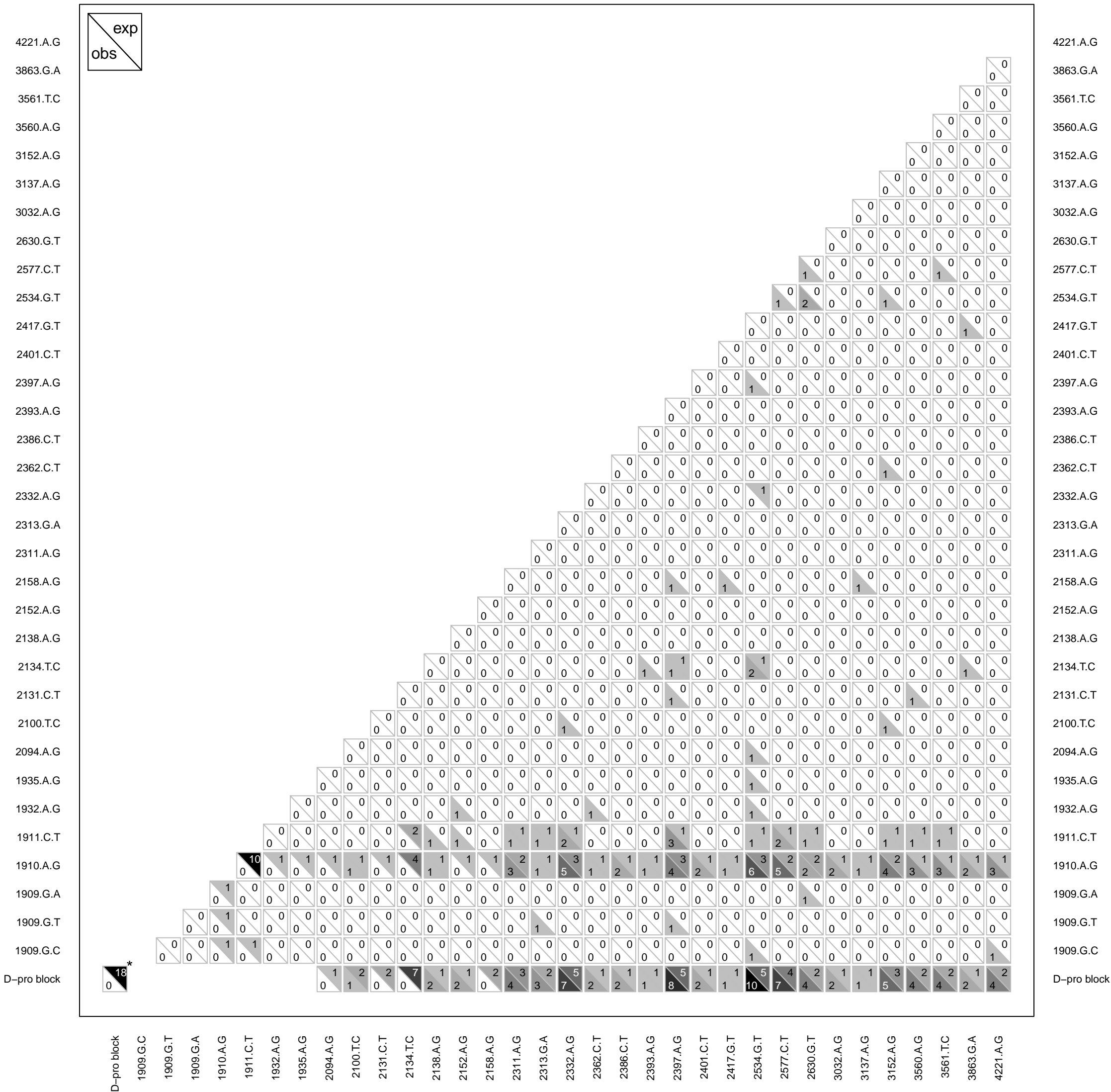

Supplement: Supplemental Information 3 [file peerj-04-2227-s003.zip › Markdown_detailed_anlaysis_code_and_data_minor_revisions/Mutations_by_background_figure_March_2016.pdf]

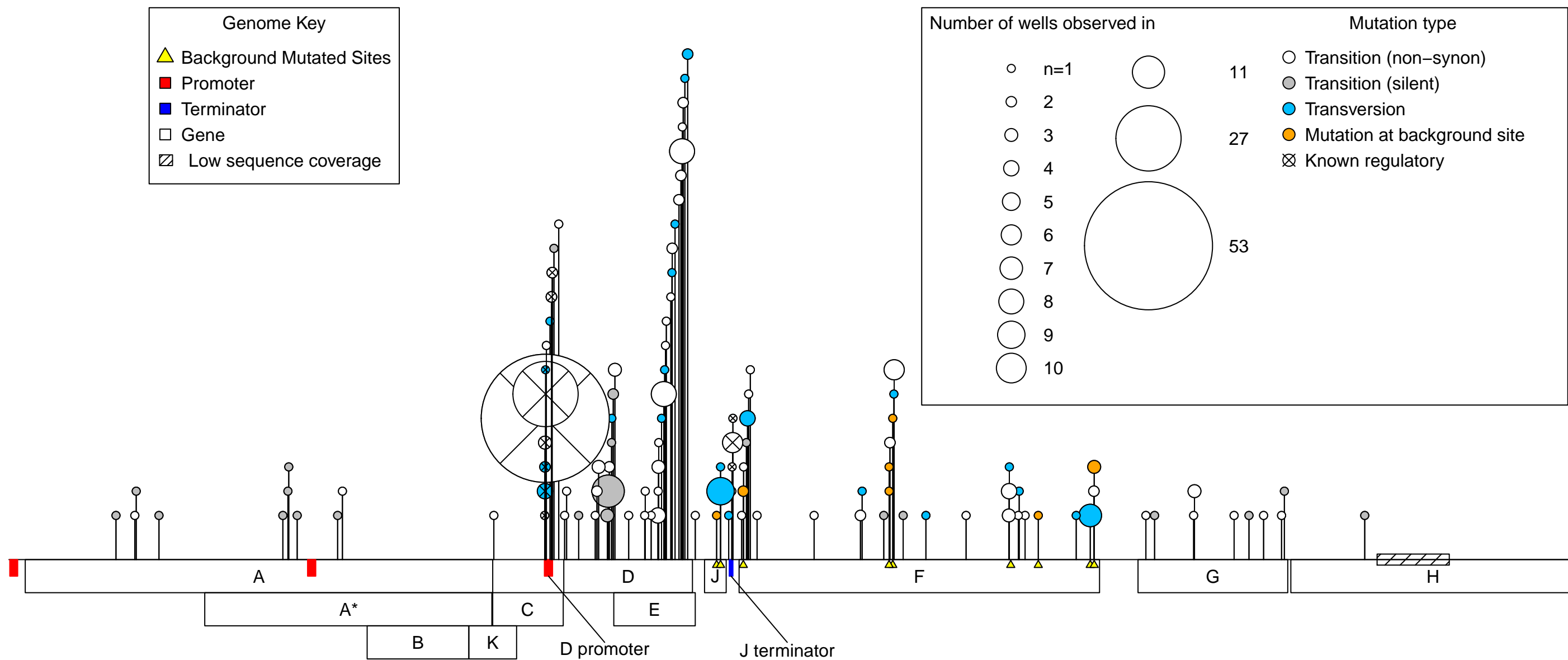

Supplement: Supplemental Information 3 [file peerj-04-2227-s003.zip › Markdown_detailed_anlaysis_code_and_data_minor_revisions/pin_plot_March_2016]

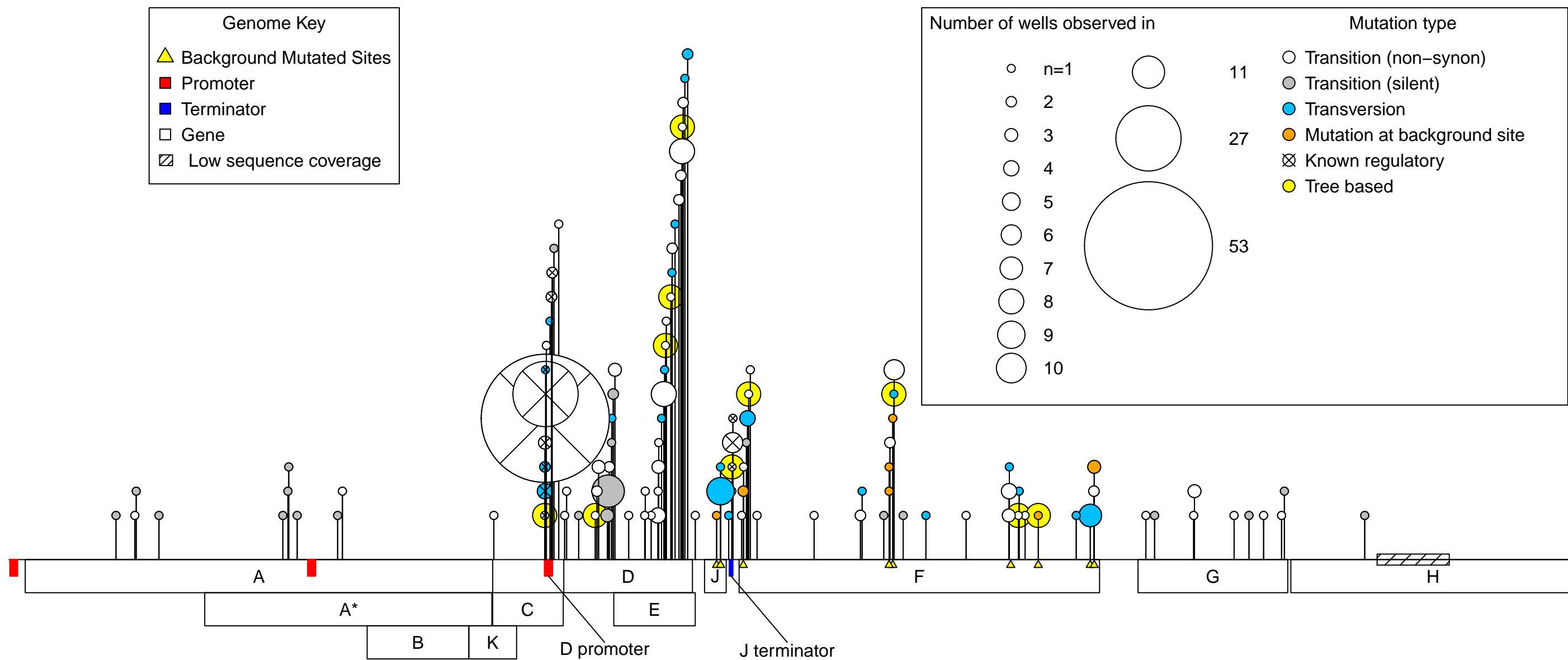

Supplement: Supplemental Information 3 [file peerj-04-2227-s003.zip › Markdown_detailed_anlaysis_code_and_data_minor_revisions/Pin_plot_tree_based.pdf]

**F178 alpha\_1B**

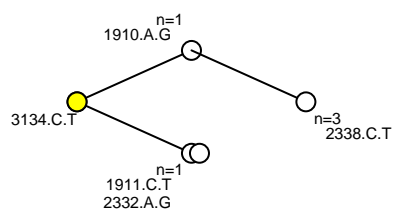

**F178 alpha\_3D**

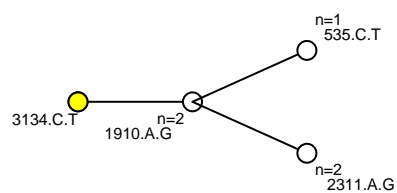

**F178 alpha\_5F**

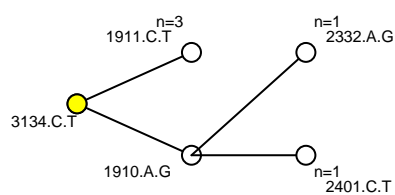

**F178 beta\_2E**

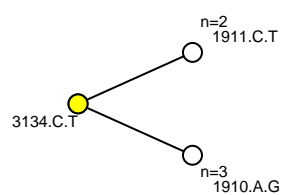

**F178 gamma\_4A**

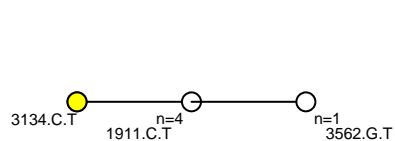

**F178 gamma\_8E**

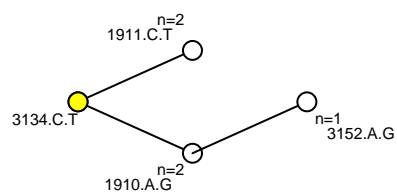

**F178 gamma\_6C**

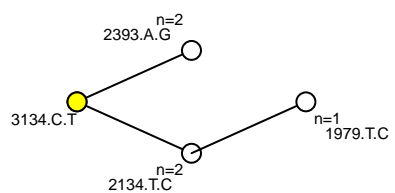

**F178 beta\_7B**

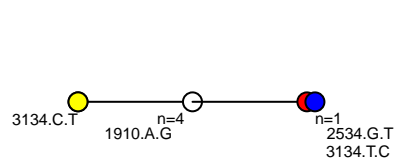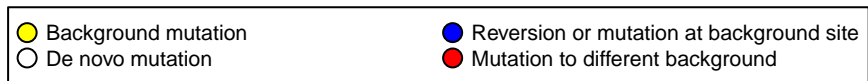

Supplement: Supplemental Information 3 [file peerj-04-2227-s003.zip › Markdown_detailed_anlaysis_code_and_data_minor_revisions/trees_each_well_F178.pdf]

**F182 alpha\_8A**

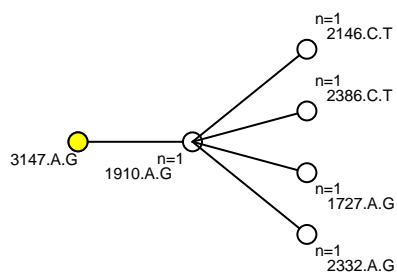

**F182 alpha\_1D**

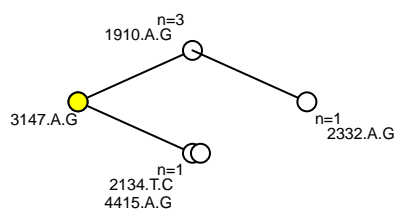

**F182 alpha\_3F**

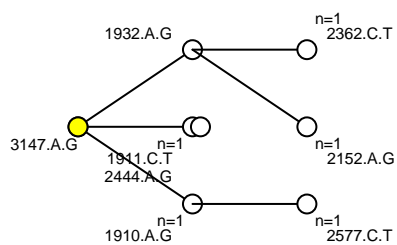

**F182 beta\_5B**

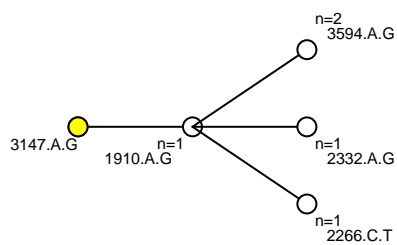

**F182 beta\_7D**

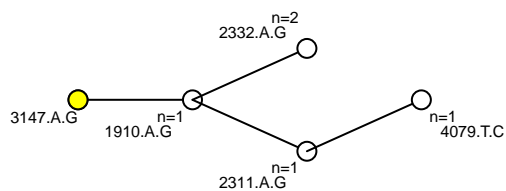

**F182 gamma\_2A**

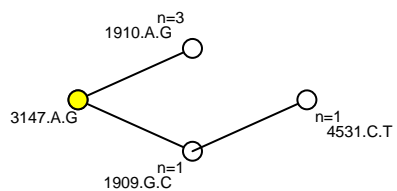

**F182 gamma\_6E**

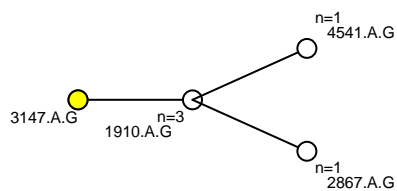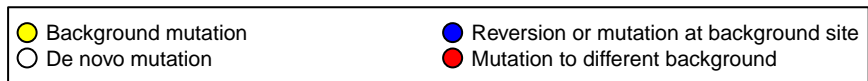

Supplement: Supplemental Information 3 [file peerj-04-2227-s003.zip › Markdown_detailed_anlaysis_code_and_data_minor_revisions/trees_each_well_F182.pdf]

**F322 alpha\_3B**

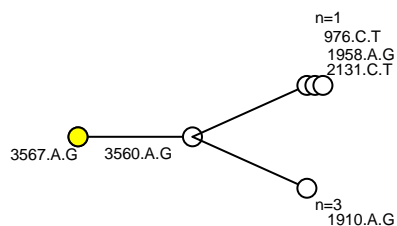

**F322 alpha\_5D**

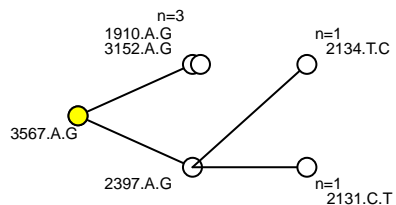

**F322 alpha\_7F**

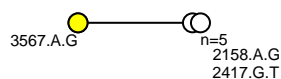

**F322 beta\_2C**

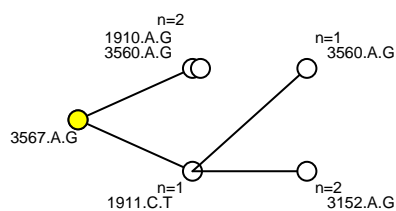

**F322 beta\_4E**

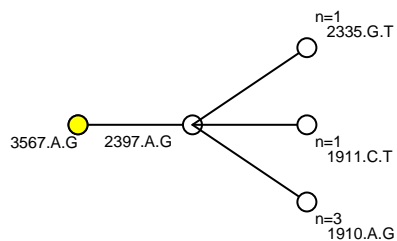

**F322 gamma\_6A**

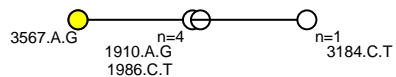

**F322 gamma\_8C**

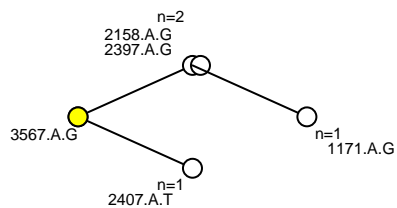

**F322 gamma\_1F**

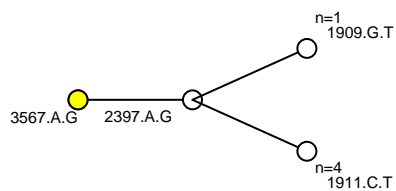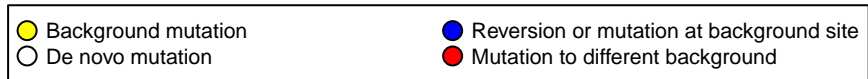

Supplement: Supplemental Information 3 [file peerj-04-2227-s003.zip › Markdown_detailed_anlaysis_code_and_data_minor_revisions/trees_each_well_F322.pdf]

**F355 alpha\_6A**

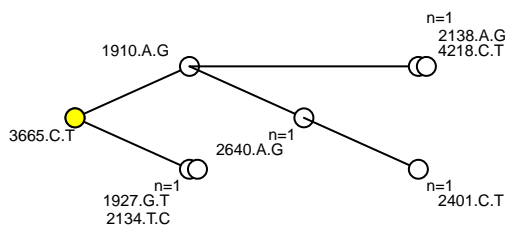

**F355 alpha\_8C**

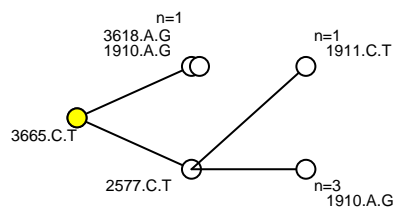

**F355 alpha\_1F**

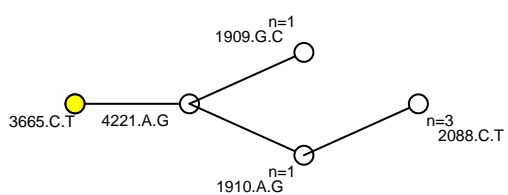

**F355 beta\_3B**

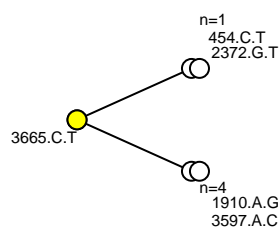

**F355 beta\_5D**

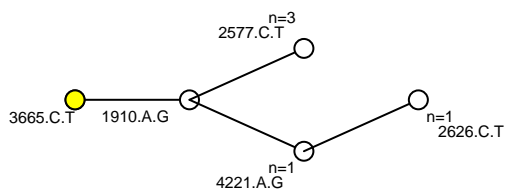

**F355 gamma\_2C**

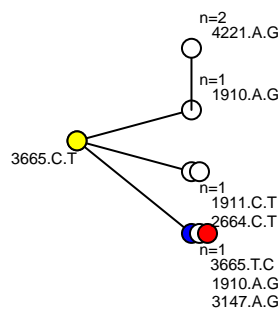

**F355 gamma\_4E**

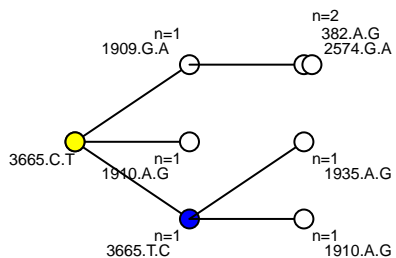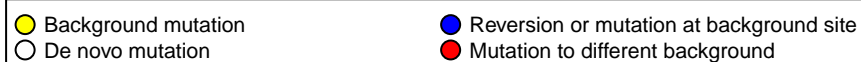

Supplement: Supplemental Information 3 [file peerj-04-2227-s003.zip › Markdown_detailed_anlaysis_code_and_data_minor_revisions/trees_each_well_F355.pdf]

**F416 gamma\_3D**

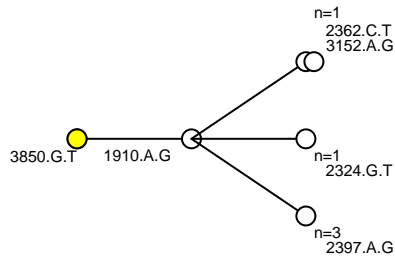

**F416 alpha\_7B**

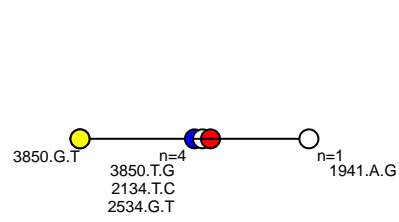

**F416 alpha\_2E**

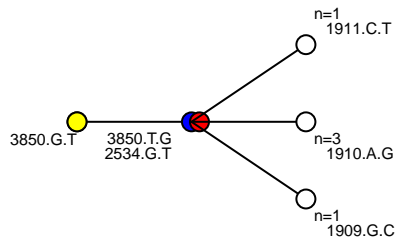

**F416 beta\_4A**

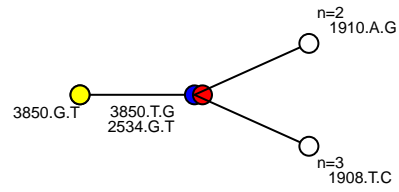

**F416 beta\_6C**

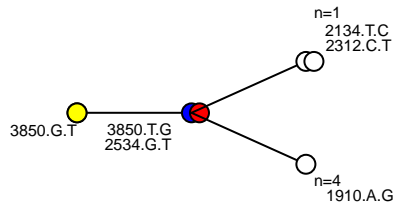

**F416 beta\_8E**

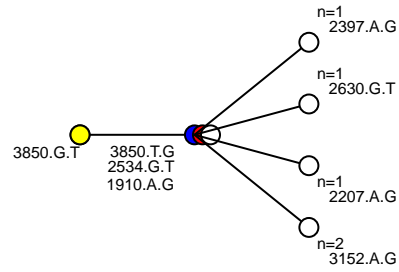

**F416 gamma\_1B**

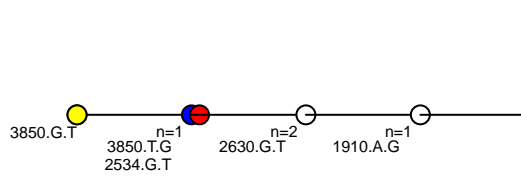

**F416 gamma\_5F**

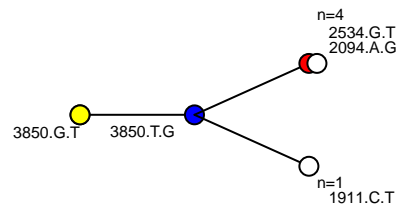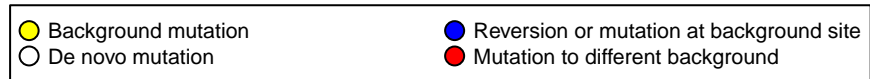

Supplement: Supplemental Information 3 [file peerj-04-2227-s003.zip › Markdown_detailed_anlaysis_code_and_data_minor_revisions/trees_each_well_F416.pdf]

**F421 alpha\_2C**

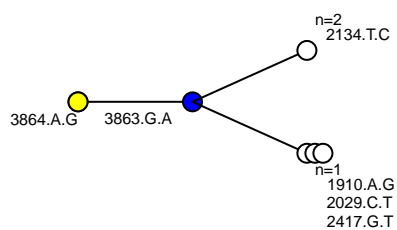

**F421 alpha\_4E**

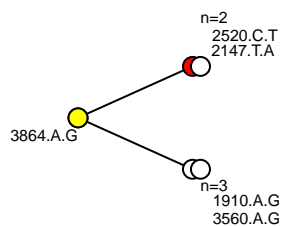

**F421 beta\_6A**

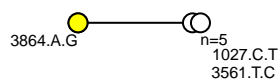

**F421 beta\_8C**

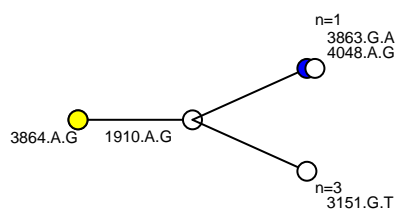

**F421 beta\_1F**

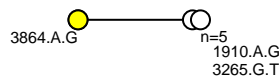

**F421 gamma\_3B**

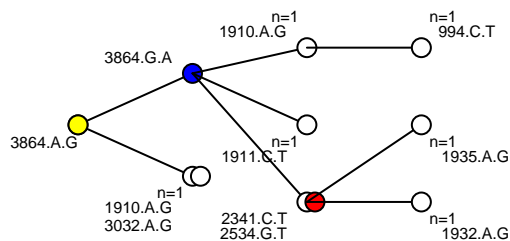

**F421 gamma\_5D**

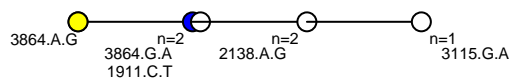

**F421 gamma\_7F**

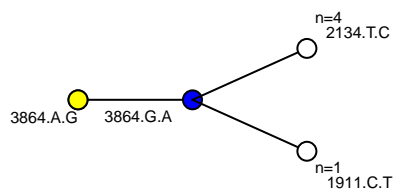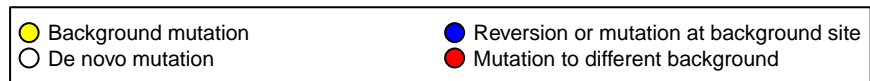

Supplement: Supplemental Information 3 [file peerj-04-2227-s003.zip › Markdown_detailed_anlaysis_code_and_data_minor_revisions/trees_each_well_F421.pdf]

**F5 alpha\_2A**

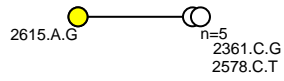

**F5 beta\_1D**

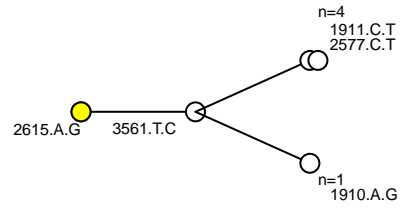

**F5 beta\_3F**

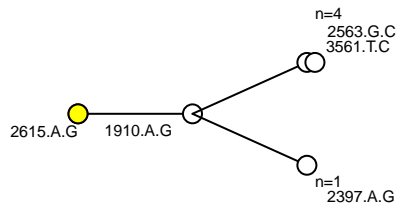

**F5 gamma\_7D**

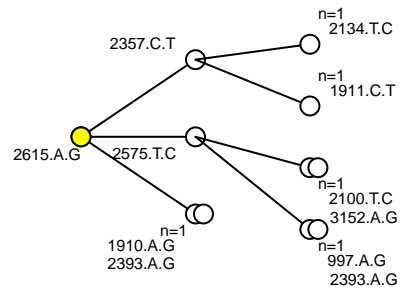

**F5 beta\_8A**

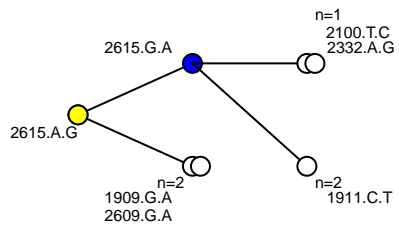

**F5 gamma\_5B**

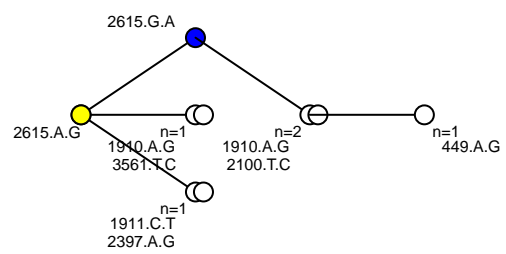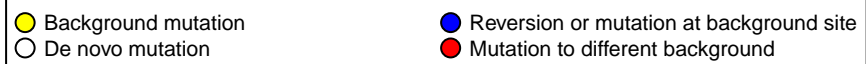

Supplement: Supplemental Information 3 [file peerj-04-2227-s003.zip › Markdown_detailed_anlaysis_code_and_data_minor_revisions/trees_each_well_F5.pdf]

**J15 alpha\_4A**

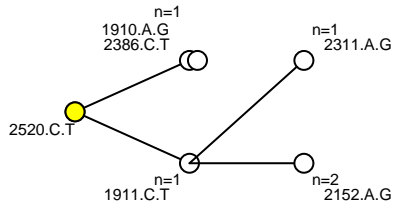

**J15 alpha\_6C**

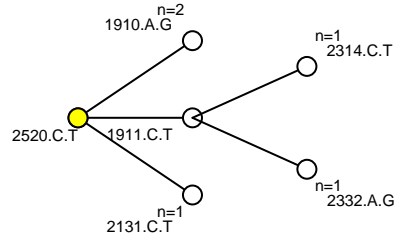

**J15 alpha\_8E**

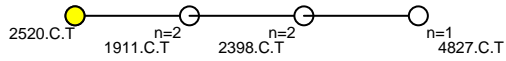

**J15 beta\_1B**

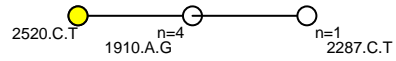

**J15 beta\_3D**

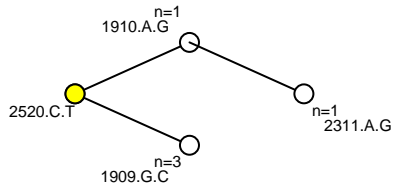

**J15 beta\_5F**

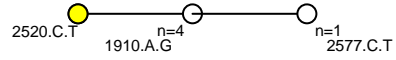

**J15 gamma\_7B**

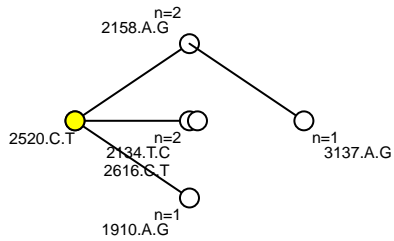

**J15 gamma\_2E**

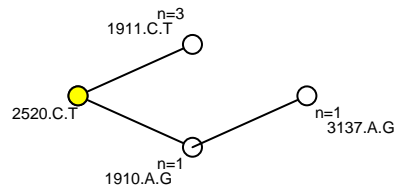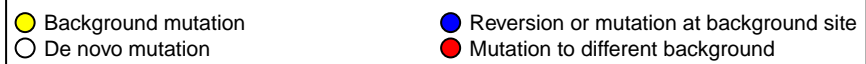

Supplement: Supplemental Information 3 [file peerj-04-2227-s003.zip › Markdown_detailed_anlaysis_code_and_data_minor_revisions/trees_each_well_J15.pdf]

**J20 alpha\_5B**

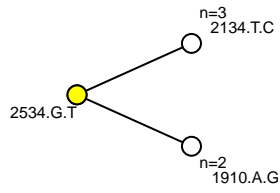

**J20 alpha\_7D**

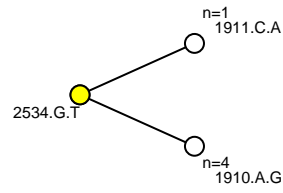

**J20 beta\_2A**

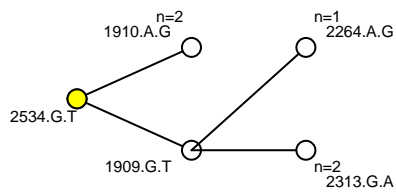

**J20 beta\_4C**

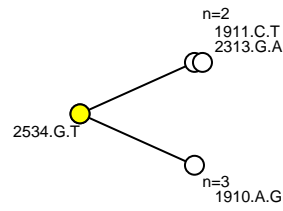

**J20 beta\_6E**

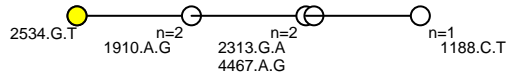

**J20 gamma\_8A**

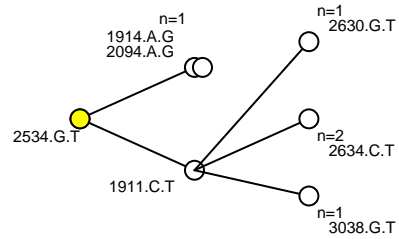

**J20 gamma\_1D**

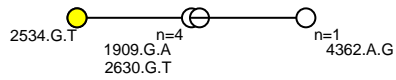

**J20 gamma\_3F**

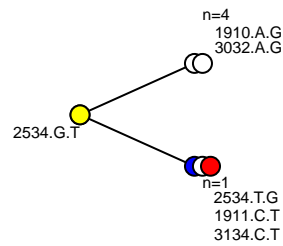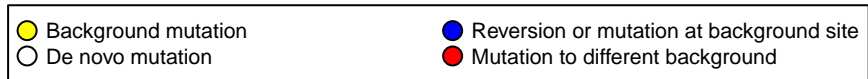

Supplement: Supplemental Information 3 [file peerj-04-2227-s003.zip › Markdown_detailed_anlaysis_code_and_data_minor_revisions/trees_each_well_J20.pdf]

**A****DNA Mutations (n=112)**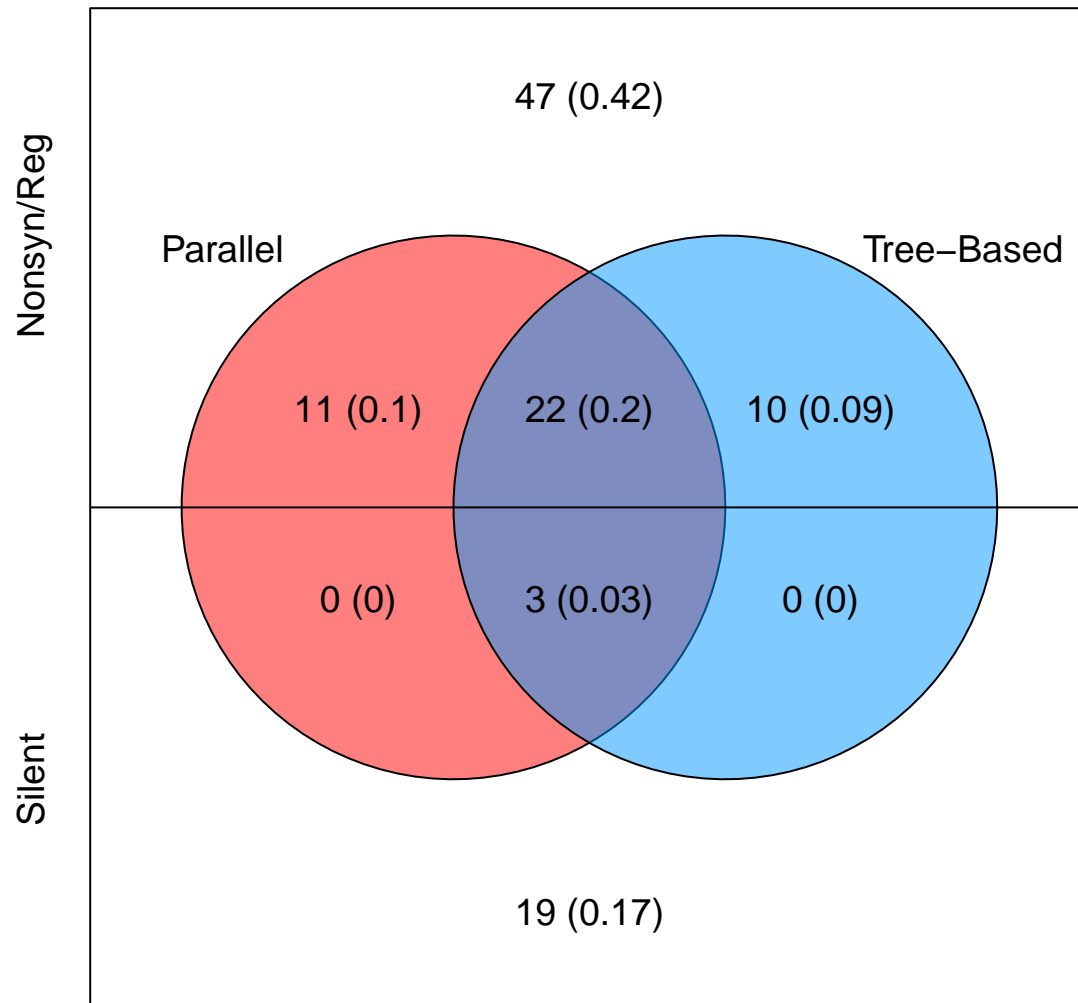**B****Mutational Events (n=281)**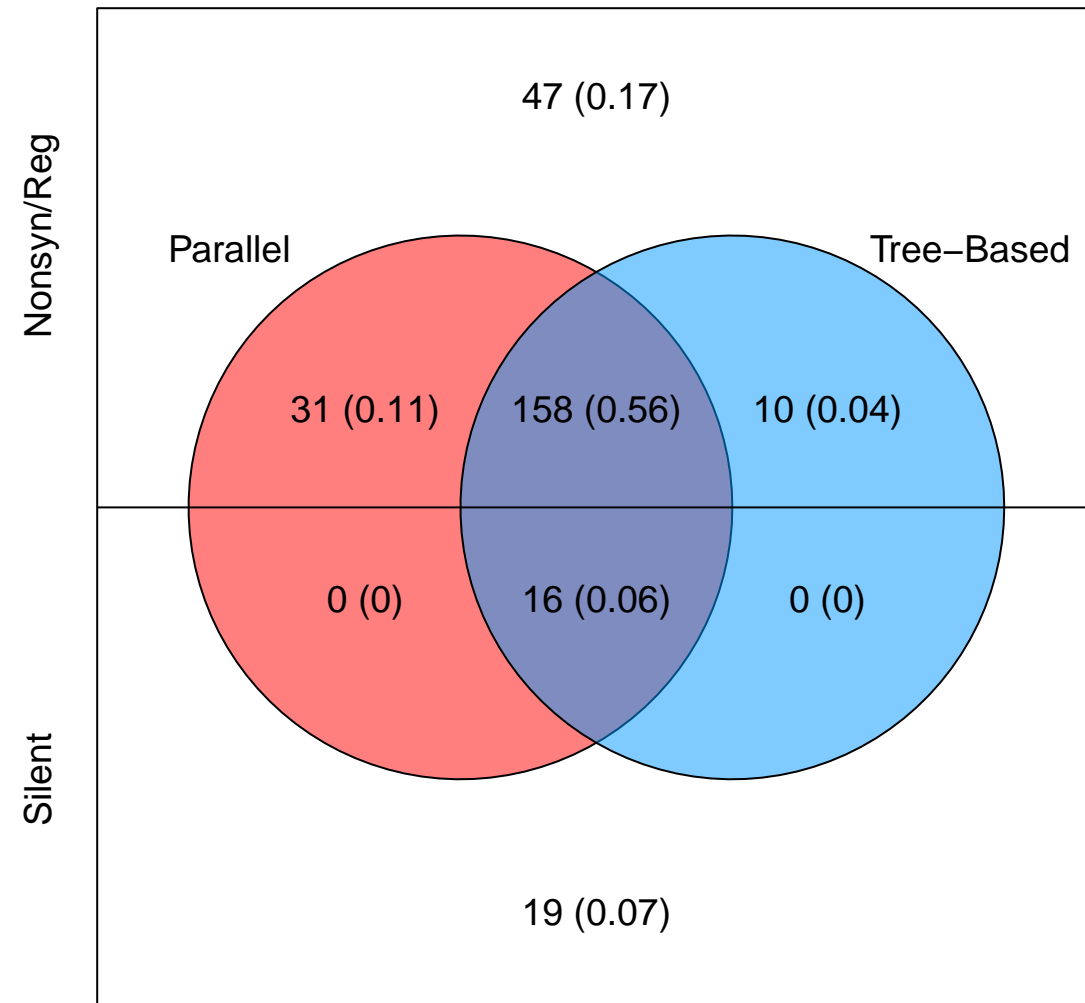

Supplement: Supplemental Information 3 [file peerj-04-2227-s003.zip › Markdown_detailed_anlaysis_code_and_data_minor_revisions/Venn_Adaptive_Muts_2_split_panels.pdf]

**A**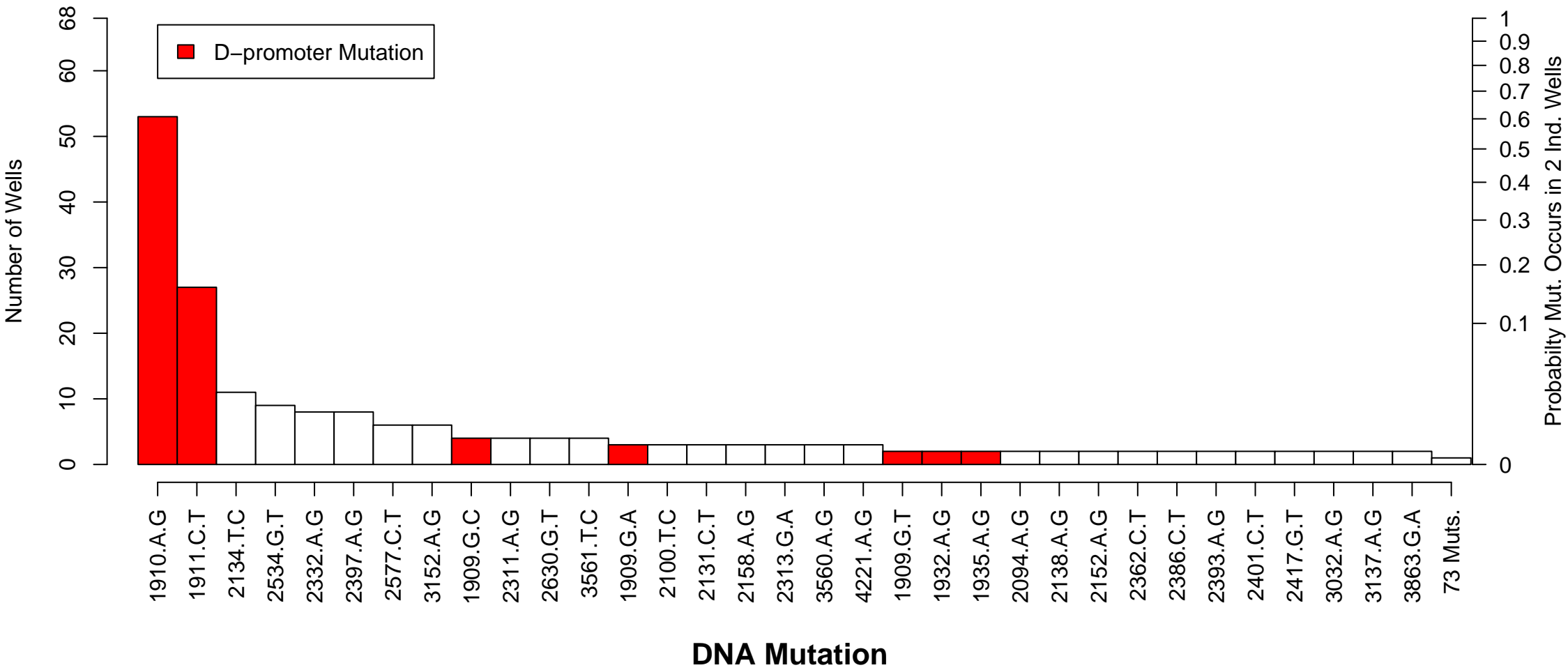**B**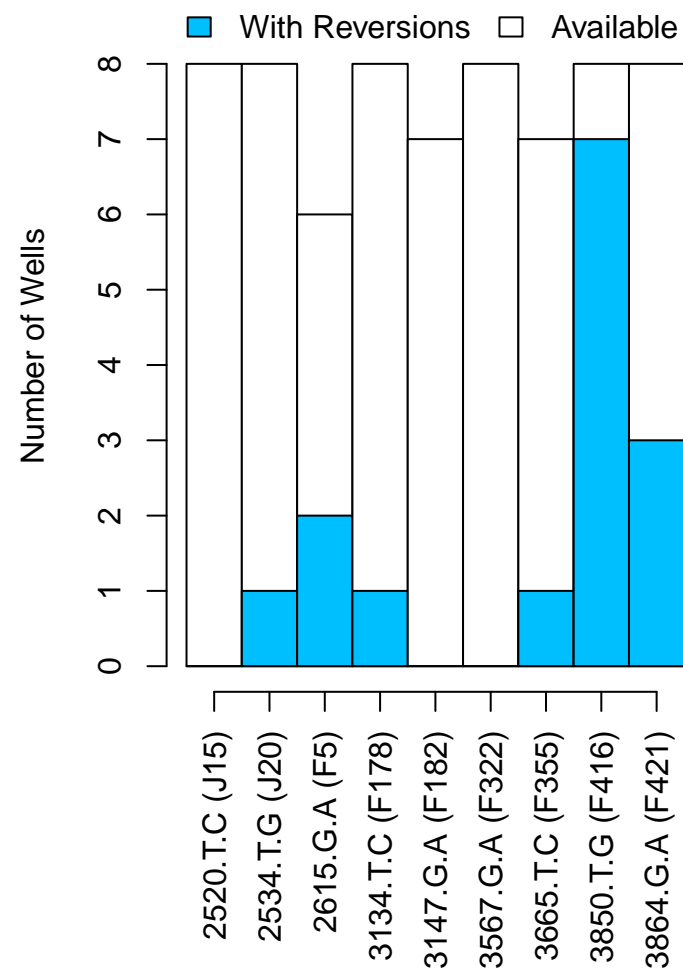

Supplement: Supplemental Information 3 [file peerj-04-2227-s003.zip › Markdown_detailed_anlaysis_code_and_data_minor_revisions/Well_count_dist_by_mutation_3.pdf]
